# Potent Inducers of Paraptosis through Electronic Tuning of Hemicyanine Electrophiles

**DOI:** 10.1021/jacs.5c07109

**Published:** 2025-08-26

**Authors:** Juan F. Tamez-Fernández, Craig F. Steven, Jade Nguyen, Pablo Rivera-Fuentes

**Affiliations:** † Department of Chemistry, 27217 University of Zurich, CH-8057, Zurich, Switzerland; ‡ Institute of Chemical Sciences and Engineering, 27218 École Polytechnique Fédérale de Lausanne, CH-1015, Lausanne, Switzerland

## Abstract

Paraptosis is a distinct form of programmed cell death characterized by cytoplasmic vacuolization, mitochondrial swelling, and endoplasmic reticulum (ER) dilation, offering an alternative to apoptosis for therapeutic applications. In this study, we identified a hemicyanine derivative that is a potent paraptosis inducer in two cancer cell lines. This compound triggers hallmark paraptotic features, including ER swelling, mitochondrial morphological changes, increased superoxide production, and caspase-independent cell death. This activity is dependent on the ability of the probe to modify thiols covalently. Proteomic analysis using a biotinylated, activity-based probe revealed Sec23 homologue A and GDP-dissociation inhibitor alpha as potential targets implicated in paraptosis activation. This lead compound already displayed some degree of selectivity, exemplified by its minimal interaction with well-known nucleophilic protein targets such as protein disulfide isomerases. These findings establish the hemicyanine chemical family as a promising scaffold for paraptosis research and suggest potential as a therapeutic lead for diseases where traditional apoptosis pathways are dysregulated.

## Graphical abstract

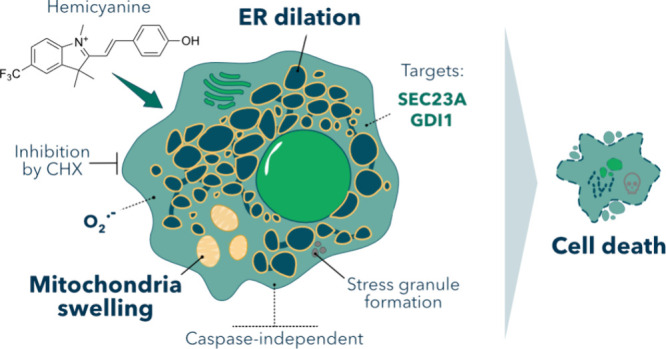

## Introduction

Paraptosis, a form of programmed cell death, is distinguished by unique morphological and biochemical features that set it apart from apoptosis and necrosis.^1,2^ Unlike apoptosis, which involves cellular shrinkage, DNA fragmentation, and the formation of apoptotic bodies, paraptosis is characterized by extensive cytoplasmic vacuolization, mitochondrial swelling, and the absence of caspase activation or DNA fragmentation.^1,2^ This form of cell death is typically accompanied by protein and Ca^2+^ homeostasis disruption and activation of the unfolded protein response of the endoplasmic reticulum (UPR^ER^). Paraptosis is usually mediated through signaling pathways involving mitogen-activated protein kinases (MAPKs).^2,3^ Despite the heterogeneity of its activation mechanisms, a few studies have reported specific paraptosis-inducing targets like the insulin-like growth factor I receptor (*IGF1R*),^1,2^ GDP-dissociation inhibitor beta (*GDI2*),^4^ and UBP10 (*USP10*), a member of the ubiquitin-specific protease family of cysteine proteases.^5^ Recently, VCP/p97 (*SVIP*) has been recognized as a paraptosis target and its inhibition triggers ER vacuolation.^6^

Understanding paraptosis is crucial for elucidating its role in development, disease, and therapy,^7–10^ as it provides alternative mechanisms for cell death that could be harnessed for therapeutic interventions, particularly in cancer treatment where traditional apoptosis pathways are often dysregulated. Unlike other cell death mechanisms (i.e., apoptosis, necrosis, ferroptosis, and pyroptosis), paraptosis is controlled, noninflammatory, and independent of apoptotic pathways.^10,11^ These characteristics make it especially appealing for therapeutic applications, particularly in cancer and neurodegenerative disorders. Its unique morphological and molecular features complement other forms of cell death, broadening the scope of therapeutic strategies.

Exploiting paraptosis for therapeutic purposes requires the development of effective chemical probes. Various compounds have been reported to induce paraptosis in cancer cell lines at concentrations ranging from 0.5 to 40 μM, with incubation periods between 6 and 72 h^12–25^ (Table S1). However, the high concentrations and prolonged exposure required to trigger paraptosis highlight the low potency of current compounds. Moreover, many of these compounds are natural products with complex chemical structures, which complicates structure–activity relationship studies. Therefore, identifying new, structurally simple molecules with improved potency and selectivity targeting paraptosis-related proteins is crucial to explore the therapeutic potential of this cell-death mechanism. In this context, hemicyanine compounds emerge as promising candidates due to their structural simplicity, biological compatibility, and ability to interact selectively with biomolecules and respond to changes in the cellular environment.^26–28^ Their strong fluorescence, good biocompatibility, and ability to penetrate cells make them ideal candidates for bioimaging and biosensing.^26^ Additionally, their photosensitive properties enable their use in photodynamic and photothermal therapies, contributing to advancements in cancer treatment and other disease-targeted applications.^29^

We recently observed that hemicyanine compounds (**1–5**, Figure 1A) induced varying levels of cytoplasmic vacuolation and exhibited significant cytotoxicity. Among them, compound **2** induced extensive vacuolation and cytotoxicity across three different cell lines. Herein, we demonstrate that compound **2** triggers hallmark features of paraptosis, including mitochondrial swelling, ER dilation, increased superoxide production, and reactivity with cysteine residues. Proteomic analysis identified Sec23 homologue A (*SEC23A*) and GDP-dissociation inhibitor alpha (*GDI1*) as targets of compound **2** and potential activators of this nonapoptotic cell death pathway. These findings establish compound **2** as a potent activator of paraptosis and highlight its potential as a lead compound for further development.

**Figure 1:**
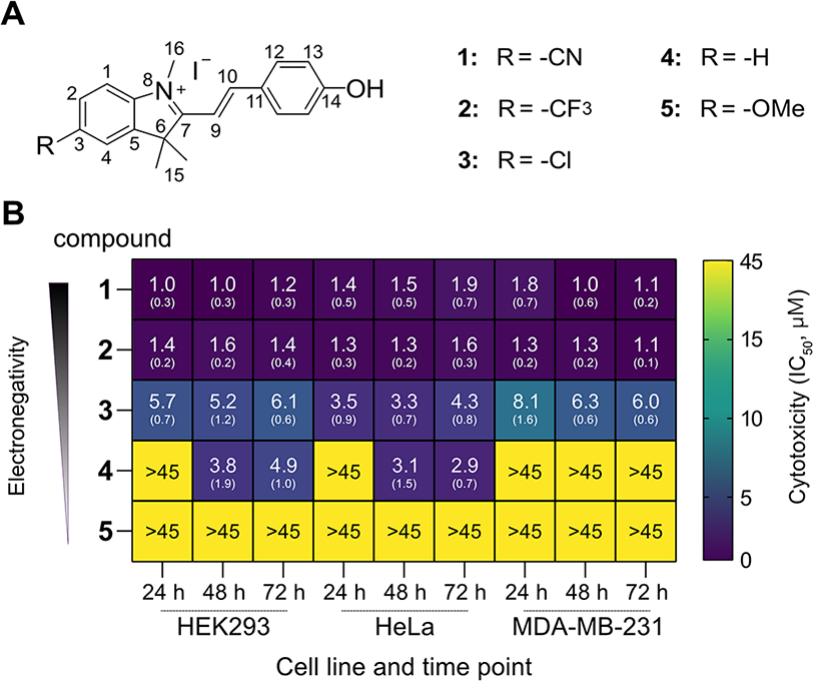
Cytotoxicity of hemicyanines **1**–**5**. (A) Chemical structures of compounds **1**–**5**. (B) Heat map of IC_50_ values of cytotoxicity assays of compounds **1**–**5** in HEK293, HeLa, and MDA-MB-231 cells. Cells were incubated with compounds at concentrations from 44 nM to 45 μM. Cell viability was assessed by MTT assay at 24, 48, and 72 h. IC_50_ was calculated by taking as 100% of viability the DMSO-treated cells. The experiments were performed in triplicate for each compound. Means and standard deviation (in parentheses) are indicated in each panel. The grayscale gradient bar indicates variations in the R substituent’s electronegativity across the different compounds.

## Results

### The Cytotoxicity of Hemicyanines Across Cell Types Correlates with Electrophilicity

We hypothesized that hemicyanine compounds **1**–**5** would display varying degrees of electrophilicity owing to the electronic properties of the substituent R in the indolenine fragment (Figure 1A). These compounds were synthesized following a published procedure^30^ and characterized by ^1^H-,^13^C NMR, and HRMS (Figures S17–S49). The half-maximal inhibitory concentration (IC_50_) of these compounds was determined in HEK293 (embryonic kidney), HeLa (cervical cancer), and MDA-MB-231 (triple-negative breast cancer) human cells at different time points by the dimethylthiazol tetrazolium (MTT) assay (Figure 1B; Figure S1). The toxicity of the compounds was significantly influenced by changes in the electronegativity of the R substituent (C3).

As the electronegativity of the substituent increased, the compounds displayed higher cytotoxicity, showing a consistent trend across all cell lines. Given that hemicyanines are Michael acceptors, we interpreted this trend as an indication that the toxicity of these compounds is related to their electrophilicity. Thus, compounds **1**-**5** may act as covalent ligands for nucleophilic residues in proteins such as cysteine (vide infra).^31,32^ Additionally, we investigated the influence of oxygen and nitrogen atoms at the C14 position. To explore this effect, we synthesized compound **2-NH**_**2**_ (Figure S2) by replacing the hydroxyl (−OH) group in compound **2** with an amine (−NH_2_) group. The nitrogen-containing compound **2-NH**_**2**_ is less cytotoxic than its oxygen-containing analog **2** (Figure S2). This difference could be explained considering that the oxygen in compound **2**—with a p*K*_a_ of 7.0 ± 0.2 (Figure S3)—is largely deprotonated at neutral pH, giving an overall neutral molecule. In contrast, compound **2-NH**_**2**_ cannot be deprotonated within the biologically relevant range of pH values (Figure S3) and has a positive charge. This change in total charge decreases the membrane permeability of **2-NH**_**2**_ and affects its subcellular localization, as confirmed by fluorescence microscopy and colocalization analysis (Figure S4).

We observed that even though compound **1** should be more electrophilic than compound **2**, it exhibits similar cytotoxicity across the three cell lines (Figure 1B). This seemingly counterintuitive observation can be explained by considering that with increased electrophilicity, compound **1** is also more prone to reaction with glutathione (GSH) than compound **2** (Figure S5). Thus, compound **2** displays sufficient electrophilicity to potentially modify protein residues, but not enough to be substantially trapped by abundant GSH in the cell. Given its potency and favorable properties, we chose compound **2** to characterize the biological effects and identify the molecular targets of this class of electrophilic compounds.

### Compound **2** Activates a Nonapoptotic Cell Death Mechanism

Cytoplasmic vacuolation has been widely associated with nonapoptotic mechanisms.^33^ To determine whether compound **2** triggered a nonapoptotic mechanism, we evaluated apoptosis induction by measuring caspase-3/7 activation in HeLa cells treated with compound **2**. Caspase-3/7 cleavage was detected using the fluorescent probe CellEvent^TM^ Caspase-3/7 Green, alongside the nucleic acid stain SYTO^TM^ Deep Red. Staurosporine (STS), a well-established apoptosis inducer, served as the positive control, while DMSO was used as the negative control, and zVAD-FMK as a pan-caspase inhibitor.

Cells treated with compound **2** exhibited no significant caspase-3/7 activation compared to the controls (Figure 2A and Figure S6). As expected, cells pretreated with zVAD-FMK followed by STS did not display caspase activation (Figure 2A and Figure S6). In the same experiment, we evaluated cell shrinkage and nuclear fragmentation, both well-characterized hallmarks of apoptosis. Our findings demonstrated that, in contrast to STS treatment, compound **2** did not induce nuclear fragmentation or cellular shrinkage (Figure 2B). Overall, these results indicate that compound **2** does not trigger caspase activation, nuclear fragmentation, or cellular shrinkage, suggesting the involvement of nonapoptotic pathways.

**Figure 2:**
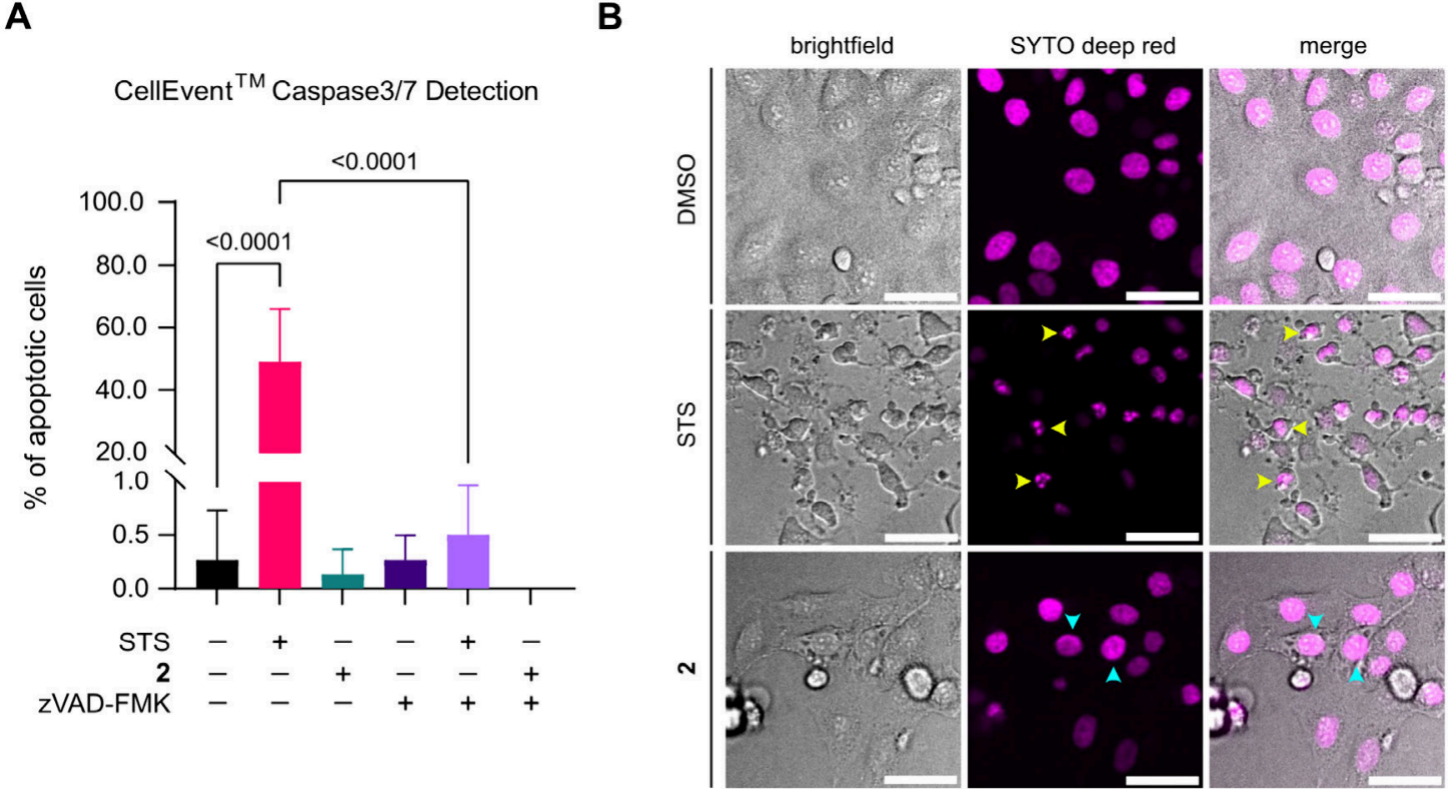
Apoptosis evaluation in live HeLa cells treated with **2**. (A) Quantification of apoptotic cells treated with STS or **2**, with and without pan-caspase inhibitor (zVAD-FMK), compared to DMSO control. Cells were incubated with DMSO (0.1%, 1 h), STS (2 μM, 4 h), or compound **2** (2 μM, 2 h). Where indicated, the cells were preincubated with zVAD-FMK (20 μM, 2 h), then coincubated with DMSO, STS, or **2** as mentioned above. Cells were stained with SYTO^TM^ Deep Red nucleic acid stain (1X, 30 min, 37 °C) and CellEvent^TM^ Caspase-3/7 green reagent (3 μM, 30 min, 37 °C) before imaging. Means are plotted and error bars represent standard mean error. Measurements were carried out for at least 200 cells from biological triplicates. *P*-values are indicated for each treatment comparison and were calculated using a one-way ANOVA test with a Tukey comparison. (B) Nuclear fragmentation of HeLa cells treated with STS and **2**, compared to DMSO control. Cells were incubated with DMSO (0.1%, 1 h), STS (2 μM, 4 h), and compound **2** (2 μM, 2 h). Cells were stained with SYTODeep Red nucleic acid stain (1X, 30 min, 37 °C) before imaging. Each panel displays the fluorescence of SYTO^TM^ Deep Red nucleic acid stain (excitation: 640 nm, 100 ms, 4.3 mW). The cells with cytoplasmic vacuoles are pointed by the light blue arrows and the fragmented nuclei by the yellow arrows. Scale bars = 50 μm.

### Compound **2** Induces Morphological Changes in the ER and Mitochondria

We next evaluated the morphological changes of the ER and mitochondria in the three cell lines induced by compound **2**. HEK293, HeLa, and MDA-MB-231 cells were incubated with **2** for 2 h and stained with ER-Tracker^TM^ Green. Cytoplasmic vacuolation was observed in all three cell lines (Figure 3A). Additionally, the vacuole membranes were stained with ER-Tracker^TM^ Green, suggesting their ER origin. To further evaluate the origin of the vacuoles, we transfected the cells with a fluorescent protein targeted to the ER lumen using the KDEL retention sequence (KDEL-mTurquoise2) and incubated them with compound **2** for 2 h. Confocal time-lapse microscopy revealed that these vacuoles contain the fluorescent protein, confirming that they originate from ER dilation (Figure 3B).

**Figure 3:**
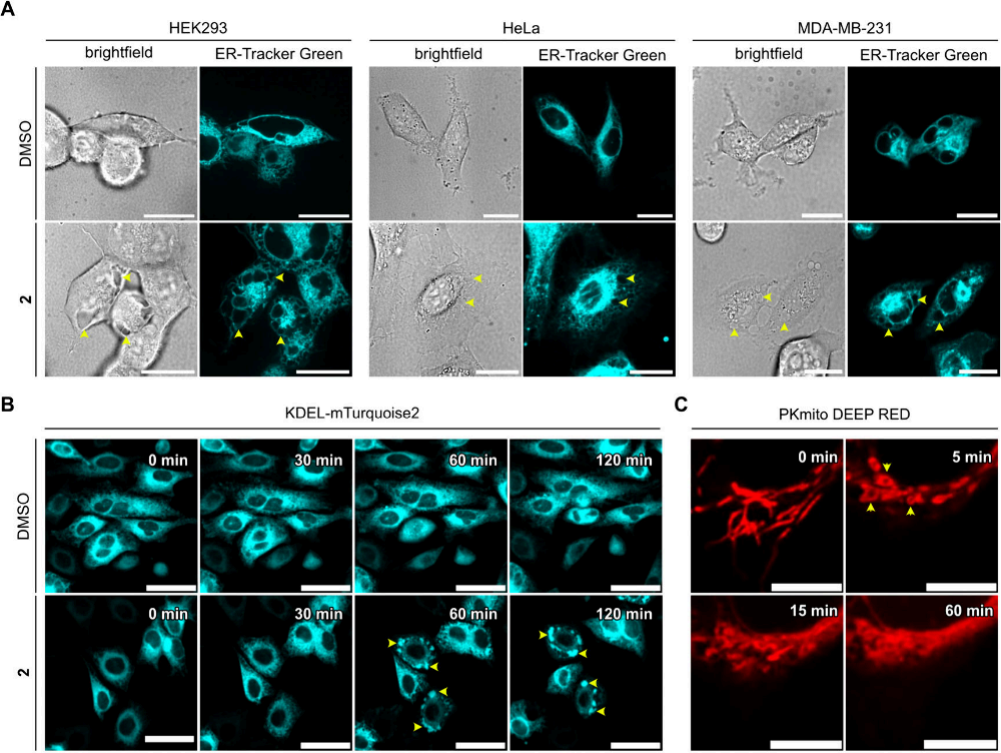
Changes in the morphology of the ER and mitochondria induced by hemicyanine **2**. (A) Induction of cytoplasmic vacuolation by **2** in HEK293, HeLa, and MDA-MB-231 cells. Cells were incubated with ER Tracker^TM^ Green (1 μM, 1 h), and incubated with DMSO (0.1%, 2 h) or **2** (2 μM, 2 h). Each panel shows the fluorescence of ER Tracker^TM^ Green (excitation: 488 nm, 300 ms, 0.7 mW). The ER vacuoles are pointed by the yellow arrows. Scale bars = 20 μm. (B) Time-lapse imaging of the induction of cytoplasmic vacuolation by **2** in HeLa cells. Cells were transfected with KDEL-mTurquoise2 2 days before imaging and incubated with DMSO (0.1%, 2 h) or **2** (2 μM, 2 h). Each panel displays the fluorescence of KDEL-mTurquoise2 (excitation: 445 nm, 400 ms, 1.9 mW) at different time points. The ER vacuoles are pointed by the yellow arrows. Scale bars = 50 μm. (C) Morphological changes of mitochondria induced by **2** in HeLa cells. Cells were preincubated with PKmito DEEP RED (1X, 1 h), and coincubated with **2** (2 μM, 2 h). Each panel displays fluorescence of PKmito DEEP RED (excitation: 640 nm, 100 ms, 3.7 mW) at different time points. The donut-shaped mitochondria are pointed by the yellow arrows. Scale bars = 10 μm.

ER dilation is often accompanied by mitochondria swelling.^1,2^ Thus, we monitored morphological changes in mitochondria and their role in vacuole formation by incubating compound **2** with HeLa cells stained with the mitochondrial probe PKmito DEEP RED (Figure 3C). The morphology changes were evident starting at 5 min after the addition of compound **2** and a characteristic donut-shaped morphology was observed (Figure 3C), indicating mitochondrial stress.^34,35^ After 15 min, the PKmito DEEP RED probe began to leak to the cytosol, indicating mitochondrial depolarization.^36^ Importantly, we observed that vacuolation did not involve mitochondria as they surround the vacuoles and no PKmito DEEP RED was observed in the vacuoles (Figure S7).

### Compound **2** Increases Superoxide Production

Reactive oxygen species (ROS) are primarily generated in mitochondria and they have an important role in cell death mechanisms.^37^ Previous studies have shown that superoxide (O_2_^•‑^) is closely associated with paraptosis induction.^2,8,25^ Therefore, we assessed the production of this radical anion in HeLa cells treated with compound **2**. We used a derivative of the fluorescent probe HKSOX-1 (HKSOX-1*) to detect intracellular O_2_^•–^ as shown in Figure 4A.^38^ We employed Antimycin A, an inhibitor of cytochrome c reductase that increases the intracellular concentration of O_2_^•–^ as the positive control, and DMSO as the negative control. Cells treated with compound **2** displayed a significantly higher O_2_^•–^ accumulation compared to cells treated with the controls and this increase was dose-dependent (Figure 4B, 4C, and S9).

**Figure 4:**
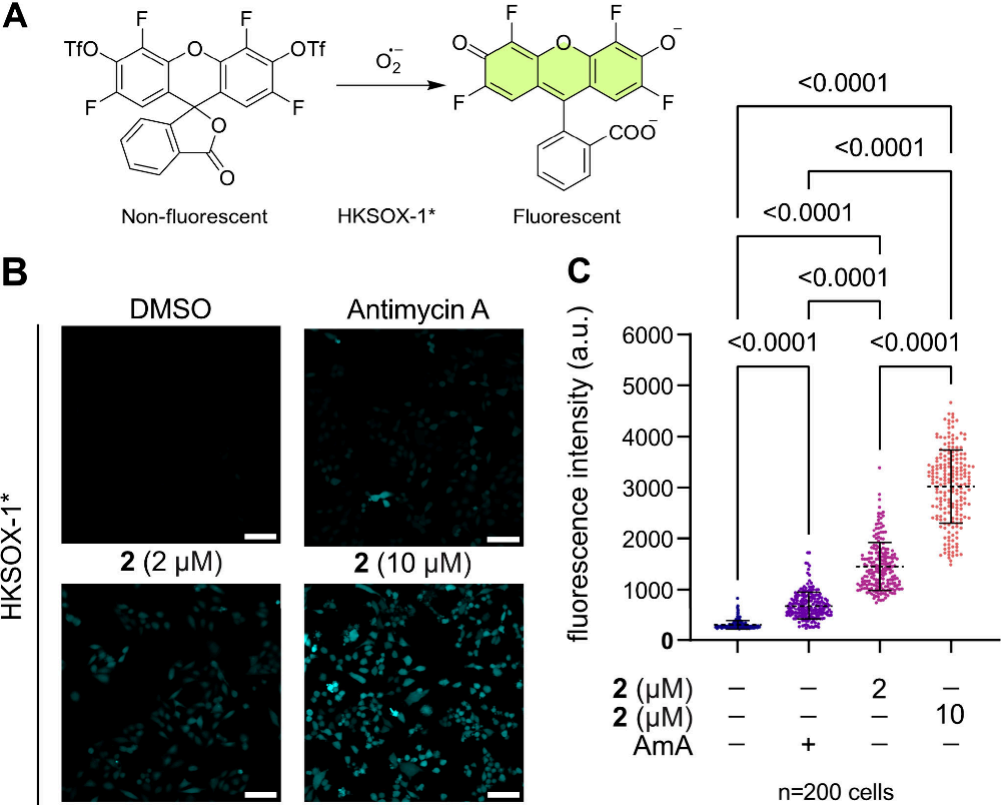
O_2_^**•–**^ production measurement in live HeLa cells treated with **2**. (A) Chemical structure of O_2_^•–^ probe HKSOX-1* and activation mechanism. (B) The cells were incubated with HKSOX-1* (10 μM, 30 min) before imaging and incubated with DMSO (0.1%, 1 h), compound **2** (2 and 10 μM, 1 h), and Antimycin A (10 μM, 1 h). Each panel displays the fluorescence of HKSOX-1* (excitation: 488 nm, 300 ms, 0.7 mW). Scale bars = 100 μm. (C) Quantification of fluorescence intensity compared to DMSO control of cells treated as described in (B). Means are plotted, and error bars represent standard deviation. Measurements were carried out for 200 cells across biological triplicates, but only one replicate is shown in the plot. *P*-values are indicated for each treatment comparison and were calculated using a one-way ANOVA test with a Tukey comparison.

### Compound **2** Induces ER Vacuolation Independently of Caspases and O_2_^•–^ and Is Inhibited by Cycloheximide

Paraptosis is a process that does not involve caspase activation and the vacuolation process can be suppressed by the protein synthesis inhibitor cycloheximide (CHX).^2^ We evaluated the effect of the pan-caspase inhibitor zVAD-FMK and CHX on vacuole formation induced by compound **2**. This effect was evaluated by confocal microscopy in HeLa cells expressing KDEL-mTurquoise2. DMSO was used as a negative control and compound **2** in the absence of inhibitors as a positive control. The percentage of vacuolated cells was quantified and compared against controls.

After 2 h of treatment with compound **2**, zVAD-FMK (20 μM) was unable to rescue cells from ER vacuolation (Figure 5A and 5B). On the other hand, pretreatment with CHX (20 μM) partially prevented vacuole formation as evidenced by a smaller percentage of vacuolated cells (Figure 5A and 5B). These results confirm that vacuolation is a caspase-independent process and that ER dilation is driven by the accumulation of proteins. However, this response seems to be faster than the onset of the unfolded protein response in the ER (UPR^ER^) because upregulation of BiP/GRP78, an established indicator of UPR^ER^, is not observed at this time point (Figure S10). These observations are consistent with a paraptotic cell-death mechanism.

**Figure 5:**
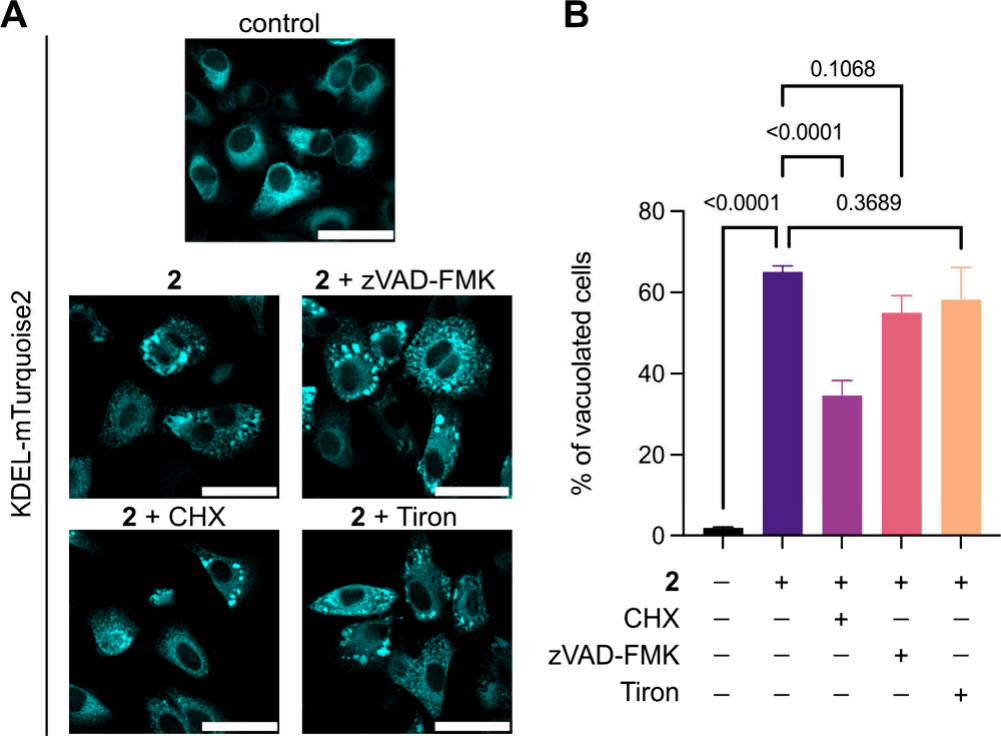
Involvement of caspases, superoxide accumulation, and protein synthesis in vacuole formation. (A) The cells were transfected with KDEL-mTurquoise2 2 days before imaging and then preincubated for 2 h with zVAD-FMK (20 μM), CHX (20 μM), or Tiron (100 μM). After incubation with inhibitors cells were incubated with compound **2** (2 μM, 2 h). DMSO (0.1%, 2 h) was used as a negative control. Each panel shows fluorescence of KDEL-mTurquoise2 (excitation: 445 nm, 400 ms, 1.9 mW). Scale bars = 50 μm. (B) Quantification of vacuolated cells compared to DMSO control of cells treated as described in (A). Means are plotted and error bars represent standard deviation. Measurements were carried out for 300 cells from biological triplicates. *P*-values are indicated for each treatment comparison and were calculated using a one-way ANOVA test with a Tukey comparison. n.s.= nonsignificant difference.

We next questioned whether ER dilation and mitochondrial damage are related. We first assessed the role of O_2_^•‑^ accumulation in the formation of vacuoles by pretreating the cells with the O_2_^•–^ scavenger Tiron (100 μM).^39,40^ Prior treatment with the scavenger did not impact the process of vacuole formation, as indicated in Figure 5. This observation suggests that the increase in O_2_^•–^ does not play a part in the formation of the vacuoles. We next tested whether ER dilation affects mitochondrial morphology. We transfected HeLa cells with the KDEL-mTurquoise2 plasmid and prestained them with PKmito DEEP RED. After a 2 h preincubation with CHX (20 μM), the cells were coincubated with compound **2** for an additional 2 h. Confocal time-lapse microscopy revealed that all cells displayed mitochondrial damage irrespective of whether they contained ER vacuoles or not (Figure S8). This lack of correlation between vacuolation and mitochondrial damage suggests that these processes are independent.

### Compound **2** Binds to Ligandable Cysteines in the Soluble Proteome

Compound **2** is a competent electrophile that can react with thiol-based nucleophiles such as GSH (Figure S5). We next assessed whether this reactivity extends to cysteine residues in proteins by gel-based, qualitative activity-based protein profiling (ABPP).^41,42^ We purified the soluble proteome from the lysate of HeLa cells and treated it with compound **2**. Next, we labeled the ligandable cysteines using the reactive probe ATTO 620 maleimide (Figure S14). A control proteome was treated with ATTO 620 maleimide only. Separation of these proteomes by sodium dodecyl sulfate-polyacrylamide gel electrophoresis (SDS-PAGE) revealed that cysteine labeling by ATTO 620 maleimide (5 μM) is blocked by compound **2** in some proteins in a dose-dependent manner (10–200 μM, Figure 6A). This observation strongly suggests that compound **2** reacts with ligandable cysteine residues in proteins, blocking these sites.

**Figure 6:**
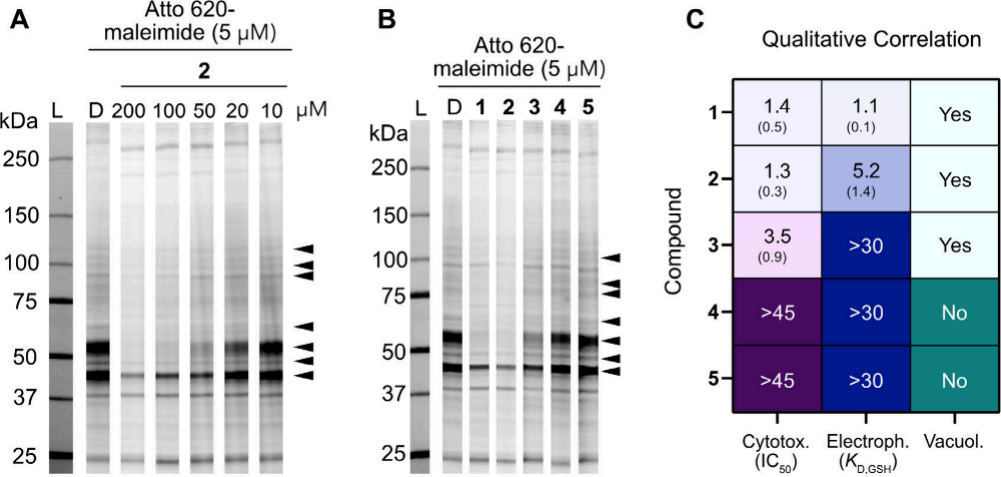
Reactivity of hemicyanines **1**–**5** against cysteine-containing proteins. (A) SDS–PAGE of competitive profiling in the proteome of HeLa cells using ATTO 620 maleimide probe. The soluble proteome of HeLa cells was treated with compound **2** for 2 h at the indicated concentration, followed by labeling with ATTO 620 maleimide (5 μM, 1 h) and analyzed by SDS–PAGE and in-gel fluorescence scanning (detection at λ = 700/50 nm). The black arrows indicate the change in protein band fluorescence. L = Ladder, D = DMSO, kDa = kilodalton. (B) SDS–PAGE of competitive profiling in HeLa cell line proteome using ATTO 620 maleimide probe. Soluble proteome from HeLa cells was treated with compounds **1**–**5** (100 μM, 2 h), followed by labeling with ATTO 620 maleimide (5 μM, 1 h) and analyzed by SDS–PAGE and in-gel fluorescence scanning (detection at λ=700/50 nm). The black arrows indicate the change in protein band fluorescence. L = Ladder, D = DMSO, kDa = kilodalton. (C) Qualitative correlation between cytotoxicity (expressed as IC_50_ in μM), electrophilicity (expressed as *K*_D,GSH_ in mM), and ability of each compound to induce vacuolation. Means and standard deviation (in parentheses) are indicated in each panel. Cytotox. = Cytotoxicity, Electroph. = Electrophilicity, Vacuol. = Vacuolation.

We posited that paraptosis triggered by compound **2** may be correlated to the covalent modification of free thiol groups of intracellular protein targets, leading to the disruption of thiol proteostasis.^15^ To test this hypothesis, we performed the competitive ABPP experiment described before for all hemicyanines **1**–**5** (Figure 6B). This experiment revealed that the extent of cysteine labeling correlates qualitatively with the electrophilicity of the compound, its toxicity, and its ability to induce ER vacuolation (Figure 6C).

A recent study found that nearly all cysteine-modifying covalent ligands induce the formation of stress granules.^43^ To verify whether this is also the case for compound **2**, we transfected HeLa cells with fluorescently labeled G3BP1 protein, a validated nucleator of stress granules.^44^ We observed that indeed compound **2** induced the formation of stress granules, further validating its role as a cysteine covalent modifier (Figure S12).

### Compound **2** Binds to Potential Regulators of Paraptosis

We hypothesized that compound **2** interacts with specific thiol-containing proteins involved in paraptosis. To identify these protein targets, we employed mass-spectrometry-based ABPP (Figure S13)^42,45–47^ and synthesized a biotinylated probe derived from compound **2** (**2-biotin**, Figure 7A). Additionally, to identify targets potentially unrelated to thiol modification, we used a biotinylated probe based on compound **5** (**5-biotin**, Figure 7A), which is a structural analog of **2** with diminished electrophilicity and lack of paraptosis-inducing activity. Finally, to identify general targets of electrophilic probes, we used a biotinylated iodoacetamide (**IA-biotin**, Figure 7A) probe, which is a broad-spectrum cysteine-reactive compound.^48^ Importantly, treatment of cells with **IA** did not induce ER vacuolation (Figure S14) and therefore serves as a valuable control in the identification of targets that are specifically engaged by compound **2** and are involved in vacuolation.

**Figure 7:**
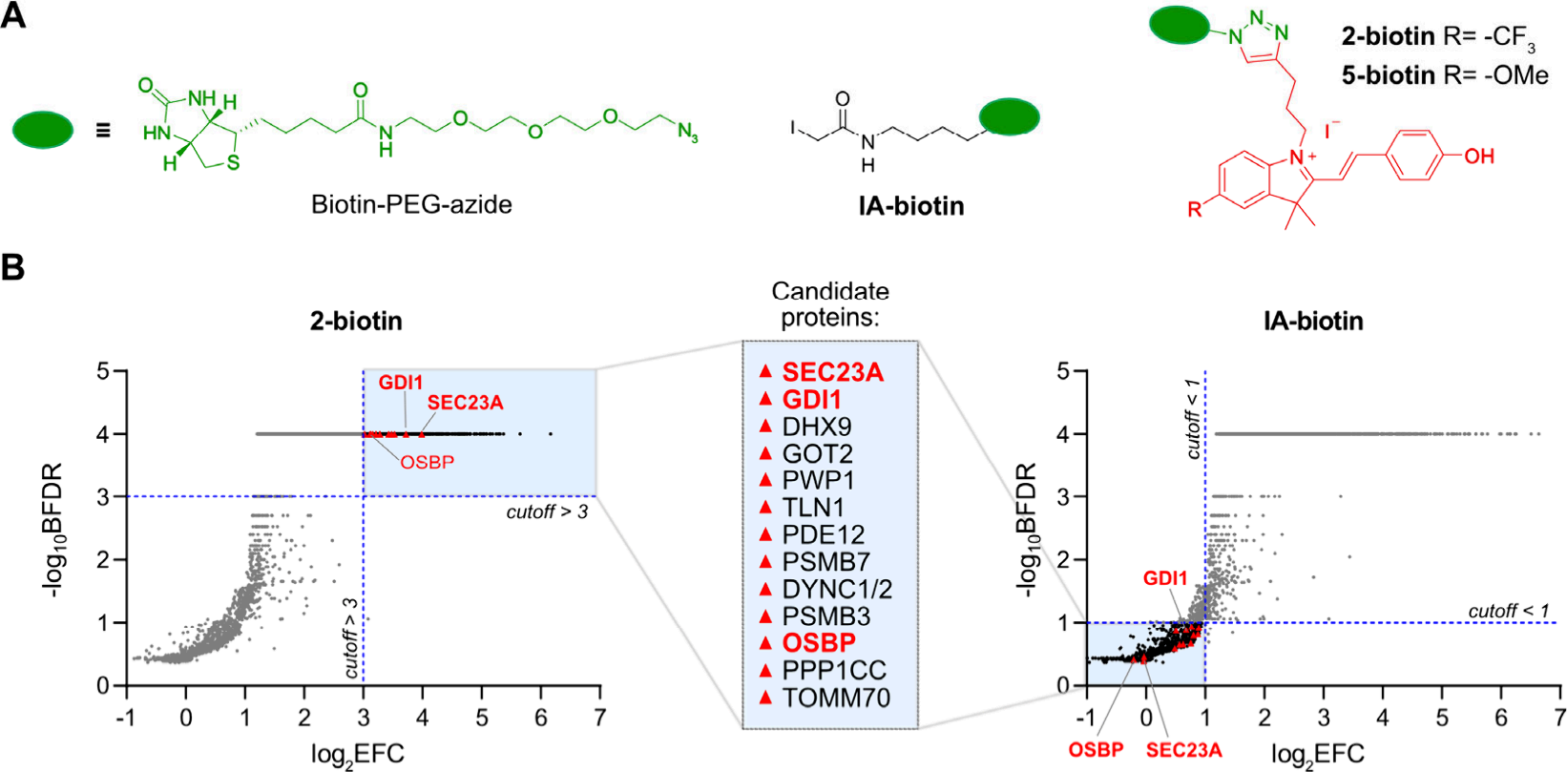
ABPP analysis for protein target identification. (A) Chemical structure of ABPP probes (**biotin-probes**), **biotin-PEG-azide**, and iodoacetamide probes (**IA-biotin**) used in the proteomic experiments. (B) Volcano plots of ABPP experiment with 10 μM of **2-biotin** (*n* = 3) and **IA-biotin** (*n* = 3) in HeLa cell lysate. Significant proteins are highlighted in black and red. Candidate proteins are highlighted as red triangles and labeled in red. The blue line represents the log_2_(enrichment) and −log_10_(BFDR) cutoff used for the selection of the protein candidates: **2-biotin** > 3; and **IA-biotin** < 1. A list of the candidate protein targets found in HeLa cell lysate is inside the light blue box with the two most important candidates highlighted in red.

We treated cell lysates with **2-biotin**, **5-biotin**, or **IA-biotin** for ABPP-based target enrichment. The enriched proteins were subsequently identified through proteomic analysis using liquid chromatography-tandem mass spectrometry (LC-MS/MS) (Figure S13). Proteins captured upon treatment of cell lysates with DMSO were used as a negative control, while **2-biotin, 5-biotin**, and **IA-biotin** were used to identify specific interactors. We employed SAIN*T* express software^49^ to identify significant targets, which we defined as those with a Bayesian false discovery rate (BFDR) threshold of 0.05 and an empirical fold-change score (EFC) threshold of 2 (log_2_(EFC) ≥ 1, Figure S13). Nearly 3000 proteins were enriched using either **2-biotin** or **IA-biotin**. These results suggest that **2-biotin** is a powerful electrophile capable of reacting with many proteins at the concentration used (10 μM). However, **2-biotin** did not react indiscriminately with nucleophilic cysteines, e.g., while protein disulfide isomerases PDI3–6 were efficiently enriched using **IA-biotin**, they were not detected in the fraction enriched by **2-biotin** (Figure S13).

Since **IA** does not induce ER vacuolation and paraptosis, we posited that proteins that react with **2-biotin** but not with **IA-biotin** might be involved in the induction of this phenotype. We set thresholds of log_2_(EFC) and -log_10_(BFDR) of >3 for **2-biotin** and <1 for **IA-biotin** (Figure 7B) and identified 13 proteins that were strongly enriched by **2-biotin** but not by **IA-biotin** (Figure 7B and Table S2). In our ABPP experiment, we treated whole cell lysate with the biotinylated probes, and therefore the whole soluble proteome was exposed to the electrophiles. Live-cell imaging revealed that compound **2** was notably enriched in the endoplasmic reticulum (ER), as indicated by colocalization with ER-specific markers (Figure S4); therefore, from the subset of 13 proteins, we focused only on those known to localize to the ER (Table S2). Based on these considerations, three proteins emerged as potential drivers of ER vacuolation: Sec23 homologue A (SEC23A), GDP-dissociation inhibitor alpha (GDI1), and oxysterol-binding protein 1 (OSBP).

SEC23A is a critical component of COPII-coated vesicles, which transport secretory proteins from the ER to the Golgi apparatus.^50,51^ Interestingly, fibroblasts from individuals carrying mutant SEC23A exhibit ER dilation,^52^ confirming its role in vacuolation. GDI1 is a protein that regulates Rab GTPase activity and plays a key role in vesicular trafficking.^4,53^ Although direct inhibition of GDI1 has not been associated with cytoplasmic vacuolation, its isoform GDI2, which shares similar functions, has been previously linked to paraptosis and ER vacuolation.^4^ Moreover, previous efforts to generate a *GDI1*/*GDI2* double knockout in U2OS cells were unsuccessful, likely due to impaired cell survival and proliferation.^54^ OSBP is a lipid transporter that delivers sterol to the Golgi complex in exchange for phosphatidylinositol 4-phosphate, which is degraded by the SAC1/SACM1L phosphatase in the ER.^55^ OSBP inhibition has been associated with disruption of the Golgi apparatus and impaired retrograde trafficking.^56,57^ Although genetic depletion of OSBP has not been reported to significantly affect cell viability, some small-molecule inhibitors of OSBP exhibit cytotoxic effects, leading to cell death in specific cell lines.^56,57^ Notably, OSBP does not appear to be directly involved in ER vacuolation or paraptosis, as knockdown experiments have failed to induce the characteristic vacuolation.^57^ Based on this precedent, we conclude that hemicyanine **2** induced ER vacuolation and paraptosis primarily through inhibition of SEC23A, with a potential contribution from GDI1.

The proteins discussed above are likely to contribute to paraptosis activation but the cytotoxic effects of compound **2** may involve additional interactions. For instance, mitochondrial proteins aspartate aminotransferase (GOT2)^58^ and mitochondrial import receptor subunit TOMM70 ^59^ may lead to the observed increase in superoxide production, contributing to cell death. Additionally, thioredoxin reductase 1 (TXNRD1) was enriched in both **2-biotin** and **IA-biotin** treatments (Figure S13). TXNRD1 contains a highly reactive selenocysteine residue, making it particularly susceptible to electrophilic compounds.^60,61^ Thus, increasing the selectivity of compound **2** for SEC23A, while decreasing its overall electrophilicity, will be crucial to develop a probe that can be used to study the induction of paraptosis independently from other potentially confounding sources of cytotoxicity.

## Conclusions

Paraptosis serves as an alternative cell death pathway when apoptosis is ineffective, offering a potential therapeutic avenue in diseases where apoptosis is dysregulated. However, the high concentration and prolonged exposure needed to trigger paraptosis highlight the limited potency of current compounds (Table S1). In this study, we introduced hemicyanine compounds as novel paraptosis activators. Among them, compound **2** effectively induces paraptosis in various cell lines by covalently interacting with free cysteine residues in cellular proteins triggering hallmark features of paraptosis, including extensive vacuolation of the ER.

Through ABPP analysis, we identified key protein targets of compound **2**, including SEC23A and GDI1, which are associated with vesicle trafficking and ER vacuolation. Notably, compound **2** displayed some degree of selectivity, with minimal interaction with highly nucleophilic PDIs, underscoring its potential as a chemical probe.

These findings position compound **2** as a promising lead for developing selective, targeted probes to study paraptosis, with potential applications in the development of therapeutics in cancers where traditional apoptotic pathways are impaired. Future studies will focus on optimizing the structure–activity relationships of compound **2** to enhance its potency and selectivity.

## Supplementary Material



## Data Availability

All the raw data from this paper are available on Zenodo at DOI 10.5281/zenodo.15262773. The mass spectrometry proteomics data have been deposited to the ProteomeXchange Consortium via the PRIDE^61^ partner repository with the data set identifier PXD062853.

## Notes

Experimental details, additional results and discussion, ^1^H and ^13^C NMR spectra; HRMS spectra; and LC-MS spectra (PDF)
