# Supplementary material for: Potent Inducers of Paraptosis through Electronic Tuning of Hemicyanine Electrophiles
Source: J Am Chem Soc. 2025 Aug 26;147(36):32571–9. doi: 10.1021/jacs.5c07109 (PMC12426919; doi:10.1021/jacs.5c07109)
Supplement: Supplementary file 1 [file ja5c07109_si_001.pdf]

Supplementary Information

Potent Inducers of Paraptosis Through Electronic Tuning of Hemicyanine Electrophiles

Juan F. Tamez-Fernández,† Craig F. Steven,† Jade Nguyen,‡ Pablo Rivera-Fuentes†*

† Department of Chemistry, University of Zurich, CH-8057, Zurich, Switzerland.

‡ Institute of Chemical Sciences and Engineering, École Polytechnique Fédérale de Lausanne, CH-1015, Lausanne, Switzerland.

Correspondence to: pablo.riverafuentes@uzh.ch

Contents

Compounds inducing paraptosis. 2

Supplementary results. 3

Dose-response curves of cytotoxicity assays. 3

Cytotoxicity assays. 4

pH titration of compounds **2** and **2**-NH_2. 5

Colocalization experiments of compounds **1**-**5** and **2**-NH_2 6

GSH reactivity against compounds **1** and **2**. 7

Apoptosis evaluation experiments. 9

Morphological changes in the ER and mitochondria. 10

Morphological changes in the ER and mitochondria under protein synthesis inhibition 11

$O_2^{\cdot-}$ production measurement in live HeLa cells treated with **2**. 12

Stress-granules induction by compound **2**. 14

Proteomics. 15

Experimental procedures. 18

Synthetic procedures 24

NMR spectra. 32

HRMS spectra. 43

Uncropped gels 47

References. 48

Compounds inducing paraptosis.

Table S1. Potencies and mechanism of chemical compounds that induce paraptosis in mammalian cells.

Compound	Cell line	Concentration and time	Mechanisms involved	Ref.
15-deoxy-Δ12,14-prostaglandin J2 (15d-PGJ2)	MDA-MB-231	20 μM at 9 h	LC3 upregulation, accumulation of ubiquitinated proteins, and disruption of sulfhydryl homeostasis.	[1]
2'-hydroxy-retrochalcone	MDA-MB-231	30 μM at 24 h	Proteasomal inhibition, and ER stress	[2]
6-Shogaol	MDA-MB-231	40 μM at 12 h.	Proteasomal inhibition, ER stress (upregulation of CHOP, BIP)	[3]
Celastrol	MDA-MB-435S	2 μM at 8 h	Proteasomal inhibition, Mitochondrial Ca^{2+} overload, and ER stress.	[4]
Curcumin	MDA-MB-231	40 μM at 8-24 h	Proteasomal dysfunction, mitochondrial Ca^{2+} overload, and superoxide production.	[5,6]
Curcuminoid B63	SGC-7901	20 μM at 12 h	Inhibition of TrxR1, increase of ROS levels, and ER stress.	[7]
Dimethoxycurcumin	MDA-MB-231	20 μM at 8 h	Proteasomal inhibition, ER stress, and accumulation of ubiquitinated proteins.	[5]
Elaiophylin	SKOV3, OVCAR8	0.5 μM at 6 h	MAPK hyperactivation, and ER stress. Target SHP2 (encoded by PTPN11).	[8]
Gambogic acid	MDA-MB-435S	1 μM at 8-24 h	Reaction with thiol-containing proteins, swollen mitochondria, proteasomal inhibition, and ER stress.	[9]
Jolkinolide B	T24, UM-UC-3	10 μM at 12 h	Inhibition of thioredoxin reductase 1 (TrxR1), depletion of glutathione (GSH), production of ROS, and ER stress.	[10]
Manumycin A	MDA-MB-231	1-5 μM at 9 h	Expression of LC3 and p62, ER stress Accumulation of ubiquitinated proteins.	[11]
Ophiobolin A	U373-MG, T98G, and GL19.	1 μM at 72 h	Inhibition of the BKCa channel activity.	[12]
Tunicamycin	FRO cells	12 μM at 48 h	Paraptosis is relevant to de novo protein synthesis and proteasomal activity. Inhibition of $BRAF^{V600E}$ potentiates TM-induced paraptosis.	[13]
Withaferin A	MDA-MB-231	4 μM at 24 h	ROS production, and ER stress	[14]

Supplementary results.

Dose-response curves of cytotoxicity assays.

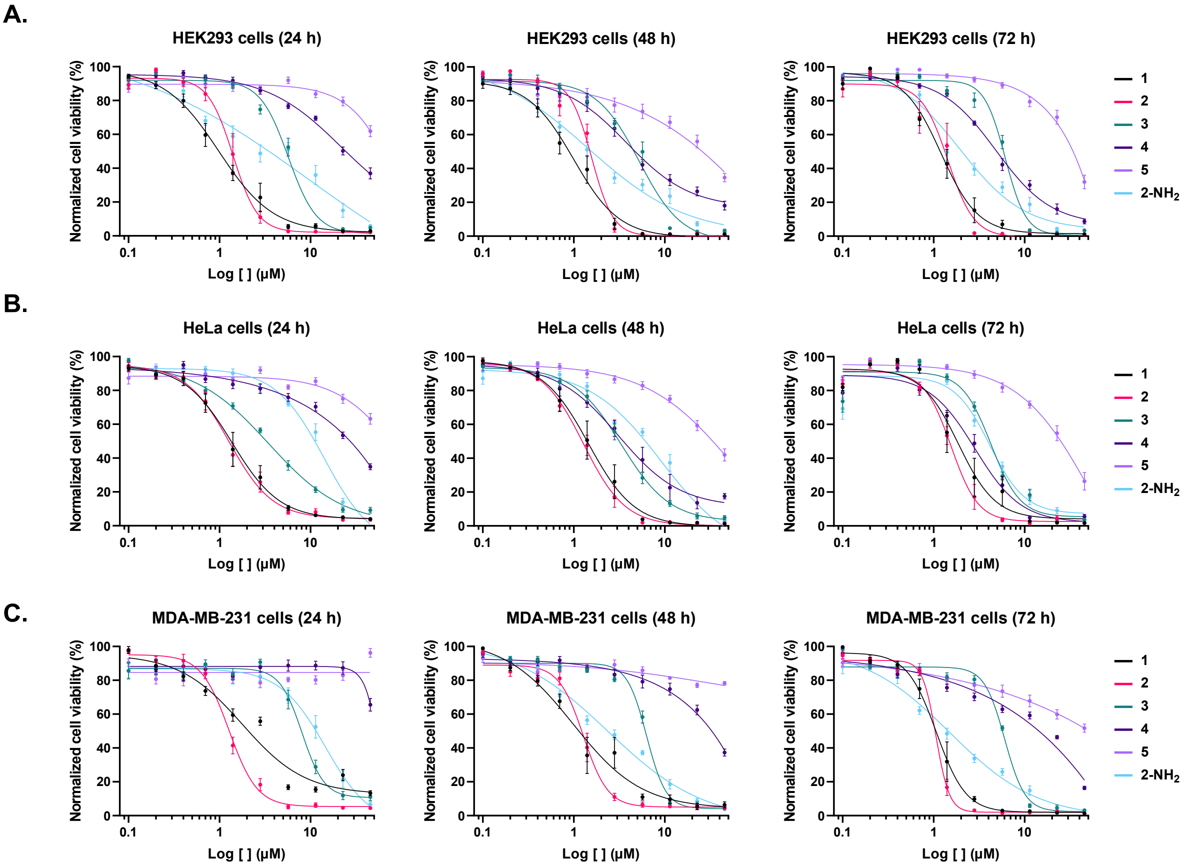

Figure S1. Dose-response curves of cytotoxicity assays of compounds 1-5 and 2-NH$_2$. Dose-response curves of cytotoxicity assays of compounds **1-5** and **2-NH$_2$** in HEK293 **(A.)**, HeLa **(B.)**, and MDA-MB-231 cells **(C.)**. Cells were incubated with compounds at concentrations from 44 nM to 45 µM. Cell viability was assessed by MTT assay at 24 h, 48 h, and 72 h. IC_{50} was calculated by taking as 100% of viability the DMSO-treated cells. The experiments were performed in triplicate for each compound. Means are plotted and error bars represent standard mean error.

Cytotoxicity assays.

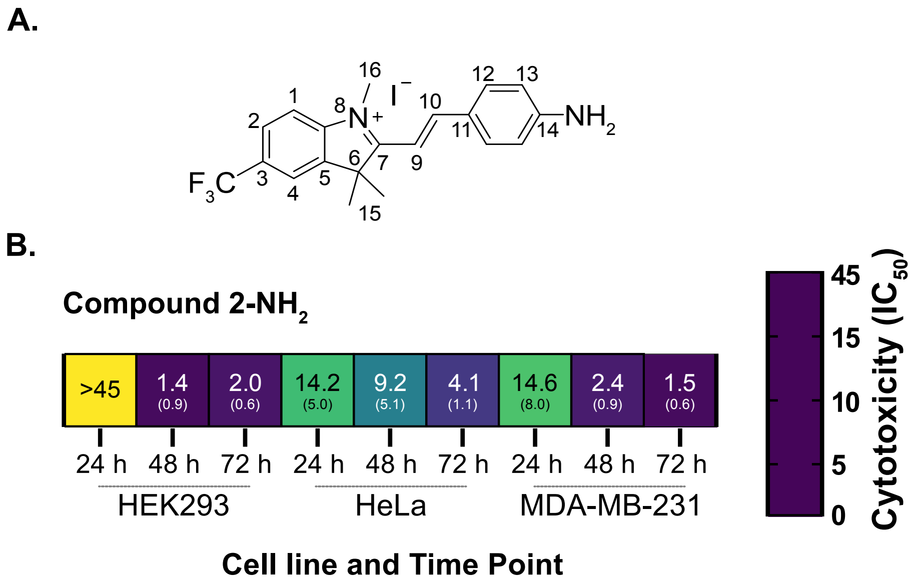

Figure S2. Cytotoxicity of hemicyanine 2-NH$_2$. A. Chemical structure of compound **2-NH$_2$; B.** Heat map showing IC$_{50}$ values of cytotoxicity assays of compounds **2-NH$_2$** in HEK293, HeLa, and MDA-MB-231 cells. Cells were incubated with compound at concentrations from 44 nM to 45 µM. Cell viability was assessed by MTT assay at 24 h, 48 h, and 72 h. IC$_{50}$ was calculated by taking as 100% of viability the DMSO-treated cells. The experiments were performed in triplicate for each compound. Means and standard deviation (in parenthesis) are shown in each panel.

*pH titration of compounds **2** and **2-NH$_2$**.*

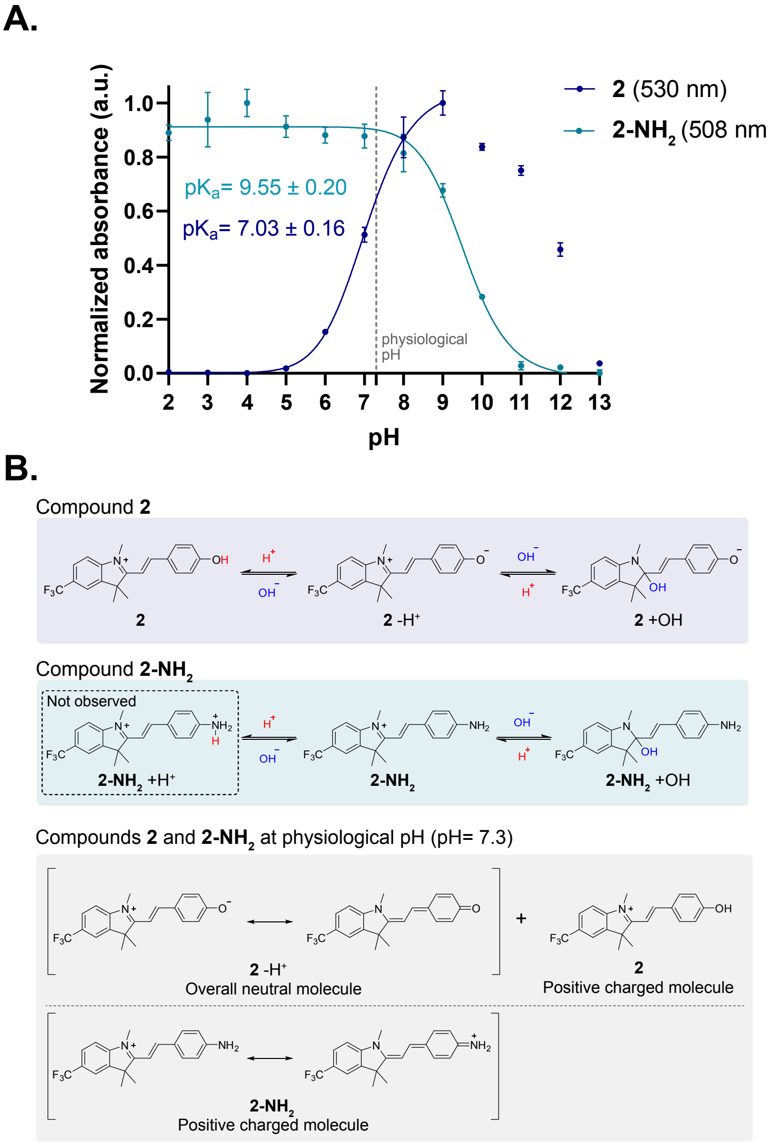

Figure S3. Determination of pK_a of compounds 2 and 2-NH$_2$. A. Absorbance vs pH plot at 530 nm (compound **2**) and 508 nm (compound **2-NH$_2$**) for pK_a measurement. Compounds were prepared at 20 μM concentrations in buffers with a pH range of 2 to 13. pK_a values are represented for each compound as mean ± standard deviation with the corresponding color. Means are plotted and error bars represent standard error. Measurements were carried out for triplicates. The Gray dotted line represents physiological pH. For compound **2**, the curve was fitted up to pH 9, as absorbance begins to decrease from pH 10 due to hydroxyl attack of the molecule; **B.** Schematic representation of the structures of compounds **2** and **2-NH$_2$** when exposed to acid, base, and physiological pH. Colors represent the behavior observed in panel (A). At physiological pH, compound **2** exists in two forms: a positively charged form and a neutral form, while compound **2-NH$_2$** exists only as a positively charged form.

*Colocalization experiments of compounds **1-5** and **2-NH$_2$***

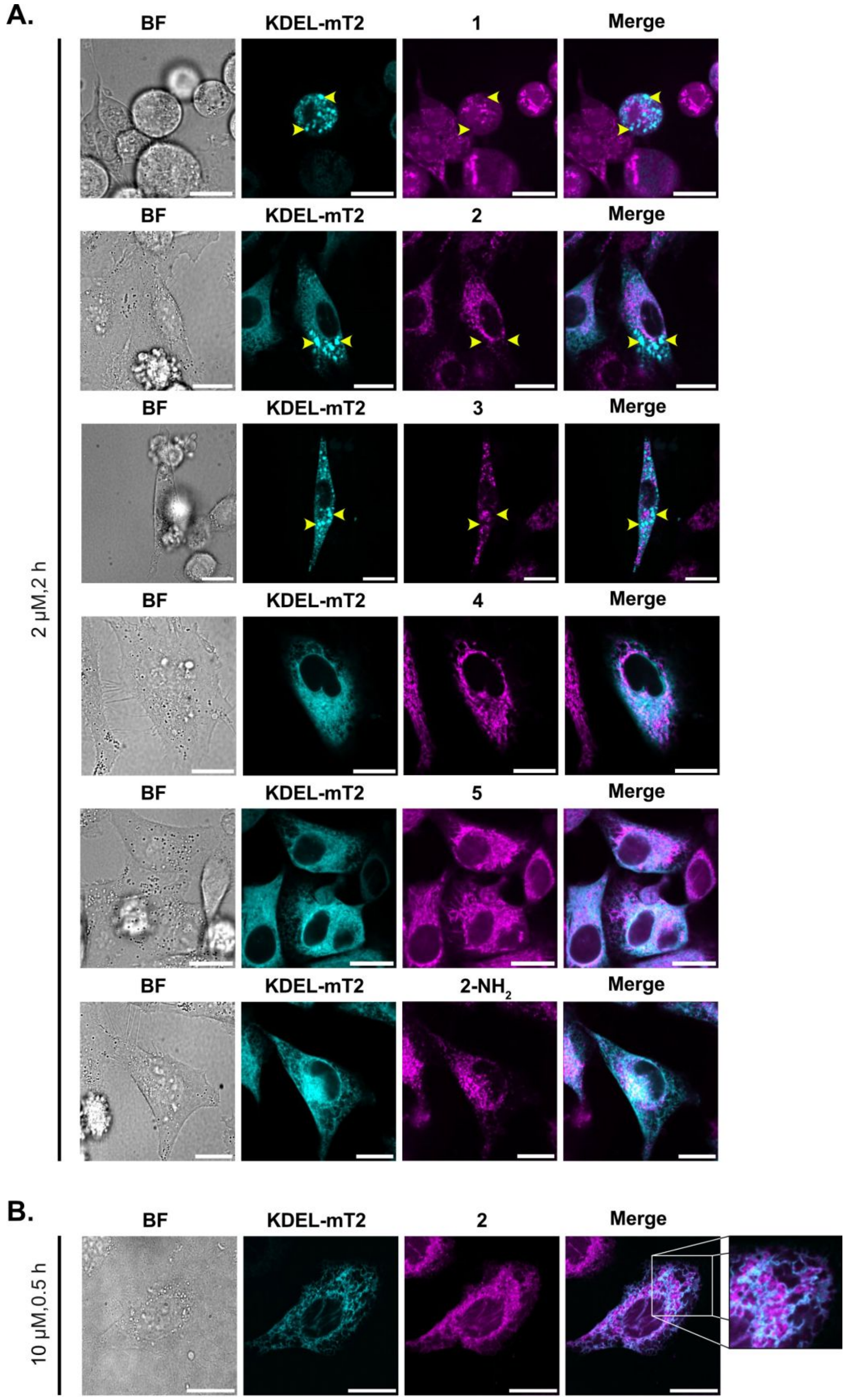

Figure S4. Colocalization and morphological changes of compounds 1-5 and 2-NH$_2$ in live HeLa cells. **A.** Colocalization and vacuolation of compounds **1-5** and **2-NH$_2$.** Cells were transfected with KDEL-mTurquoise2 two days before imaging and

incubated with compounds **1-5** and **2-NH$_2$** (2 μM, 2 h). In each panel from left to right: brightfield, fluorescence of KDEL-mTurquoise2 (laser: 445 nm, 400 ms, 1.5 mw), fluorescence of compounds **1-5,** and **2-NH$_2$** (laser: 561 nm, 300 ms, 3.5 mW), and merged images. The overlapping signals are shown in white; the ER-vacuoles are pointed by the yellow arrows. Scale bars = 20 μm. **BF**= brightfield, **KDEL-mT2**= KDEL-mTurquoise2. For compound **2-NH$_2$**, ER-vacuoles were observed at concentrations higher than 10 μM (data not shown). **B.** Accumulation of compound **2** to the ER and other compartments. Cells were transfected with KDEL-mTurquoise2 two days before imaging, and incubated with compound **2** (10 μM, 0.5 h). In each panel from left to right: brightfield, fluorescence of KDEL-mTurquoise2 (laser: 445 nm, 100 ms, 1.5 mW), fluorescence of compounds **2** (laser: 561 nm, 300 ms, 3.5 mW), and merged images (with a zoom-in region). The overlapping signals are shown in white. Scale bars = 20 μm. **BF**= brightfield, **KDEL-mT2**= KDEL-mTurquoise2.

*GSH reactivity against compounds **1** and **2**.*

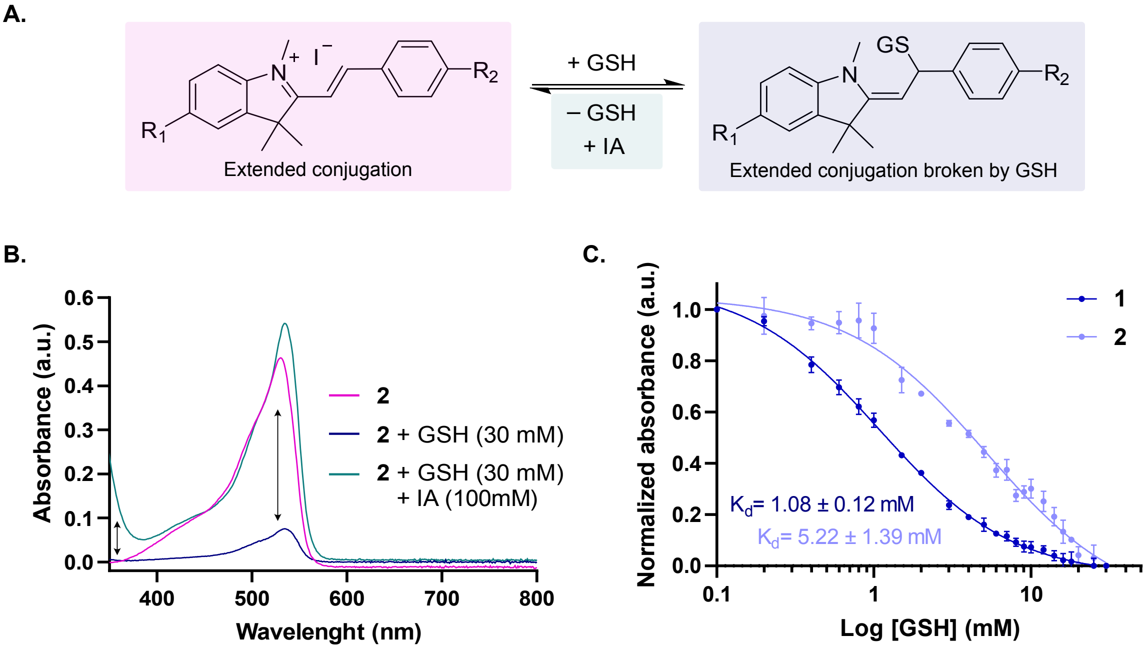

Figure S5. Reactivity of compounds 1 and 2 against reduced glutathione (GSH). A. Scheme illustrating the reversible reaction between hemicyanine electrophiles and reduced glutathione (GSH). Colors represent the behavior observed in panel (B). **B.** Absorption spectra of **2**, **2** (10 μM) incubated with GSH (30 mM), and **2** (10 μM) incubated with GSH (30 mM) and iodoacetamide (100 mM), in 0.5 M sodium

phosphate buffer. The absorbance was measured at 25 ºC. The reversibility of the reaction was evaluated by adding an excess of iodoacetamide (100 mM) to the reaction mixture containing **2** and **GSH**. This addition was intended to trap the GSH molecules and prevent any further reaction with compound **2**. The arrows show the change in absorbance, explained by the disruption of extended conjugation caused by GSH as seen in panel (A); **C.** Normalized absorbance vs log of GSH concentration plot for $K_{d,\ GSH}$ measurement of compound **1** (at 541 nm) and **2** (at 531 nm)**.** Means are plotted and error bars represent standard error. Measurements were carried out for triplicates. K_d values are represented for each compound as mean ± standard deviation with the corresponding color.

Discussion of Figure S5.

Considering the electrophilic nature of the double bond (C9-C10, Fig. 1A) in compound **2**, we assessed its reactivity with prevalent thiol groups in cells, such as reduced glutathione (GSH). First, we used UV-Vis spectroscopy to investigate the reactivity of compound **2** with GSH. We reacted compound **2** (10 µM) with different concentrations of GSH within the biological range (0-30 mM). We observed that compound **2** exhibited a maximum absorption at 531 nm, and the absorbance at this wavelength decreased as the concentration of GSH increased, accompanied by an increase at around 300 nm (Fig. S5B). These spectral shifts indicate that GSH was incorporated into the double bond of compound **2**.[15]

To assess the reversibility of this reaction, we introduced an excess of iodoacetamide (100 mM) to quench the dissociated GSH. The recovery of absorbance at 531 nm confirmed the dissociation of GSH and the restoration of the extended conjugation in compound **2** (Fig. S5B). Furthermore, we determined the GSH dissociation constant ($K_{d,GSH}$) for compounds **1** and **2**. (Fig. S5C).

Apoptosis evaluation experiments.

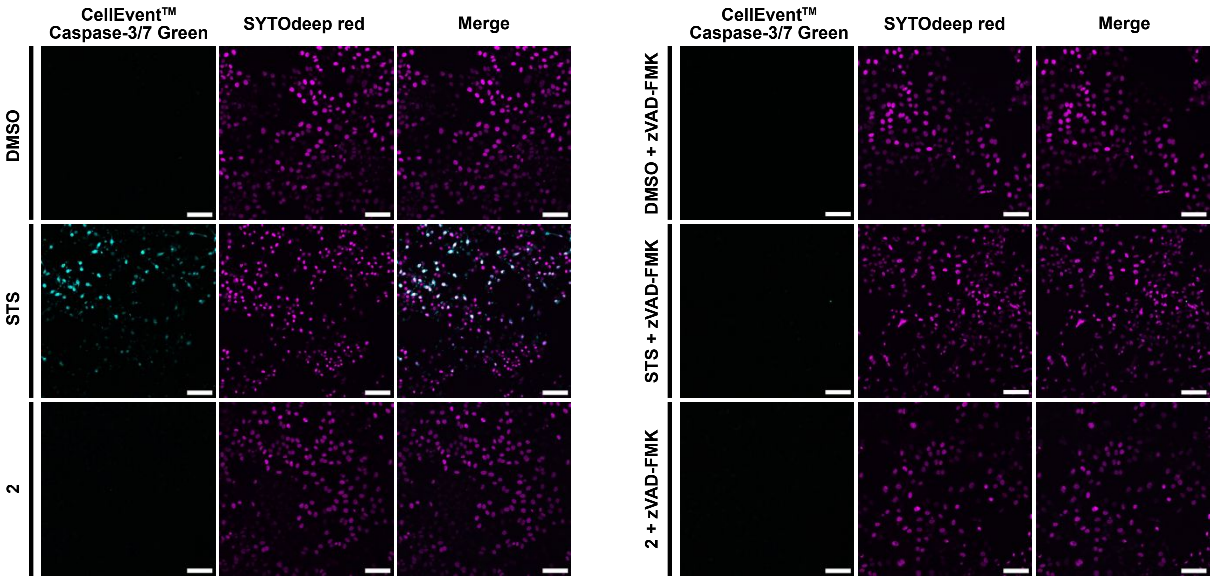

Figure S6. Apoptosis evaluation in live HeLa cells treated with 2. Quantification of apoptotic cells treated with Staurosporine (STS) or **2**, with and without pan-caspase inhibitor (zVAD-FMK), compared to DMSO control. Cells were incubated with DMSO (0.1%, 1h), Staurosporine (2 µM, 4 h), and compound **2** (2 µM, 2 h). When indicated the cells were pre-incubated with zVAD-FMK (20 µM, 2 h), then co-incubated with DMSO, STS, and **2** as mentioned above. Cells were stained with SYTO™ Deep Red nucleic acid stain (1X, 30 min, 37 °C) and CellEvent™ Caspase-3/7 green reagent (3 µM, 30 min, 37 °C) before imaging. In each panel from left to right: fluorescence of CellEvent™ Caspase-3/7 green (laser: 488 nm, 300 ms, 0.8 mW), fluorescence of SYTO™ Deep Red nucleic acid stain (laser: laser: 640 nm, 100 ms, 4.3 mW), and merged images. The overlapping signals are shown in white. Scale bars = 100 µm.

Morphological changes in the ER and mitochondria.

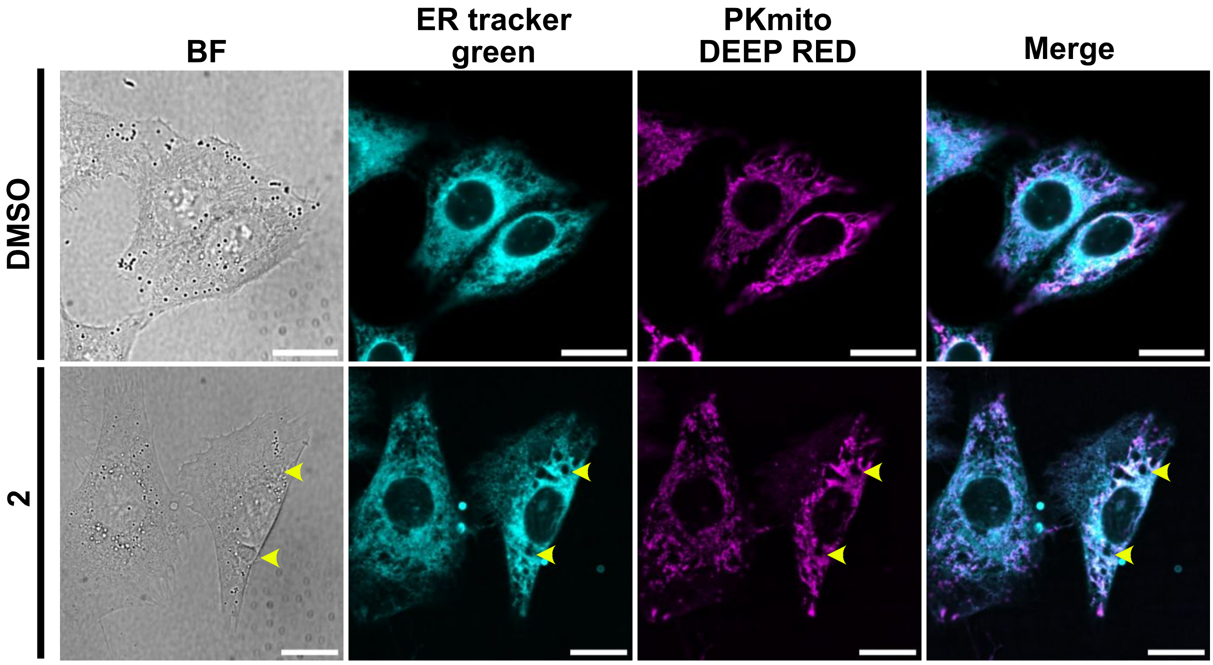

Figure S7. Involvement of mitochondria in vacuole formation in HeLa cells. Cells were incubated with ER Tracker™ Green (1 µM, 1 h) and PKmito™ DEEP RED (1X, 1 h), and incubated with DMSO (0.1%, 2 h) or **2** (2 µM, 2 h). In each panel from left to right: fluorescence of ER Tracker™ Green (laser: 488 nm, 300 ms, 0.7 mW), fluorescence of PKmito™ DEEP RED (laser: 640 nm, 100 ms, 3.7 mW), merged images. The overlapping signals are shown in white; the ER-vacuoles are pointed by the yellow arrows. Scale bars = 20 µm.

Morphological changes in the ER and mitochondria under protein synthesis inhibition

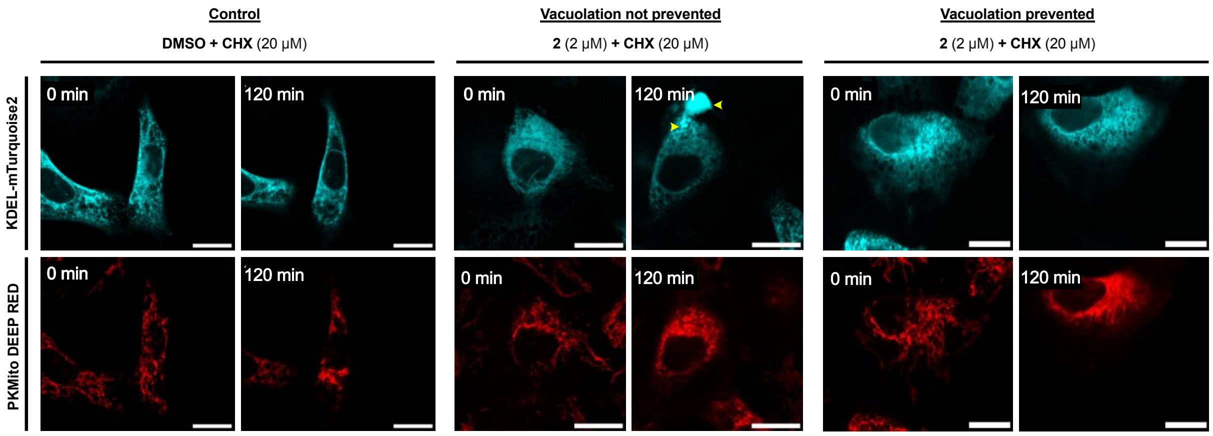

Figure S8. Evaluation of the relationship between mitochondrial damage and vacuole formation. Cells were transfected with KDEL-mTurquoise2 two days before imaging, stained PKmito^{TM} DEEP RED (1X, 1 h), and preincubated with CHX (20 μM, 2 h), then co-incubated with DMSO (0.1%, 2 h), or **2** (2 μM, 2 h). In each panel from left to right: fluorescence of KDEL-mTurquoise2 (laser: 445 nm, 400 ms, 1.5 mW) or fluorescence of PKmito^{TM} DEEP RED (laser: 640 nm, 100 ms, 3.7 mW) at 0 min and 120 min. The ER-vacuoles are pointed by the yellow arrows. Damaged mitochondria are observed at 120 min in PKmito^{TM} DEEP RED fluorescence panel with compound **2** treatment. Scale bars = 20 μm.

*$O_2^{\bullet-}$ production measurement in live HeLa cells treated with **2.***

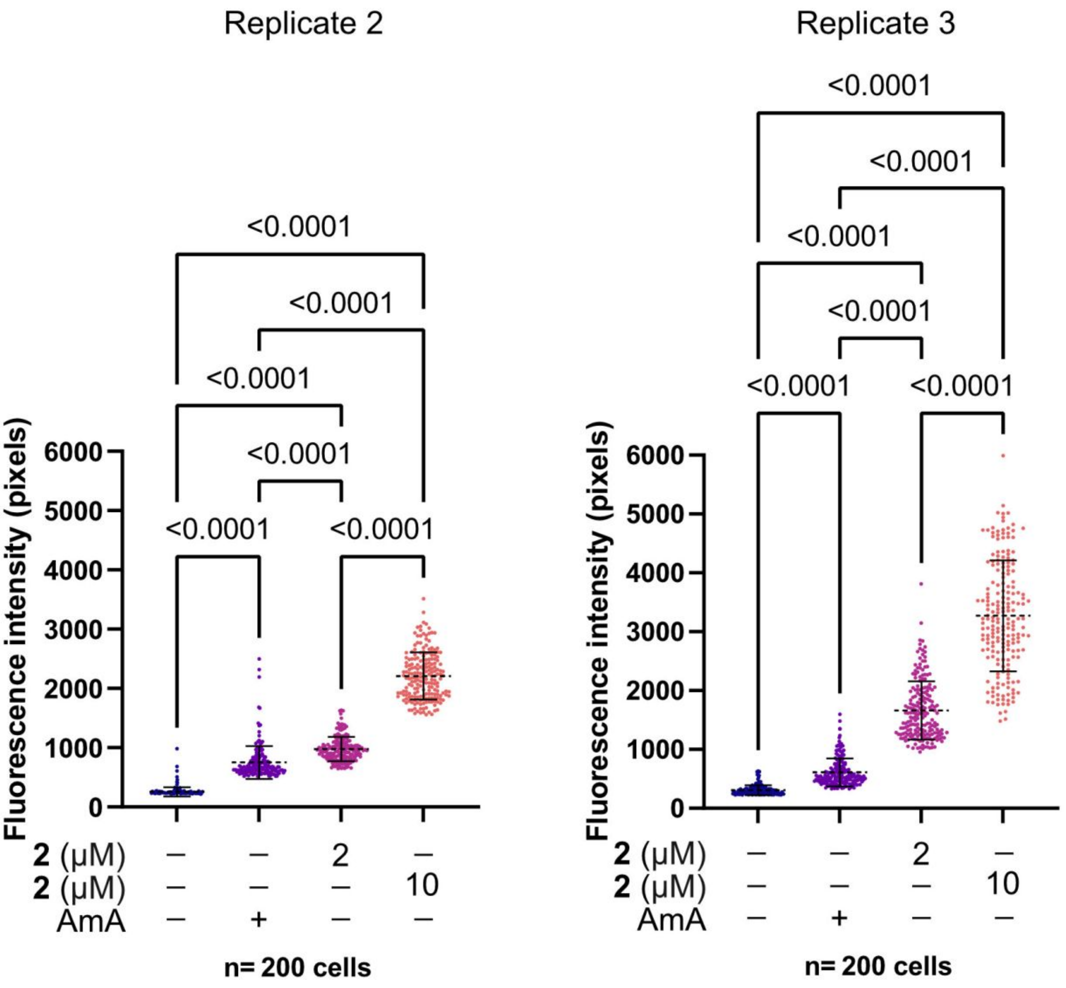

Figure S9. $O_2^{\bullet-}$ production measurement in live HeLa cells treated with 2. The cells were incubated with HKSOX-1* (10 μM, 30 min) before imaging and incubated with DMSO (0.1%, 1h), compound **2** (2 and 10 μM, 1 h), and AmA (10 μM, 1 h). Then, the fluorescence intensity was quantified and compared to the DMSO control of the cells treated as described above. Means are plotted, and error bars represent the standard deviation. Measurements were carried out for 200 cells across biological triplicates, and two replicates were plotted. *P*-values are indicated for each treatment comparison and were calculated using a one-way ANOVA test with a Tukey comparison.

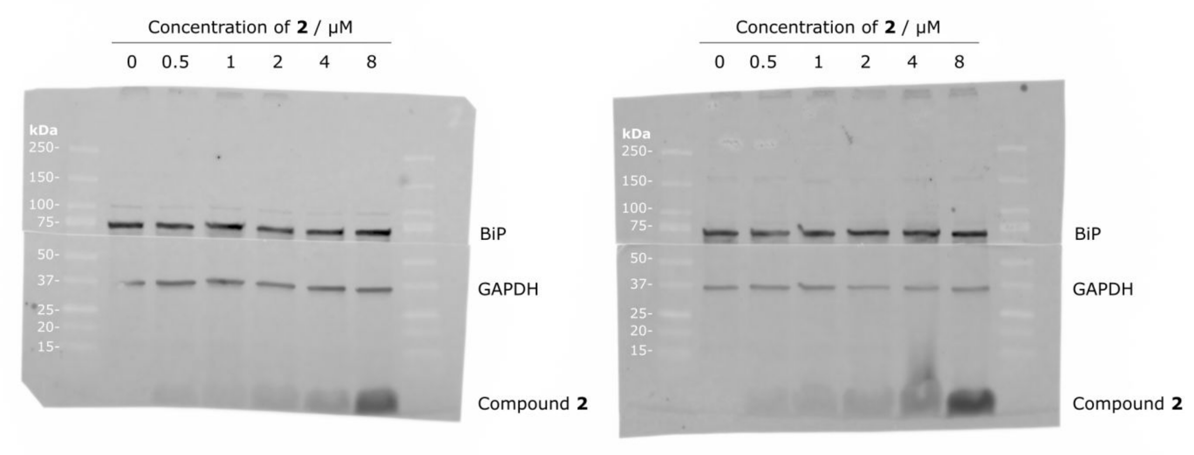

Figure S10. Uncropped Western blots of HeLa cell lysates after 2 h incubation with **2** at the indicated concentration. BiP/GRP78 expression was detected using an anti-GRP78 primary antibody (Thermo Fisher Scientific, #MA527686) with an anti-GAPDH loading control (Cell Signalling Technology, #5174). Background fluorescence of **2** is visible in the lower portion of each running lane. Two biological replicates (left and right) are shown.

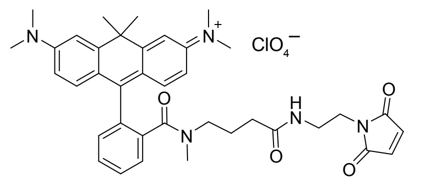

Figure S11. Chemical structure of ATTO 620 maleimide probe. The commercially available probe was used in the gel-based, qualitative activity-based protein profiling (ABPP).

*Stress-granules induction by compound **2.***

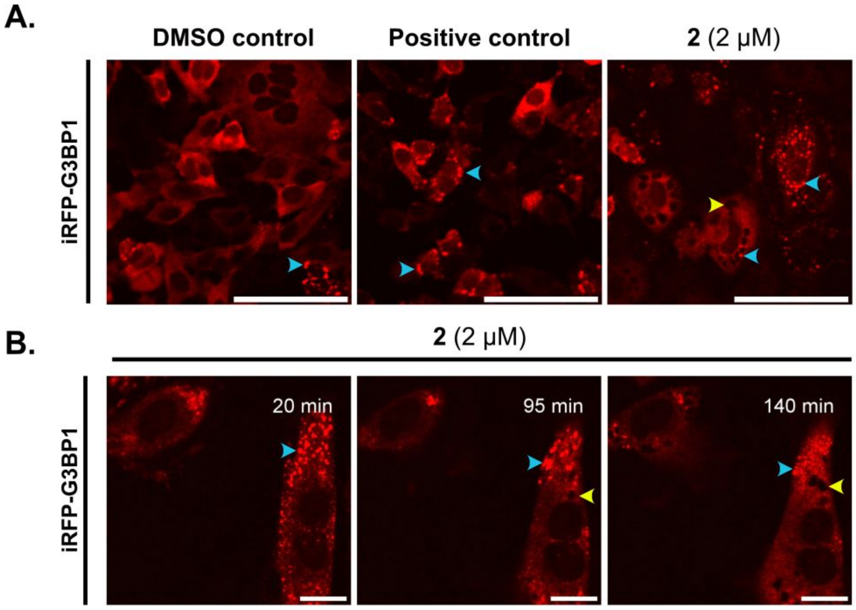

Figure S12 Stress-granules induction by compound 2. A. Induction of stress granules formation by **2** in HeLa cells. Cells were transfected with iRFP-G3BP1 plasmid two days before imaging, and incubated with DMSO (0.1%, 2.5 h) or **2** (2 μM, 2.5 h). DMSO was used as a negative control (some cells showed stress granules due to intrinsic cell stress). DMSO control was heated at 43 °C for 0.5 h and was used as a positive control. Each panel shows the fluorescence of iRFP-G3BP1 (laser: 640 nm, 100 ms, 3.7 mW). The ER vacuoles are pointed by the yellow arrows and the stress granules by the light blue arrows. Scale bars = 100 μm; **B.** Time-lapse of stress-granules formation by **2** in HeLa cells. Cells were transfected with iRFP-G3BP1 plasmid two days before imaging, and incubated with or **2** (2 μM, 2.5 h). In each panel from left to right: fluorescence of iRFP-G3BP1 (laser: 640 nm, 100 ms, 3.7 mW) at 20, 95 and 140 min. The ER vacuoles are pointed by the yellow arrows and the stress granules by the light blue arrows. Scale bars = 20 μm.

Proteomics.

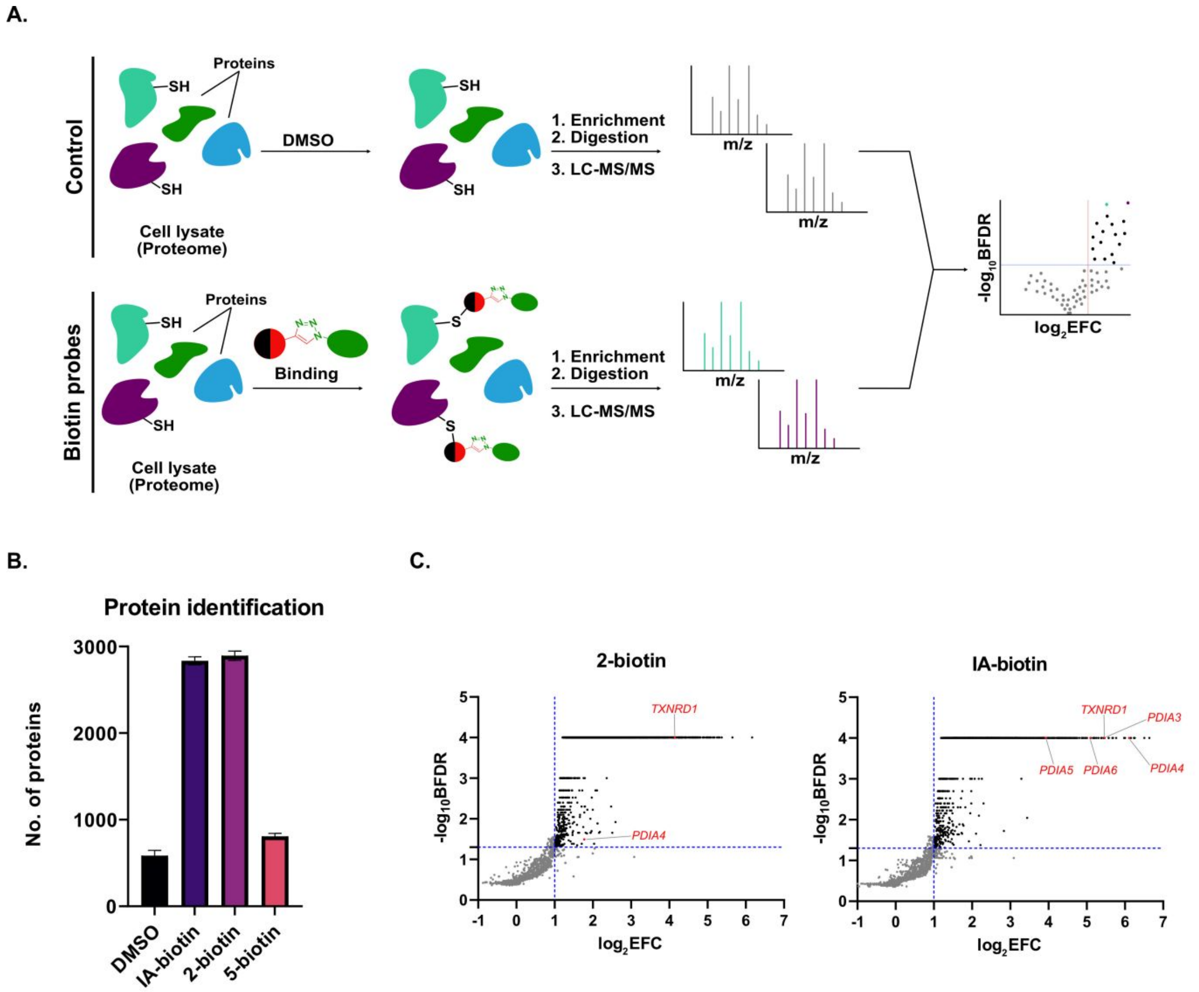

Figure S13. ABPP analysis for protein target identification. HeLa cell lysates were treated with DMSO (0.1%, 2 h) or **2-biotin**/**5-biotin**/**IA-biotin** (10 μM, 2 h). **A.** Schematic overview of ABPP approach with **2-biotin, 5-biotin,** and **IA-biotin** in HeLa cells lysate with MS/MS-based read-out. **B.** Number of proteins identified and quantified per treatment. Means are plotted and error bars represent standard deviation. Measurements were carried out by biological triplicates. **C.** Volcano plots of ABPP experiment with 10 μM of **2-biotin** (n = 3) and **IA-biotin** (n = 3) in HeLa cell lysate using DMSO (n = 3) as a negative control. Significant proteins are highlighted in black and red, and some important proteins are labeled red. The blue line represents the Bayesian false discovery rate (BFDR) threshold of 0.05 and an empirical fold-change score (EFC) threshold of 2 ($\log_2$(EFC) ≥ 1) obtained by the analysis with SAINTexpress software.

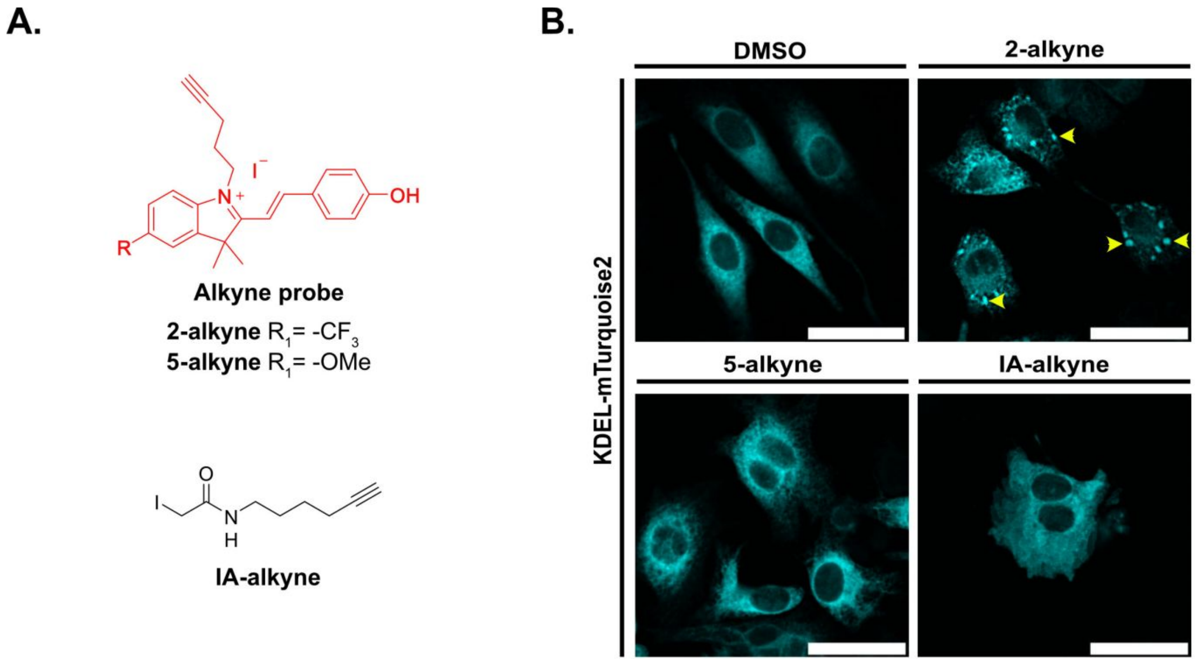

Figure S14. Induction of cytoplasmic vacuolation by 2-alkyne, 5-alkyne, and IA-alkyne in HeLa cells. A. Chemical structures of compounds **2-, 5-, and IA-alkyne. B.** Assessment of cytoplasmic vacuolation in HeLa cells treated with **2-**, **5-**, and **IA-alkynes**. Cells were transfected with KDEL-mTurquoise2 two days before imaging, and incubated with DMSO (0.1%, 2 h), **2-alkyne/5-alkyne** (2 μM, 2 h), or **IA-alkyne** (10 μM, 2 h). Each panel shows the fluorescence of KDEL-mTurquoise2 (laser: 445 nm, 400 ms, 1.9 mW). The ER-vacuoles are pointed by the yellow arrows. Scale bars = 50 μm.

Table S2. Significant top 13 protein candidates for **2-biotin**, from the interaction data between **2-biotin** and **IA-biotin** probes.

Ranking	Gene	Description	Subcellular localization[a]	Mol. weight (Da)[a]	N° of cysteines[a]	log_2EFC	-log_{10}BFDR
**1**	***SEC23A***	**Protein transport protein Sec23A**	**ER/Cytosol**	**86'161**	**16**	**3.990683**	**4**
**2**	***GDI1***	**Rab GDP dissociation inhibitor alpha**	**Golgi/Cytosol**	**50'583**	**11**	**3.724105**	**4**
**3**	*DHX9*	ATP-dependent RNA helicase A	Nucleus	140'958	23	3.528321	4
**4**	*GOT2*	Aspartate aminotransferase, mitochondrial	Mitochondria	47'518	7	3.477936	4
**5**	*PWP1*	Periodic tryptophan protein 1 homolog	Nucleus	55'828	10	3.429482	4
**6**	*TLN1*	Talin-1	Cytosol/cytoskeleton	269'767	38	3.282736	4
**7**	*PDE12*	2',5'-phosphodiesterase 12	Mitochondria	67'352	16	3.275603	4
**8**	*PSMB7*	Proteasome subunit beta type-7	Cytosol/nucleus	29'965	6	3.214902	4
**9**	*DYNC1/2*	Cytoplasmic dynein 1 intermediate chain 2	Cytosol/cytoskeleton	71'457	7	3.213347	4
**10**	*PSMB3*	Proteasome subunit beta type-3	Cytosol/nucleus	22'949	5	3.16366	4
**11**	***OSBP***	**Oxysterol-binding protein 1**	**ER/Golgi/Cytosol**	**89'421**	**15**	**3.131589**	**4**
**12**	*PPP1CC*	Serine/threonine-protein phosphatase PP1-gamma catalytic subunit	Nucleus/mitochondria/cytosol	36'984	13	3.114367	4
**13**	*TOMM70*	Mitochondrial import receptor subunit TOM70	Mitochondria	67'455	12	3.022545	4

[a]Information obtained from the UniProt Knowledgebase (https://www.uniprot.org/).

Experimental procedures.

General methods.

All reagents and solvents were purchased from commercial sources and used as received. NMR spectra were acquired on a Bruker 500 MHz instrument. ^1H NMR chemical shifts are reported in ppm relative to $SiMe_4$ (δ = 0) and were referenced internally with respect to residual protons in the solvent (δ = 7.26 for $CDCl_3$, δ = 2.50 for $(CD_3)_2SO$, and δ = 3.31 for CD_3OD). Coupling constants are reported in Hz. Splitting patterns are designated as singlet (s), broad singlet (br. s), doublet (d), triplet (t), and multiplet (m). Splitting patterns that could not be interpreted or easily visualized are designated as multiplet (m). ^{13}C NMR chemical shifts are reported in ppm relative to $SiMe_4$ (δ = 0) and were referenced internally to solvent signal (δ = 77.16 for $CDCl_3$, δ = 39.52 for $(CD_3)_2SO$, and δ = 49.00 for CD_3OD. Preliminary peak assignments are based on calculated chemical shifts and multiplicity. LC-MS measurements were performed using an Agilent 1290 Infinity II LC with an Agilent InfinityLab LC/MSD. Columns: C18 (Waters, gradient: 5.5 min, 10 to 95% acetonitrile in water with 0.1% formic acid and 0.04% trifluoroacetic acid). High-Resolution Mass Spectra (HRMS) were measured by the staff of the Mass Spectrometry Laboratory of the Department of Chemistry at the University of Zurich employing a timsTOF Pro TIMS-QTOF-MS instrument (Bruker Daltonics GmbH, Bremen, Germany) by using electrospray ionization (ESI) mode at 4'000 V (-4'000 V) capillary voltage and -500 V (500 V) endplate offset with a N_2 nebulizer pressure of 0.4 bar and a dry gas flow of 4 L min^{-1} at 180°C. IUPAC names of all compounds are provided and were determined using CS ChemDraw Professional 21.0.

Optical spectroscopic methods.

Stock solutions were prepared in DMSO (spectrophotometric grade >99.9%) at concentrations of 10 mM and 2 mM and stored at –20 °C.

*Reactivity of compounds **1** and **2** against reduced glutathione (GSH)*: Compounds **1** and **2** were incubated with different concentrations of GSH (0-30 mM). Spectroscopic measurements were conducted in a 0.5 M sodium phosphate buffer. The UV-visible spectra were acquired employing a Multiskan SkyHigh Microplate Spectrophotometer (ThermoFisher Scienntific). All measurements were conducted at ambient

temperature and using quartz cuvettes from Starna (10 mm path length). The UV-visible spectra were background-corrected, and the absorbance maxima of each GSH concentration value were normalized. Normalized absorbance values were plotted against the log_{10} of GSH concentration value and fitted (non-linear fit, sigmoidal, 4PL) using Prism 9.

*pH titration of compounds **2** and **2-NH$_2$**:* Buffered aqueous solutions in the pH range of 2 to 8 were prepared by mixing citric acid (0.1 M) and sodium dihydrogen phosphate NaH_2PO_4 (0.2 M) in ultrapure water. Buffered aqueous solutions in the pH range of 9 to 11 were prepared by mixing sodium bicarbonate (0.1 M) and sodium carbonate (0.1 M) in ultrapure water. Buffered aqueous solutions in the pH range of 12 to 13 were prepared by mixing potassium chloride KCl (0.2 M) and sodium hydroxide (0.2 M); 20 µM solutions of compounds **2** and ***2-NH$_2$*** in the buffered aqueous solutions were prepared and the absorbance spectra were recorded in triplicates using 96-well plates (Corning) on a Multiskan SkyHigh Microplate Spectrophotometer (ThermoFisher Scienntific). The UV-visible spectra were background-corrected, and the absorbance maxima of each pH value were normalized. Normalized absorbance values were plotted against the pH value and fitted (non-linear fit, sigmoidal, 4PL) using Prism 9.

Mammalian cell cultures

HEK293, HeLa, and MDA-MB-231 cells were grown in DMEM medium supplemented with fetal bovine serum (FBS, 10%) and antibiotics (1%) at 37 °C in 5% CO_2 environment. The cells were passaged at 80–90% confluence. For microscopy experiments, 9´000-15’000 cells per well were seeded into 8-well plates (Ibidi chambered cover glass) or 96-well plates 1–3 days before imaging. The probes were incubated in growth medium for the indicated time. Before imaging, the growth medium was removed, and the cells were washed with PBS and imaged in FluoroBrite DMEM.

Microscopy experiments.

HEK293, HeLa, and MDA-MB-231 cells were transfected 24–48 h before imaging using KDEL-mTurquoise2 (#36204) plasmid obtained from Addgene. Cells were transfected using the jetPRIME® kit, according to the manufacturer's instructions (Polyplus), and the medium was replaced with a fresh growth medium after 4–5 h. Cells were incubated with compounds at the indicated concentrations and times in DMEM. The medium was removed, the cells were washed with PBS and then imaged

in FluoroBrite DMEM. When needed, the FluoroBrite DMEM contained an organelle marker such as ER Tracker^{TM} green (Invitrogen™) or PKmito^{TM} DEEP RED (Spirochrome) at the indicated concentrations. Fluorescent emission of the compounds (laser: 561 nm, 300 ms, 3.5 mW), KDEL-mTurquoise2 (laser: 445 nm, 400 ms, 1.5 or 1.9 mW), ER Tracker^{TM} Green (laser: 488 nm, 300 ms, 0.7 mW), PKmito^{TM} DEEP RED (laser: 640 nm, 100 ms, 3.7 mW) were recorded in a Nikon CSU-W1 single camera confocal microscope.

Cytotoxicity assay

Cell viability was determined according to the MTT method in HEK293, HeLa, and MDA-MB-231 cells. The cells were seeded into 96 well plates (9’000 cells in 90 µL of medium per well) 24 h prior to incubation with compounds. The cells were incubated with compounds at concentrations from 44 nM to 45 µM. After 24 h of incubation, cell viability was assessed by adding 10 µL of a 0.5% (w/v) solution of 3-(4,5-dimethylthiazol-2-yl)-2,5-diphenyl tetrazolium bromide (MTT). IC_{50} was determined as the concentration of the compound required to reduce cell viability by 50%, taking as 100% of viability the DMSO-treated cells. The experiments were performed in triplicate for each compound.

Quantification of apoptotic cells

HeLa cells were incubated for 2 h with the inhibitor (zVAD-FMK, 20 µM) followed by co-incubation with compound **2** (2 µM, 2 h) or Staurosporine (2 µM, 4 h) in DMEM. DMSO (0.1%, 4 h) was used as a negative control and Staurosporine (2 µM, 4 h) as a positive control. Cells were stained with SYTO^{TM} Deep Red nucleic acid stain (1X, 30 min, 37 °C) and CellEvent^{TM} Caspase-3/7 green reagent (3 µM, 30 min, 37 °C) before imaging. Fluorescent emission of CellEvent^{TM} Caspase-3/7 green (laser: 488 nm, 300 ms, 0.8 mW), SYTO^{TM} Deep Red nucleic acid stain (laser: laser: 640 nm, 100 ms, 4.3 mW), and compound **2** (laser: 561 nm, 300 ms, 3.8 mW) were recorded in a Nikon CSU-W1 single camera confocal microscope.

At least 200 cells were quantified and analyzed using CellProfiler 4.2.6 software. The proportion of cells displaying CellEvent™ Caspase-3/7 green and SYTO™ Deep Red signals was established as apoptotic cells. The number of apoptotic cells was represented as a percentage and compared to the controls. The analyses were plotted using Prism 9 (GraphPad), and the data was presented as means, with error bars

representing the standard mean error of three independent experiments (one-way ANOVA and Tukey comparison, P-value > 0.05).

Superoxide production assay

HEK293, HeLa, and MDA-MB-231 cells were seeded on Ibidi plates 1–3 days prior to imaging. The cells were incubated with superoxide sensor HKSOX-1* (10 μM) for 30 min in FluoroBrite DMEM and treated with compounds at indicated concentrations and time. Fluorescent emission of HKSOX-1* (laser: 488 nm, 300 ms, 0.8 mW), was recorded in a Nikon CSU-W1 single-camera confocal microscope. The images were processed by manual analysis in ImageJ software. The analyses were plotted using Prism 9 (GraphPad) and the data was presented as means, with error bars representing the standard mean error of three independent experiments (one-way ANOVA and Tukey comparison, P-value > 0.05).

Quantification of vacuolated cells

HeLa cells were transfected 24–48 h before imaging using KDEL-mTurquoise2 plasmid (#36204) obtained from Addgene. Cells were transfected using the jetPRIME® kit, according to the manufacturer's instructions (Polyplus), and the medium was replaced with a fresh growth medium after 4–5 h. Cells were incubated for 2 h with the inhibitors (zVAD-FMK, 20 μM; CHX, 20 μM; Tiron,100 μM) followed by co-incubation with compound **2** for 2 h in DMEM, then imaged. DMSO (0.1%, 2h) was used as a negative control, and compound **2** as a positive control. Fluorescent emission of the compounds (laser: 561 nm, 300 ms, 3.8 mW) and KDEL-mTurquoise2 (laser: 445 nm, 400 ms, 1.9 mW) were recorded in a Nikon CSU-W1 single camera confocal microscope.

The proportion of cells displaying vacuoles was scored by visually examining at least 300 cells, represented as a percentage, and compared against the controls. Data was plotted as means and error bars represented the standard deviation of three independent experiments (one-way ANOVA and Tukey comparison, *P*-value > 0.05).

Stress-granules formation assay

HeLa cells were transfected 24–48 h before imaging using iRFP-G3BP1 plasmid (#129339) obtained from Addgene. Cells were transfected using the jetPRIME® kit, according to the manufacturer's instructions (Polyplus), and the medium was replaced

with a fresh growth medium after 4–5 h. HeLa cells were incubated with DMSO (0.1%, 2.5 h) or **2** (2 µM, 2.5 h). DMSO was used as a negative control. DMSO control was heated at 43 °C for 0.5 h and was used as a positive control. Fluorescent emission of iRFP-G3BP1 (laser: 640 nm, 100 ms, 3.7 mW) was recorded in a Nikon CSU-W1 single-camera confocal microscope.

Gel-ABPP experiments

HeLa cells were grown in DMEM medium supplemented with fetal bovine serum (FBS, 10%) and antibiotics (1%) at 37 °C in 5% CO_2 environment. The cells were cultured until 90–100% confluence. The lysate was obtained using the CytoBuster™ Protein Extraction Reagent (Merck). The soluble protein concentration was determined using the BCA protein assay (Merck). The lysates were adjusted to 1 mg/mL and treated with DMSO (0.1%) or 100 µM of compounds **1**-**5** for 2 h at 25 °C. After the 2 h incubation with compounds, the mixture was incubated with 5 µM of ATTO 620 maleimide for 1 h at 25 °C. Then, the reaction was quenched with 4x Laemmli Buffer containing 20% β-mercaptoethanol (BME) and incubated for 5 min at 95 °C. The solution was analyzed by SDS-PAGE and in-gel fluorescence scanning.

Proteomics

HeLa cells were grown in DMEM medium supplemented with fetal bovine serum (FBS, 10%) and antibiotics (1%) at 37 °C in 5% CO_2 environment. The cells were cultured until 90–100% confluence. The lysate was obtained using the CytoBuster™ Protein Extraction Reagent (Merck). The soluble protein concentration was determined using the BCA protein assay (Merck). The lysates were adjusted to 1 mg/mL and treated with DMSO (0.1%) or 10 µM of compounds **2-biotin**/**5-biotin**/ **IA-biotin** for 2 h at 25 °C. Then the soluble proteome was incubated with streptavidin magnetic beads (Dynabeads™) for 1 h at room temperature. The beads were washed six times with PBS pH 7.4 containing 0.01% [v/v] Tween™ 20 and submitted to the Functional Genomic Center Zurich (FCGZ). In the FCGZ, the bead-bound proteins were digested, centrifuged, and analyzed by LC-MS/MS analysis (Orbitrap Fusion™ Lumos™ 2). The LC-MS data was processed using the DIA-NN software.[16] The quantification results were extracted from the DIANN main report, containing precursor (charged modified peptide) quantification results. To score potential interactions between observed

proteins (potential prays) and the bait protein the SAINTexpress software[17] was used. Data conversion into SAINTexpress compatible format and visualizations were performed using the R package prolfqua.[18] To select protein candidates a Bayesian false discovery rate (BFDR) threshold of 0.05 and an empirical fold-change score (EFC) threshold of 2 ($\log_2$(EFC) ≥ 1) were used.

Western blots

After the indicated incubation procedure, cells were collected and centrifuged (400×*g*, 4 min, 4 °C), washed with ice-cold PBS and lysed with radio-immunoprecipitation assay (RIPA) buffer (Thermo Fisher Scientific, #89900), supplemented with 2% (v/v) protease inhibitor cocktail (Sigma, #P8340) for 5 min at 4 °C. The resultant suspensions were centrifuged (16,600×*g,* 10 min, 4 °C). Supernatants were retained and total protein content quantified using the bicinchoninic acid (BCA) assay, as per the manufacturer's protocol (Merck-Millipore, #71285-3). The volume of lysate solution corresponding to 50 ng of total protein was mixed with Laemmli buffer (Bio-Rad, #1610747), supplemented with β-mercaptoethanol (5% final concentration) and the resultant mixtures were heated to 95 °C for 5 min. Protein samples were loaded onto 4–20% Mini-PROTEAN TGX Stain-Free Protein Gels (Bio-Rad, #4568094). Gels electrophoresis was carried out for 15 min at 40 V, followed by 55 min at 100 V using a protein ladder as a molecular weight reference (Bio-Rad, #1610374). Gels were transferred to a PVDF membrane (Bio-Rad, #1704156) using the Trans-Blot Turbo Transfer System (Bio-Rad, Mixed MW program). Membranes were blocked in 5% bovine serum albumin (BSA) for 1 h at 20 °C, washed with Tris-buffered saline containing 0.1% Tween-20 (TBST, 3 × 10 min) and incubated overnight at 4 °C with primary antibodies (1:1000 in 5% BSA). The following primary antibodies were used: rabbit anti-GAPDH (Cell Signalling Technology, #5174), mouse anti-GRP78 (Thermo Fisher Scientific, #MA527686). The membranes were then washed with TBST (3 × 10 min) and incubated for 1 h at 20 °C with secondary antibodies (1:5000 in 5% bovine serum albumin). The following secondary antibodies were used: goat anti-rabbit conjugated to AlexaFluor 488 (Thermo Fisher Scientific, #A32731, for GAPDH), goat anti-mouse conjugated to AlexaFluor 488 (Thermo Fisher Scientific, #A32723, for GRP78). The membranes were washed with TBST (3 × 10 min) and TBS (1 × 5 min) and imaged using the ChemiDoc MP System (Bio-Rad).

Synthetic procedures

General procedure for the synthesis of compounds **1**-**5**, **2-NH$_2$**, **2-alkyne**, and **5-alkyne**:

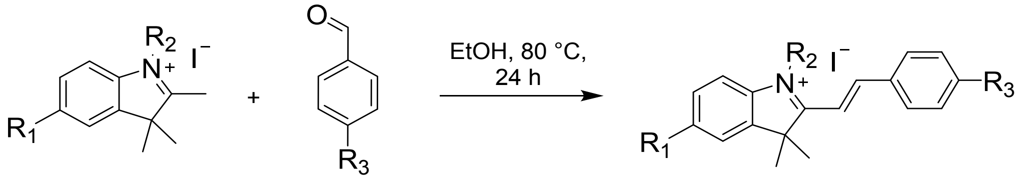

Indoleninium (1 mmol) and aldehyde (1 mmol) were combined in EtOH (10 mL) and stirred at 80 °C for 24 h. The solvent was evaporated, and the crude residue was purified by flash column chromatography (SiO; CH_2Cl_2 to CH_2Cl_2/MeOH 90:10) to give the product hemicyanine.[19,20]

(*E*)-5-cyano-2-(4-hydroxystyryl)-1,3,3-trimethyl-3*H*-indol-1-ium iodide (**1**):

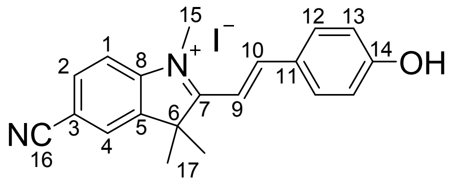

The compound was obtained as a bright orange solid (310 mg, 78%). ^{1}H NMR (500 MHz, DMSO): δ 11.04 (s, 1H), 8.48 (d, J = 16.0 Hz, 1H), 8.43 (d, J = 1.5 Hz, 1H), 8.18 (d, J = 8.8 Hz, 2H), 8.12 (dd, J = 8.3, 1.5 Hz, 1H), 8.01 (d, J = 8.3 Hz, 1H), 7.48 (d, J = 16.0 Hz, 1H), 6.98 (d, J = 8.8 Hz, 2H), 4.07 (s, 3H), 1.80 (s, 6H).^{13}C NMR (126 MHz, DMSO): δ 183.55, 164.22, 156.34, 145.32, 143.84, 134.44, 133.62, 126.95, 126.06, 118.44, 116.65, 115.59, 110.36, 109.11, 51.82, 34.15, 25.51. HR-ESI-MS: m/z 303.14904 [M]$^+$, (calcd for $C_{20}H_{19}ON_2^+$, 303.14919).

(*E*)-2-(4-hydroxystyryl)-1,3,3-trimethyl-5-(trifluoromethyl)-3*H*-indol-1-ium iodide (**2**):

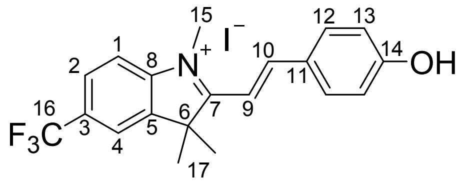

The compound was obtained as a dark red solid (100 mg, 18%). ^{1}H NMR (500 MHz, DMSO): δ 10.98 (s, 1H), 8.42 (d, J = 15.8 Hz, 1H), 8.31 (s, 1H), 8.31 (d, J = 1.6 Hz, 1H), 8.14 (d, J = 8.7 Hz,

2H), 8.00 – 7.92 (m, 2H), 7.39 (d, J = 15.8 Hz, 1H), 6.90 (d, J = 8.6 Hz, 2H), 4.04 (s, 3H), 1.81 (s, 6H).^{13}C NMR (126 MHz, DMSO): δ 182.10, 165.91, 154.93, 145.13, 143.70, 134.49, 126.38, 125.68, 125.21, 123.05, 120.20, 117.38, 114.79, 107.93, 51.60, 33.82, 25.65. HR-ESI-MS: m/z 346.14153 $[M]^+$, (calcd for $C_{20}H_{19}ONF_3^+$, 346.14133).

(*E*)-5-chloro-2-(4-hydroxystyryl)-1,3,3-trimethyl-3*H*-indol-1-ium iodide (**3**):

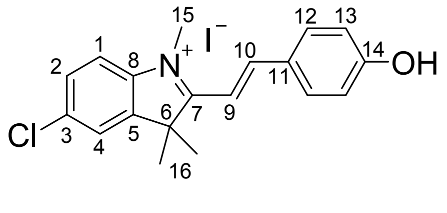

The compound was obtained as an orange solid (100 mg, 80%). ^{1}H NMR (500 MHz, DMSO): δ 7.81 (d, J = 14.1 Hz, 1H), 7.74 (s, 2H), 7.65 (d, J = 2.1 Hz, 1H), 7.37 (dd, J = 8.4, 2.1 Hz, 1H), 7.22 (d, J = 8.4 Hz, 1H), 6.41 (d, J = 14.1 Hz, 1H), 6.20 (d, J = 9.2 Hz, 2H), 3.51 (s, 3H), 1.65 (s, 6H). ^{13}C NMR (126 MHz, DMSO) δ 183.76, 174.38, 169.89, 146.64, 146.61, 142.23, 127.93, 127.24, 124.49, 122.59, 121.41, 110.94, 96.20, 47.95, 30.66, 27.27. HR-ESI-MS: m/z 312.11450 $[M]^+$, (calcd for $C_{19}H_{19}ONCl^+$, 312.11499).

(*E*)-2-(4-hydroxystyryl)-1,3,3-trimethyl-3*H*-indol-1-ium iodide (**4**):

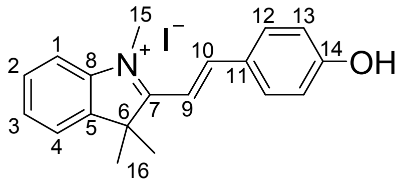

The compound was obtained as a dark red solid (100 mg, 75%). ^{1}H NMR (500 MHz, DMSO) δ 10.82 (s, 1H), 8.36 (d, J = 16.2 Hz, 1H), 8.12 (d, J = 8.8 Hz, 3H), 7.87 – 7.79 (m, 3H), 7.66 – 7.54 (m, 3H), 7.46 (d, J = 16.2 Hz, 1H), 6.96 (d, J = 8.8 Hz, 2H), 4.08 (s, 3H), 1.77 (s, 6H).^{13}C NMR (126 MHz, DMSO): δ 181.40, 163.23, 153.78, 143.19, 141.87, 133.54, 128.85, 128.75, 126.00, 122.77, 116.43, 114.64, 109.29, 51.71, 33.93, 25.64. HR-ESI-MS: m/z 278.15402 $[M]^+$, (calcd for $C_{19}H_{20}ON^+$, 278.15394).

(*E*)-2-(4-hydroxystyryl)-5-methoxy-1,3,3-trimethyl-3*H*-indol-1-ium iodide (**5**):

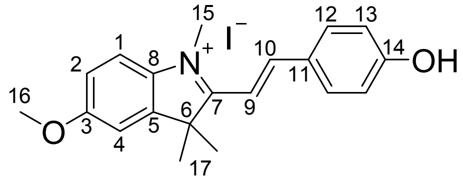

The compound was obtained as an orange solid (200 mg, 76%). ^{1}H NMR (500 MHz, DMSO): δ 10.70 (s, 1H), 8.27 (d, J = 16.2 Hz, 1H), 8.11 – 8.04 (m, 3H), 7.76 (d, J = 8.8 Hz, 1H), 7.50 (d, J = 2.5 Hz, 1H), 7.39 (d, J = 16.3 Hz, 1H), 7.14 (dd, J = 8.8, 2.5 Hz, 1H), 6.94 (d, J = 8.4 Hz,

3H), 4.06 (s, 3H), 3.88 (s, 3H), 1.76 (s, 6H). ^{13}C NMR (126 MHz, DMSO): δ 179.33, 162.72, 160.47, 151.86, 145.35, 135.18, 133.07, 126.07, 116.32, 115.74, 114.54, 109.42, 108.64, 56.12, 51.69, 34.05, 25.69. HR-ESI-MS: m/z 308.16433 $[M]^+$, (calcd for $C_{20}H_{22}O_2N^+$, 308.16451).

(*E*)-2-(4-aminostyryl)-1,3,3-trimethyl-5-(trifluoromethyl)-3*H*-indol-1-ium iodide (**2-NH$_2$**):

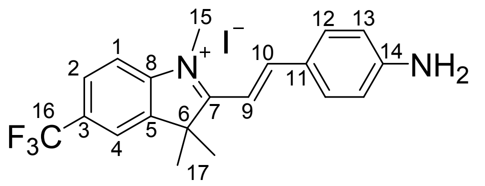

The compound was obtained as a brown solid (70 mg, 22%). ^{1}H NMR (500 MHz, DMSO): δ 8.35 (d, J = 15.4 Hz, 1H), 8.23 (d, J = 1.7 Hz, 1H), 8.03 (d, J = 8.3 Hz, 2H), 7.91 (dd, J = 8.4, 1.7 Hz, 1H), 7.83 (d, J = 8.4 Hz, 1H), 7.34 (s, 2H), 7.17 (d, J = 15.5 Hz, 1H), 6.73 (d, J = 8.6 Hz, 2H), 3.93 (s, 3H), 1.78 (s, 6H). ^{13}C NMR (126 MHz, DMSO): δ 189.46, 180.53, 157.16, 155.99, 145.31, 143.12, 126.26, 125.33, 123.16, 122.70, 120.00, 114.35, 113.74, 103.99, 50.74, 32.98, 26.18. HR-ESI-MS: m/z 345.15666 $[M]^+$, (calcd for $C_{20}H_{20}ON_2F_3^+$, 345.15731).

(*E*)-2-(4-hydroxystyryl)-3,3-dimethyl-1-(pent-4-yn-1-yl)-5-(trifluoromethyl)-3*H*-indol-1-ium iodide (**2-alkyne**):

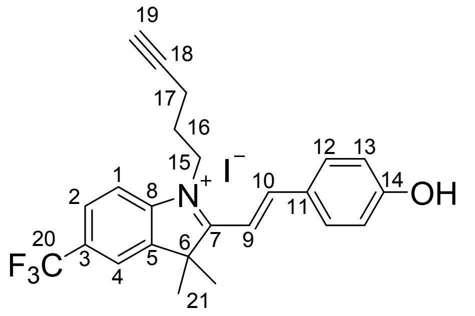

The compound was obtained as a bright pink solid (30 mg, 20%). ^{1}H NMR (500 MHz, DMSO): δ 11.13 (s, 1H), 8.55 (d, J = 15.9 Hz, 1H), 8.37 (d, J = 1.8 Hz, 1H), 8.17 (d, J = 8.8 Hz, 3H), 8.07 – 7.97 (m, 3H), 7.47 (d, J = 16.1 Hz, 1H), 7.00 (d, J = 8.8 Hz, 2H), 4.65 (t, J = 7.4 Hz, 3H), 2.98 (t, J = 2.6 Hz, 1H), 2.42 (td, J = 7.1, 2.7 Hz, 3H), 2.05 (p, J = 7.3 Hz, 3H), 1.85 (s, 6H). ^{13}C NMR (126 MHz, DMSO): δ 183.64, 164.53, 156.90, 144.19, 144.04, 134.55, 126.55, 125.96, 125.13, 122.96, 120.53, 116.78, 115.16, 108.30, 83.34, 72.43, 52.18, 45.42, 26.76, 25.83, 15.18. HR-ESI-MS: m/z 398.17255 $[M]^+$, (calcd for $C_{24}H_{23}ONF_3^+$, 398.17263).

(*E*)-2-(4-hydroxystyryl)-5-methoxy-3,3-dimethyl-1-(pent-4-yn-1-yl)-3*H*-indol-1-ium iodide (**5-alkyne**):

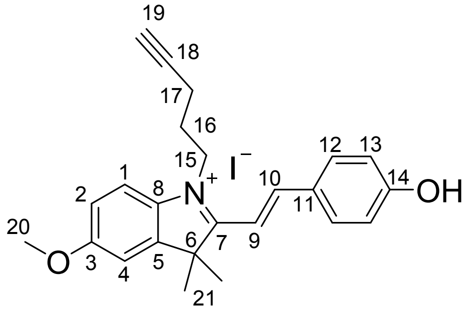

The compound was obtained as a bright red solid (30 mg, 24%).^{1}H NMR (500 MHz, MeOD) δ 8.29 (d, J = 15.9 Hz, 1H), 7.91 (d, J = 8.4 Hz, 2H), 7.67 (d, J = 8.8 Hz, 1H), 7.36 – 7.29 (m, 2H), 7.14 (dd, J = 8.8, 2.5 Hz, 1H), 6.92 (d, J = 8.5 Hz, 2H), 3.92 (s, 3H), 2.58 (d, J = 2.7 Hz, 1H), 2.43 (td, J = 6.5, 2.6 Hz, 2H), 2.15 (p, J = 6.9 Hz, 2H), 1.83 (s, 6H). ^{13}C NMR (126 MHz, MeOD) δ 180.89, 167.57, 162.50, 155.00, 146.62, 135.53, 134.68, 126.74, 118.55, 116.23, 116.03, 109.73, 108.27, 83.61, 71.93, 56.72, 53.25, 46.16, 28.13, 27.16, 16.41. HR-ESI-MS: m/z 360.19520 $[M]^+$, (calcd for $C_{24}H_{26}O_2N^+$, 360.19851).

General procedure for the synthesis of compounds **2-biotin**, **5-biotin**, and **IA-biotin:**

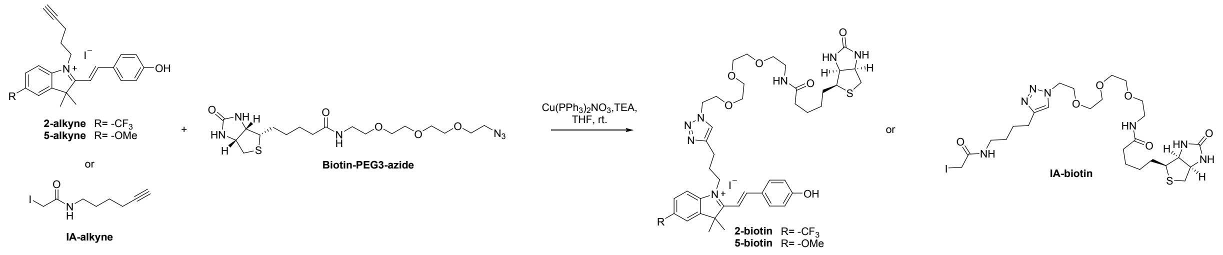

In a flame-dried flask, **2-alkyne**, **5-alkyne**, or **IA-alkyne** (1.0 equiv.) and **biotin-PEG3-azide** (1.5 equiv.) were dissolved in degassed THF (7 mL) and the solution was degassed for an additional hour by bubbling argon through the solution while stirring vigorously. $Cu(PPh_3)_2NO_3$ (0.5 equiv.) and triethylamine (0.5 equiv.) were dissolved in 3 mL of degassed THF and added to the solution and stirred for 24 h at room temperature. Solvents were removed under reduced pressure and the residue was purified via column chromatography (SiO_2; CH_2Cl_2/MeOH,100/0 to 0/100).[20]

2-((*E*)-4-hydroxystyryl)-3,3-dimethyl-1-(3-(1-(13-oxo-17-((3a*S*,4*S*,6a*R*)-2-oxohexahydro-1*H*-thieno[3,4-d]imidazol-4-yl)-3,6,9-trioxa-12-azaheptadecyl)-1*H*-1,2,3-triazol-4-yl)propyl)-5-(trifluoromethyl)-3*H*-indol-1-ium iodide (**2-biotin**):

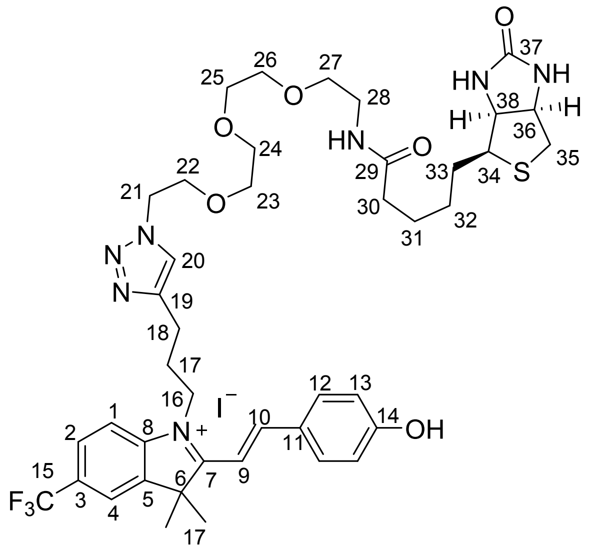

The compound was obtained as a bright pink solid (13 mg, 70%). ^{1}H NMR (500 MHz, $CDCl_3$) δ 7.66 – 7.53 (m, 4H), 7.50 (d, J = 1.7 Hz, 1H), 7.12 (d, J = 8.3 Hz, 1H), 6.58 (d, J = 9.4 Hz, 2H), 6.54 (d, J = 13.7 Hz, 1H), 4.55 (t, J = 5.0 Hz, 2H), 4.47 (dd, J = 7.8, 4.8 Hz, 1H), 4.29 (dd, J = 7.8, 4.6 Hz, 1H), 4.20 (t, J = 7.9 Hz, 2H), 3.89 (t, J = 5.1 Hz, 2H), 3.67 – 3.57 (m, 10H), 3.54 (t, J = 5.3 Hz, 3H), 3.41 (t, J = 5.2 Hz, 2H), 3.12 (td, J = 7.4, 4.5 Hz, 1H), 2.92 – 2.84 (m, 3H), 2.71 (d, J = 12.9 Hz, 1H), 2.33 – 2.13 (m, 5H), 1.71 (s, 6H), 1.68 – 1.52 (m, 6H), 1.50 – 1.35 (m, 4H). ^{13}C NMR (126 MHz, $CDCl_3$) δ 173.46, 173.38, 163.69, 146.81, 146.03, 145.55, 140.79, 126.55, 125.44, 123.84, 123.29, 122.74, 119.26, 109.46, 98.00, 70.57, 70.54, 70.51, 70.23, 69.96, 69.61, 65.99, 61.91, 61.80, 60.25, 60.13, 55.57, 50.89, 48.25, 43.41, 40.65, 39.28, 39.16, 35.97, 35.92, 29.84, 28.45, 28.25, 28.22, 25.85, 25.65, 22.37. HR-ESI-MS: m/z 842.38835 [M]$^+$, (calcd for $C_{42}H_{55}F_3O_6N_7S^+$, 842.38811).

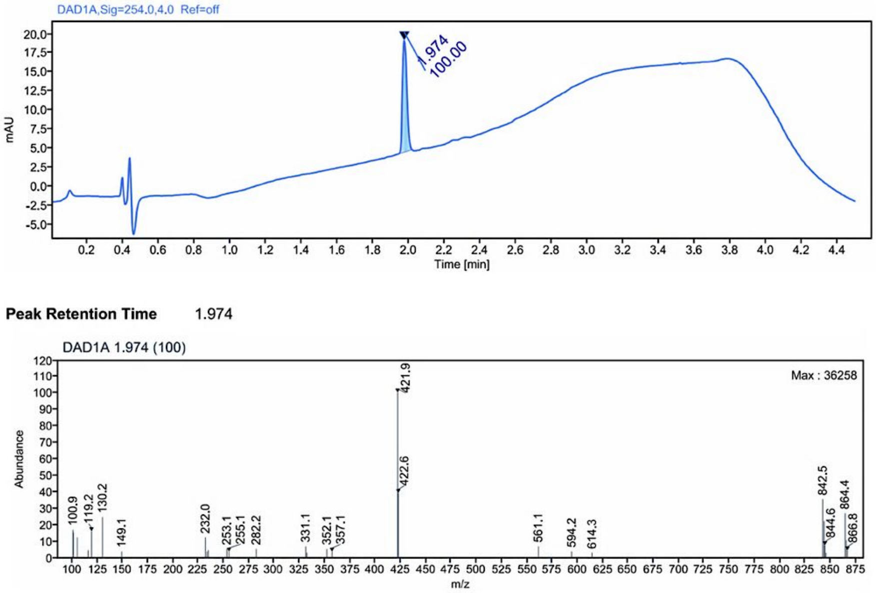

Figure S15. LC-MS chromatogram of compound **2-biotin**

2-((*E*)-4-hydroxystyryl)-5-methoxy-3,3-dimethyl-1-(3-(1-(13-oxo-17-((3a*S*,4*S*,6a*R*)-2-oxohexahydro-1*H*-thieno[3,4-d]imidazol-4-yl)-3,6,9-trioxa-12-azaheptadecyl)-1*H*-1,2,3-triazol-4-yl)propyl)-3*H*-indol-1-ium iodide (**5-biotin**):

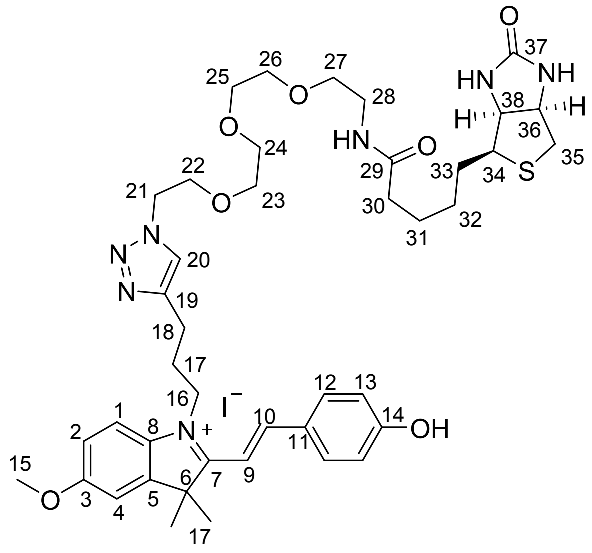

The compound was obtained as a bright purple solid (11 mg, 58%). ^{1}H NMR (500 MHz, MeOD) δ 8.08 (d, *J* = 14.9 Hz, 1H), 7.86 (s, 1H), 7.71 (d, *J* = 7.3 Hz, 2H), 7.34 (d, *J* = 8.7 Hz, 1H), 7.18 (d, *J* = 2.5 Hz, 1H), 7.01 (dd, *J* = 8.7, 2.5 Hz, 1H), 6.76 (d, *J* = 14.9 Hz, 1H), 6.57 (d, *J* = 8.8 Hz, 2H), 4.56 (t, *J* = 5.0 Hz, 2H), 4.46 (dd, *J* = 7.9, 4.8 Hz, 1H), 4.34 (t, *J* = 7.6 Hz, 2H), 4.27 (dd, *J* = 7.9, 4.5 Hz, 1H), 3.90 – 3.87 (m, 2H), 3.87 (s, 3H), 3.63 – 3.50 (m, 8H), 3.47 (t, *J* = 5.5 Hz, 2H), 3.16 (ddd, *J* = 8.9, 5.7, 4.3 Hz, 1H), 2.93 – 2.86 (m, 3H), 2.68 (d, *J* = 12.7 Hz, 1H), 2.36 (s, 1H), 2.27 (p, *J* = 7.2 Hz, 2H), 2.17 (t, *J* = 7.4 Hz, 2H), 1.75 (s, 6H), 1.73 – 1.51 (m, 5H), 1.45 – 1.35 (m, 3H). ^{13}C NMR (126 MHz, MeOD) δ 182.97, 177.16, 176.05, 166.07, 160.65, 154.33, 147.33, 144.96, 136.36, 132.10, 129.99, 128.38, 127.90, 124.54, 123.21, 122.37, 115.12, 113.81, 109.90, 101.14, 71.51, 71.45, 71.37, 71.23, 70.54, 70.38, 63.36, 61.61, 56.99, 56.48, 51.56, 51.39, 44.99, 41.05, 40.32, 36.71, 29.75, 29.49, 27.96, 27.92, 26.83, 23.16. HR-ESI-MS: m/z 804.41263 $[M]^{+}$, (calcd for $C_{42}H_{58}O_{7}N_{7}S^{+}$, 804.41129).

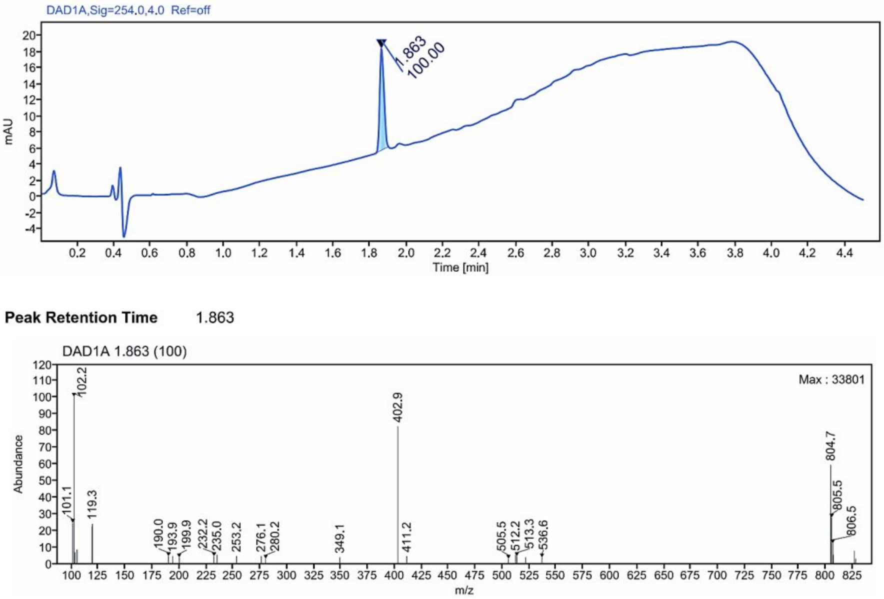

Figure S16. LC-MS chromatogram of compound **5-biotin**

***N*-(2-(2-(2-(2-(4-(4-(2-iodoacetamido)butyl)-1*H*-1,2,3-triazol-1-yl)ethoxy)ethoxy)ethoxy)ethyl)-5-((3a*S*,4*S*,6a*R*)-2-oxohexahydro-1*H*-thieno[3,4-d]imidazol-4-yl)pentanamide** (**IA-biotin**):

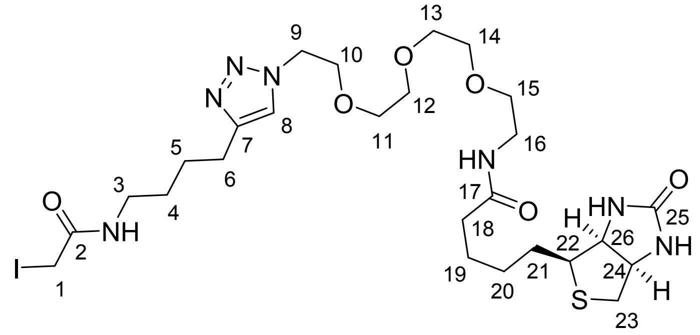

The compound was obtained as a colorless sticky solid (13 mg, 24%). ^{1}H NMR (500 MHz, MeOD) δ 7.81 (s, 1H), 4.58 – 4.48 (m, 5H), 4.32 (dd, *J* = 7.8, 4.4 Hz, 1H), 3.89 (t, *J* = 5.0 Hz, 2H), 3.72 – 3.58 (m, 9H), 3.54 (t, *J* = 5.5 Hz, 2H), 3.40 – 3.33 (m, 4H), 3.29 – 3.18 (m, 3H), 2.93 (dd, *J* = 12.8, 5.0 Hz, 1H), 2.76 – 2.68 (m, 3H), 2.22 (t, *J* = 7.4 Hz, 2H), 1.79 – 1.52 (m, 8H), 1.43 (p, *J* = 7.7 Hz, 2H). ^{13}C NMR (126 MHz, MeOD) δ 176.30, 171.45, 166.08, 148.72, 124.11, 71.46, 71.39, 71.37, 71.19, 70.54, 70.40, 63.35, 61.58, 56.99, 51.30, 41.09, 40.54, 40.30, 36.74, 29.72, 29.45, 29.43, 27.68, 26.81, 25.83, -1.83. HR-ESI-MS: m/z 732.20168 [M]$^+$, (calcd for $C_{26}H_{44}IO_6N_7SNa^+$, 732.20107).

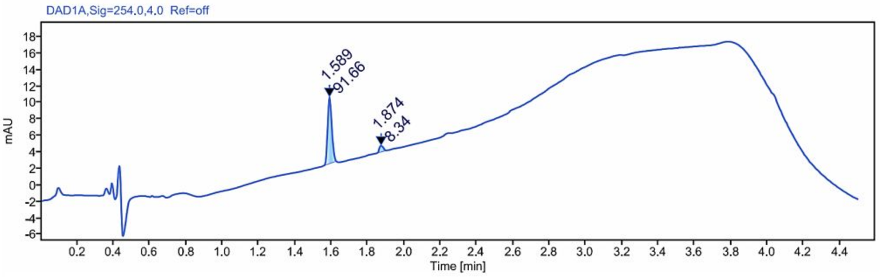

Peak Retention Time 1.589

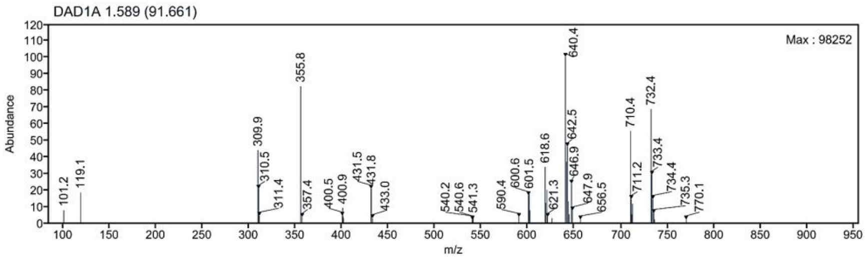

Figure S17. LC-MS chromatogram of compound **IA-biotin**

NMR spectra.

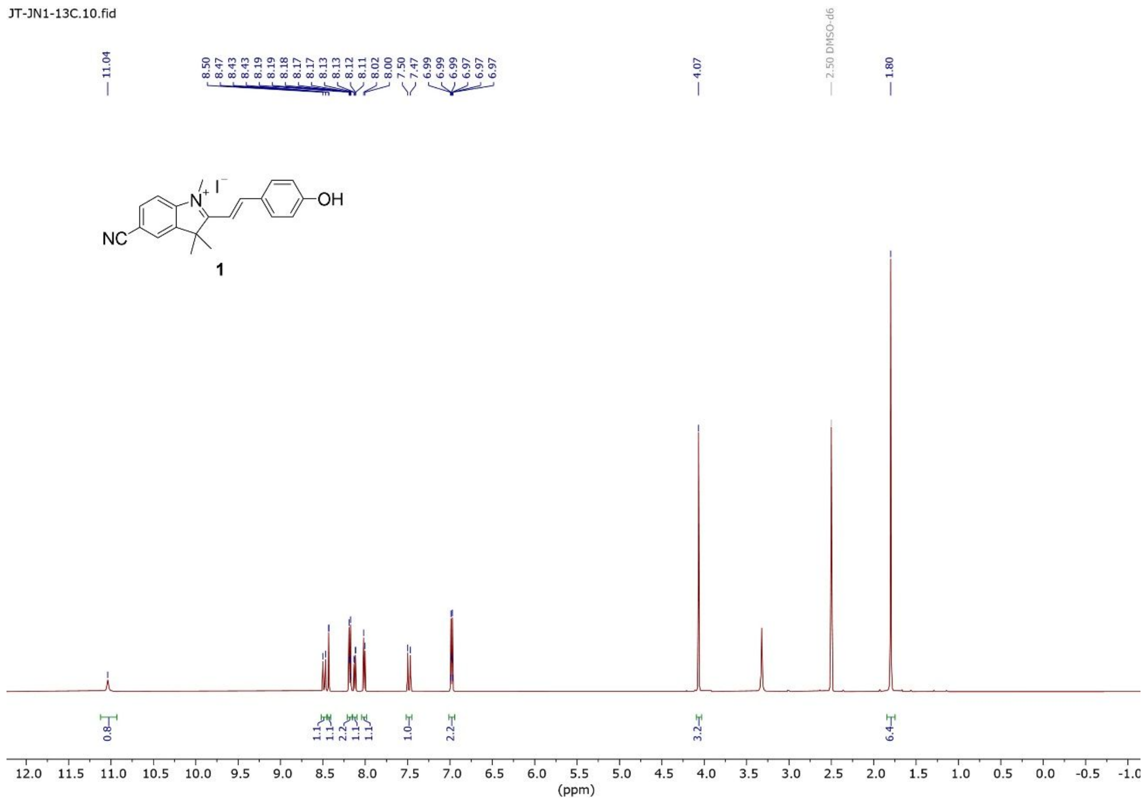

Figure S18. ^{1}H NMR spectra (DMSO-*d*6, 500 MHz) of compound **1**.

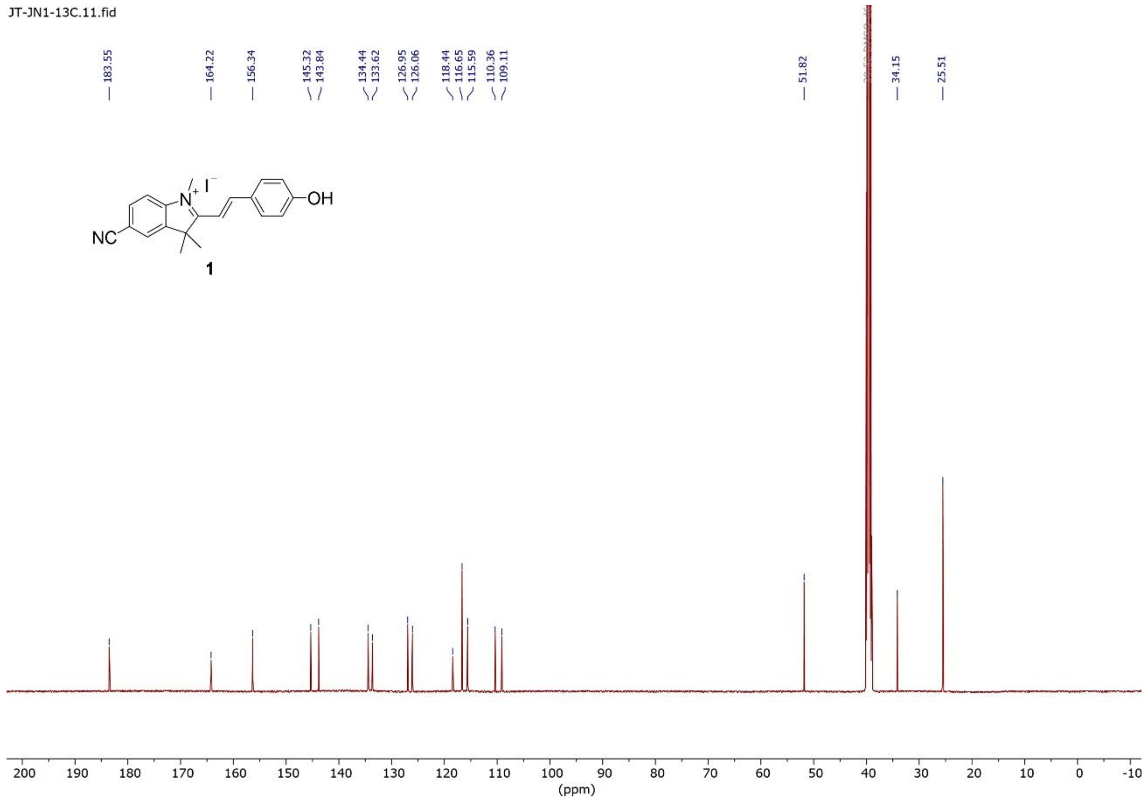

Figure S19. $^{13}C\{^{1}H\}$ NMR spectra (DMSO-*d*6, 126 MHz) of compound **1**.

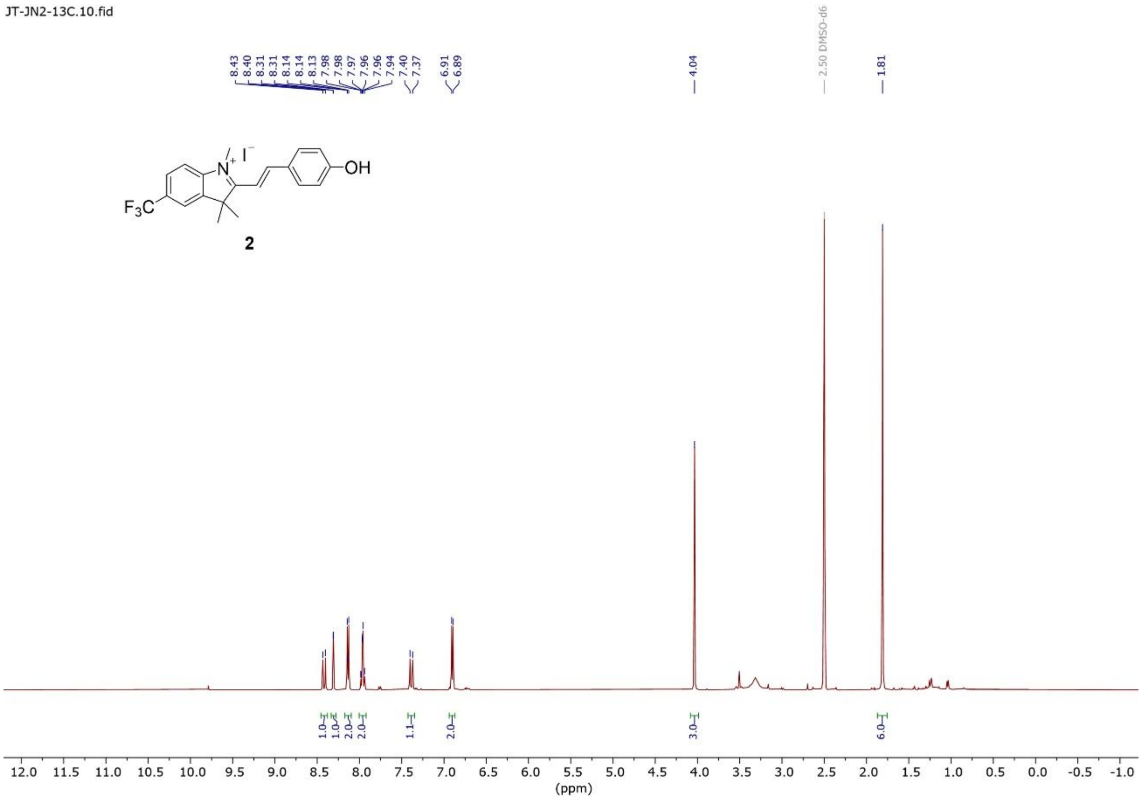

Figure S20. ^1H NMR spectra (DMSO-*d*6, 500 MHz) of compound **2**.

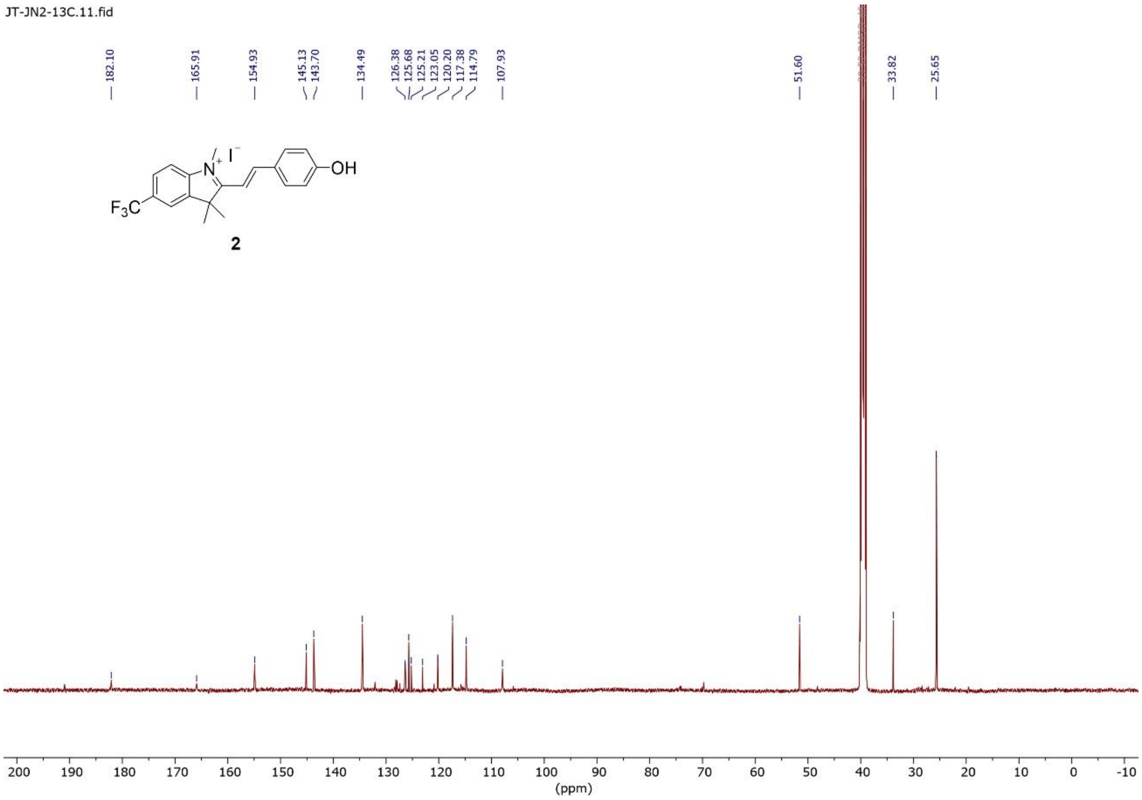

Figure S21. $^{13}C\{^1H\}$ NMR spectra (DMSO-*d*6, 126 MHz) of compound **2**.

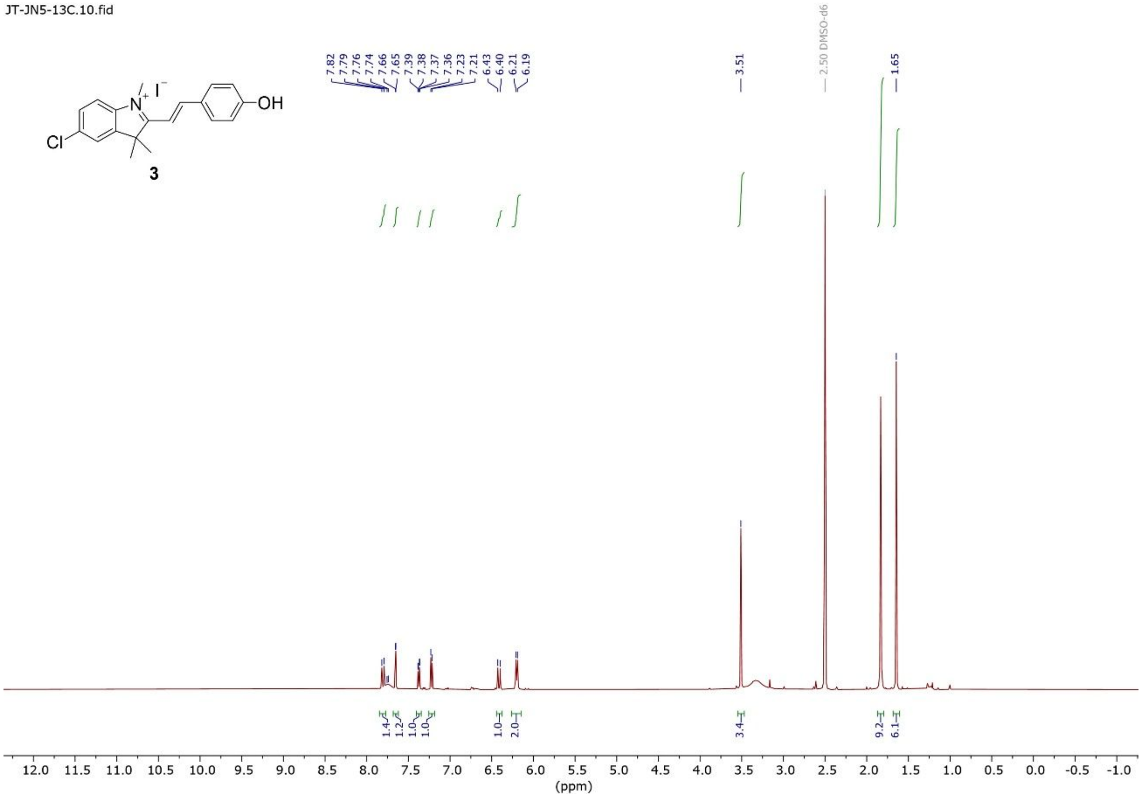

Figure S22. ^{1}H NMR spectra (DMSO-*d*6, 500 MHz) of compound **3**.

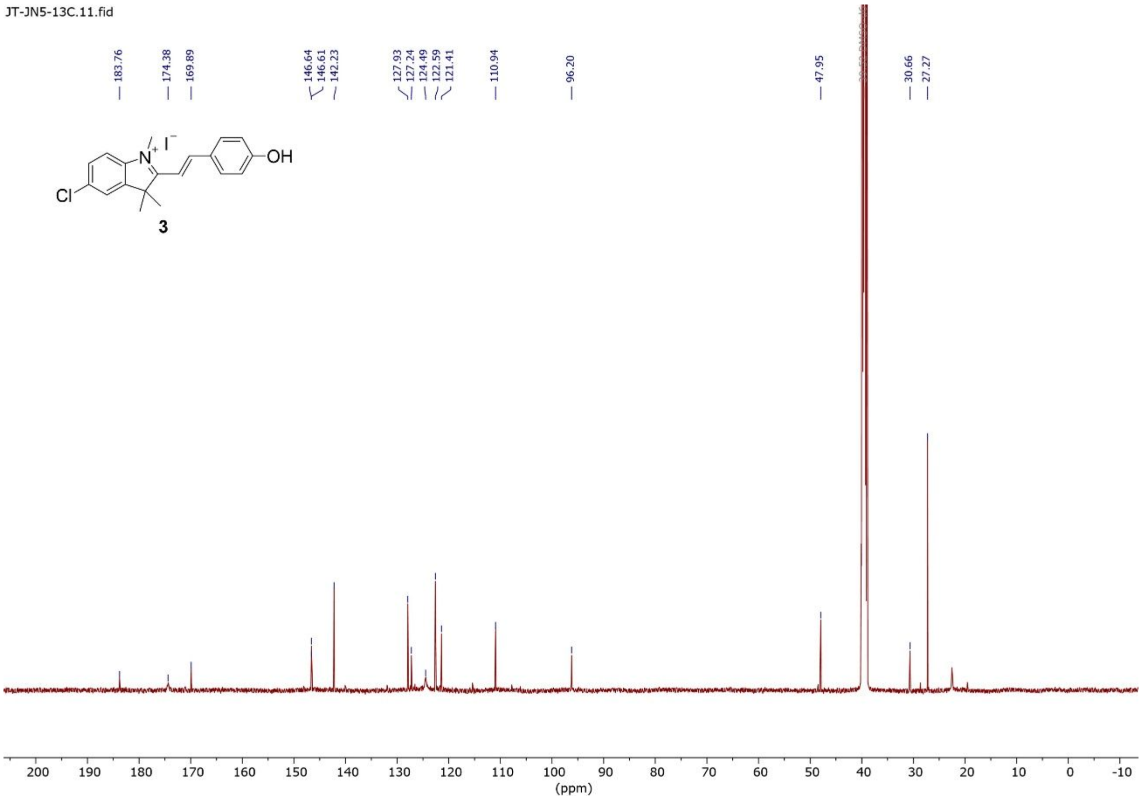

Figure S23. $^{13}C\{^{1}H\}$ NMR spectra (DMSO-*d*6, 126 MHz) of compound **3**.

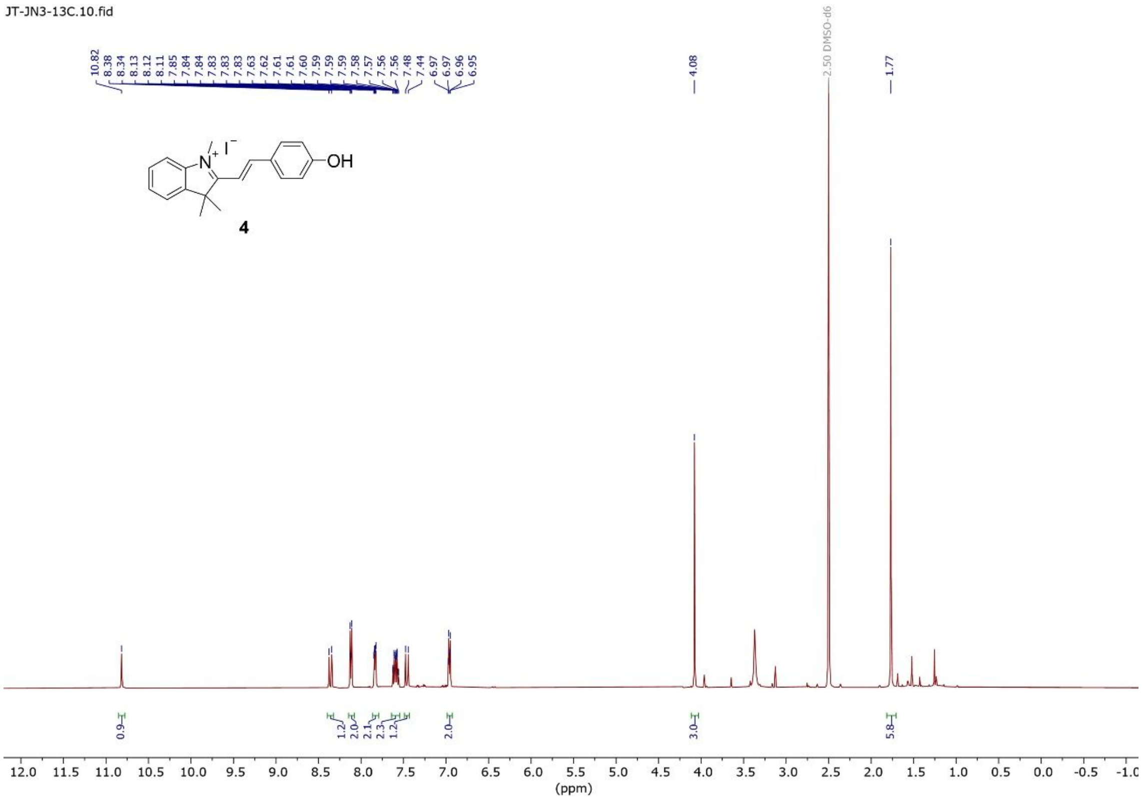

Figure S24. ^{1}H NMR spectra (DMSO-*d*6, 500 MHz) of compound **4**.

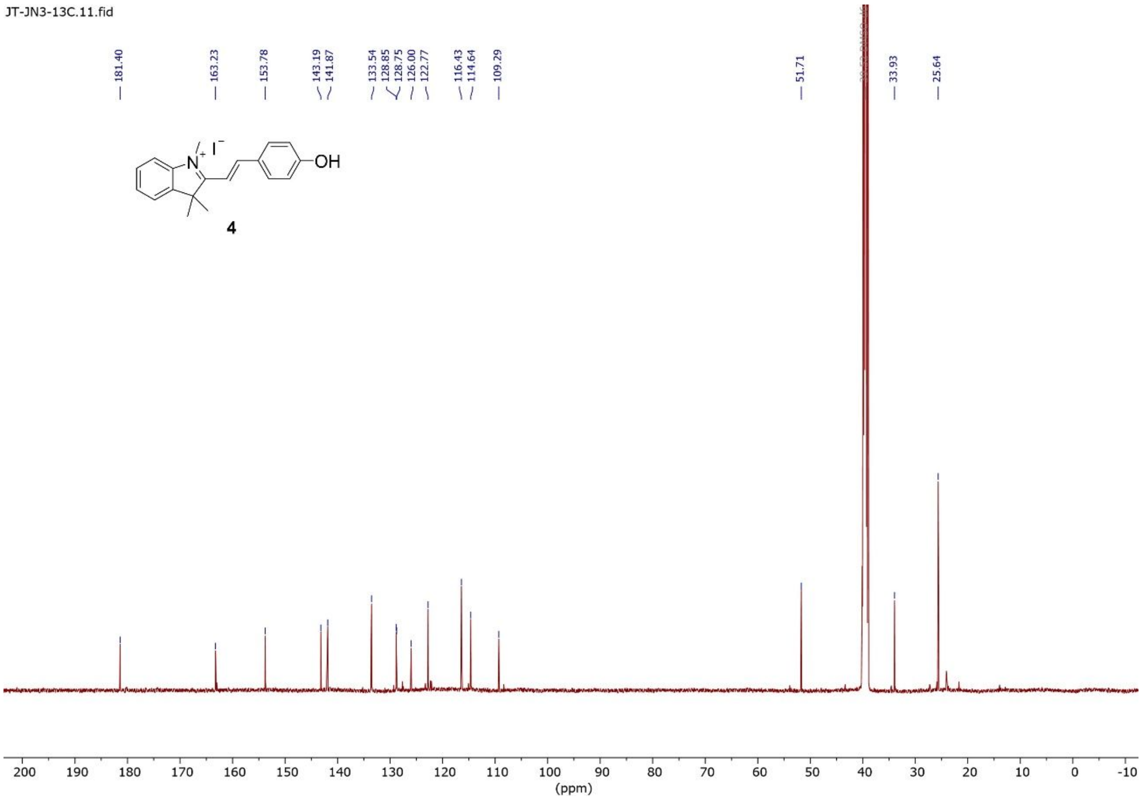

Figure S25. $^{13}C\{^{1}H\}$ NMR spectra (DMSO-*d*6, 126 MHz) of compound **4**.

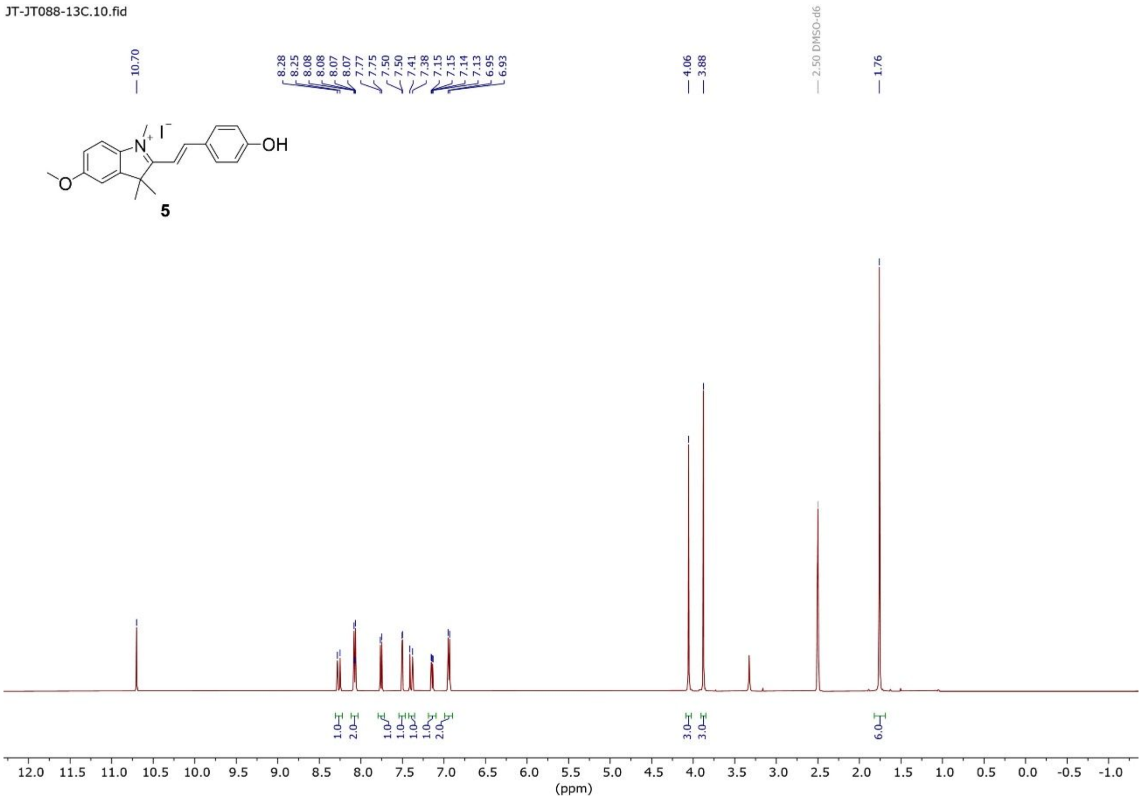

Figure S26. ^{1}H NMR spectra (DMSO-*d*6, 500 MHz) of compound **5**.

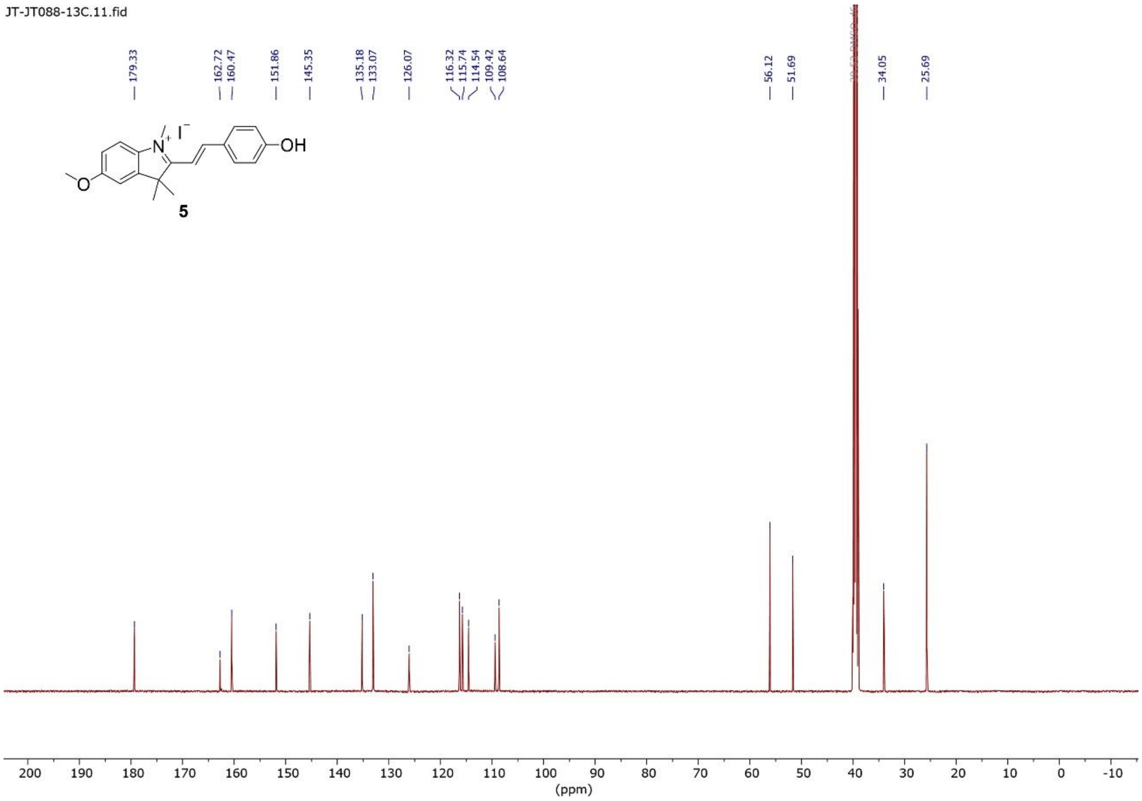

Figure S27. $^{13}C\{^{1}H\}$ NMR spectra (DMSO-*d*6, 126 MHz) of compound **5**.

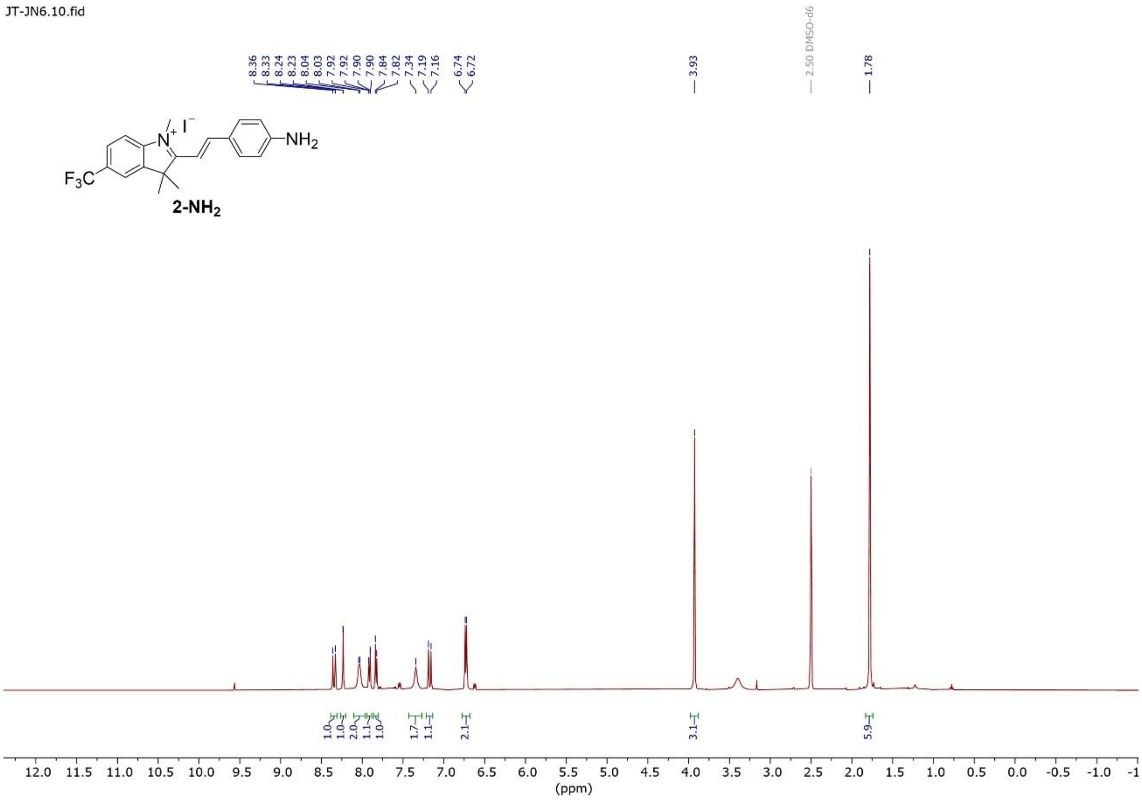

Figure S28. ^{1}H NMR spectra (DMSO-*d*6, 500 MHz) of compound **2-NH₂**.

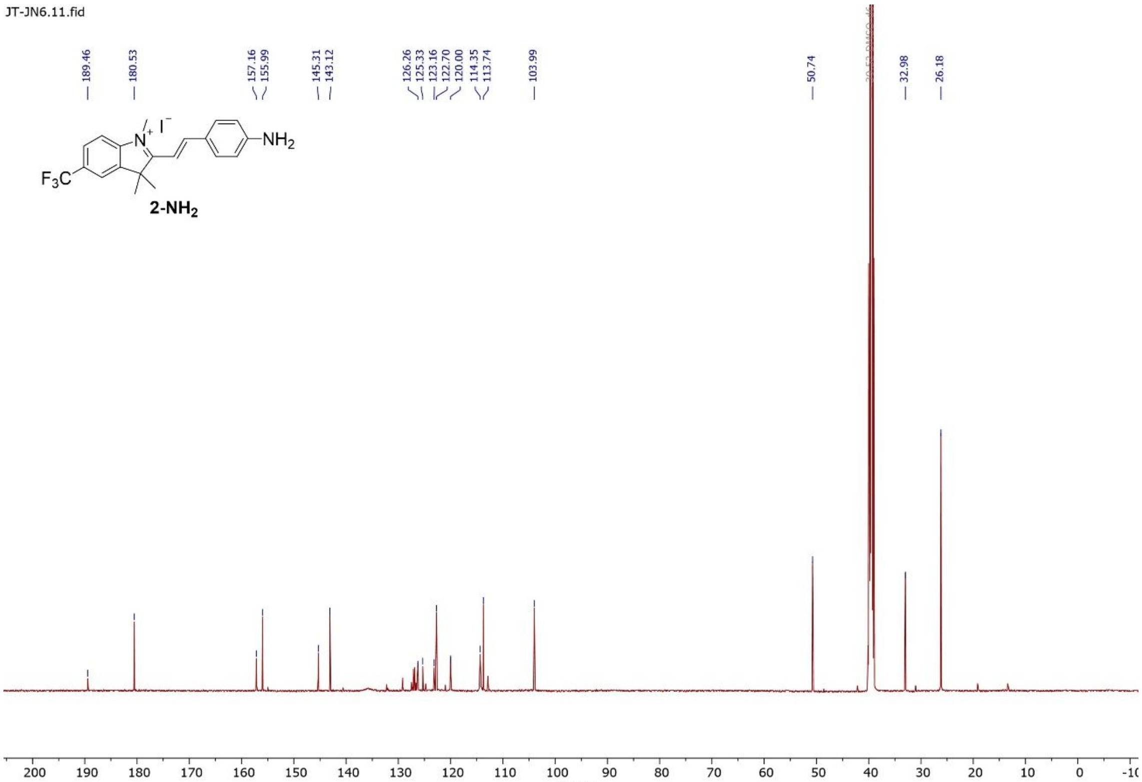

Figure S29. $^{13}C\{^{1}H\}$ NMR spectra (DMSO-*d*6, 126 MHz) of compound **2-NH₂**.

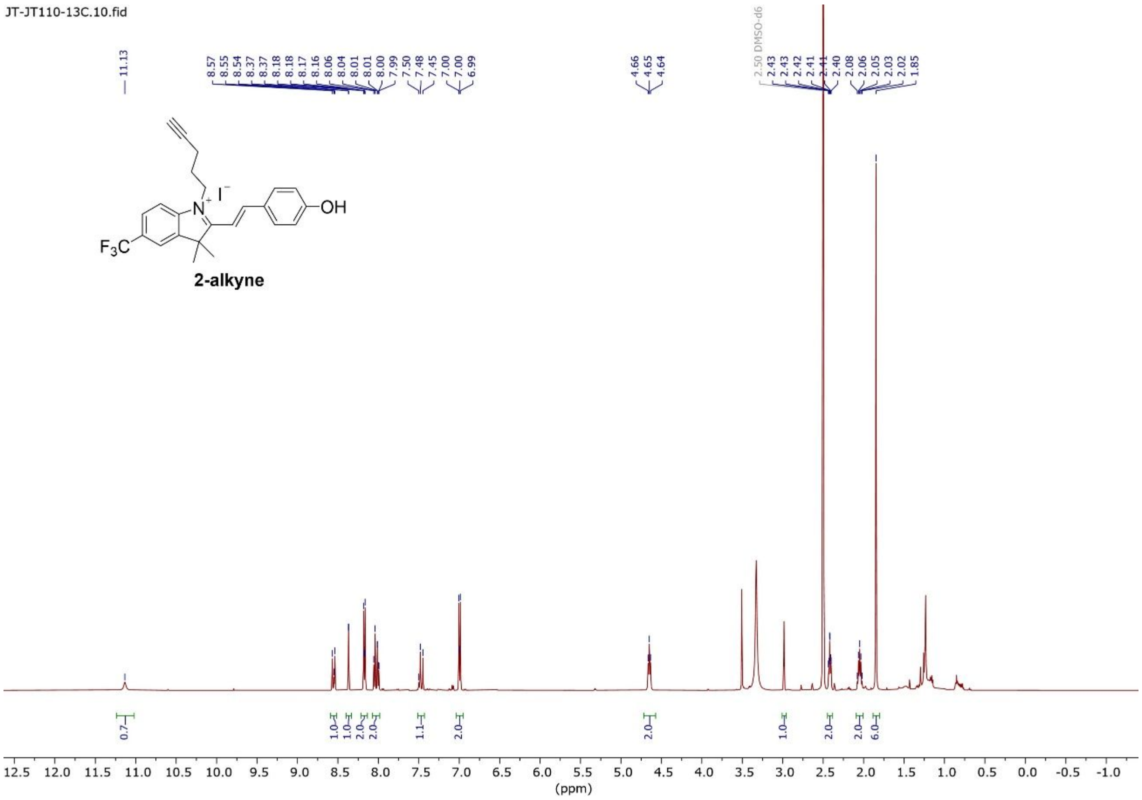

Figure S30. ^{1}H NMR spectra (DMSO-*d*6, 500 MHz) of compound **2-alkyne**.

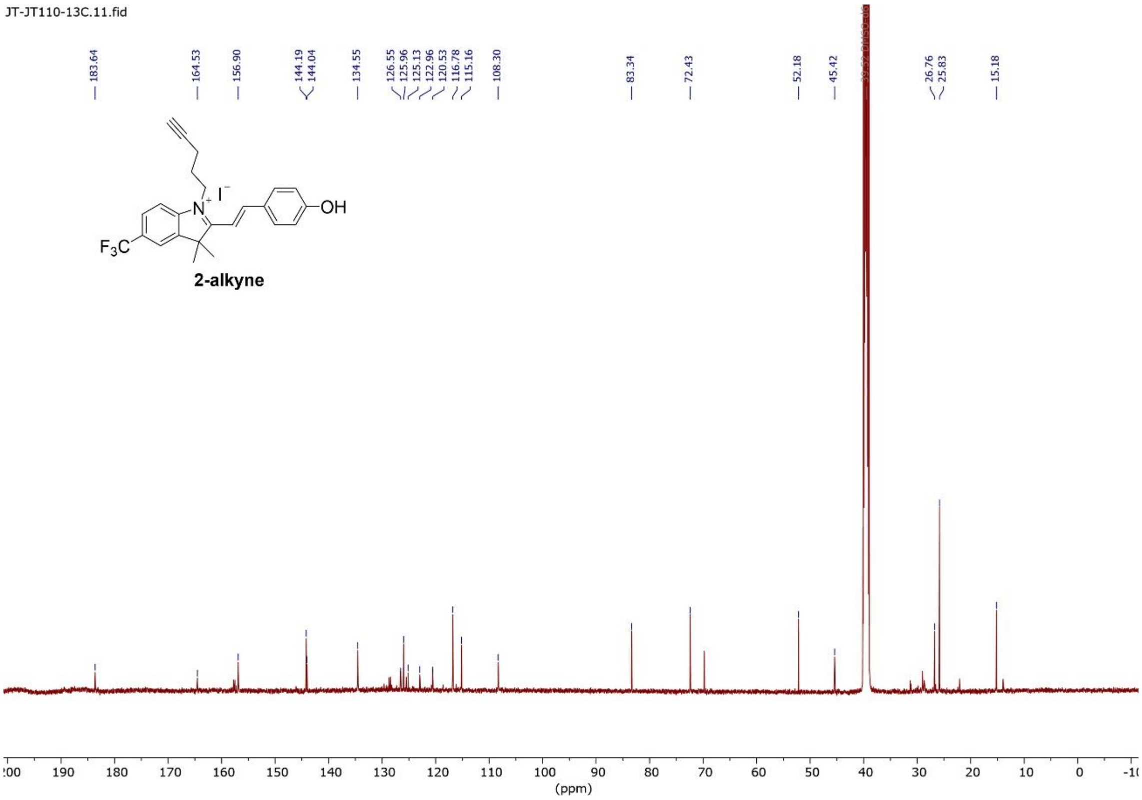

Figure S31. $^{13}C\{^{1}H\}$ NMR spectra (DMSO-*d*6, 126 MHz) of compound **2-alkyne**.

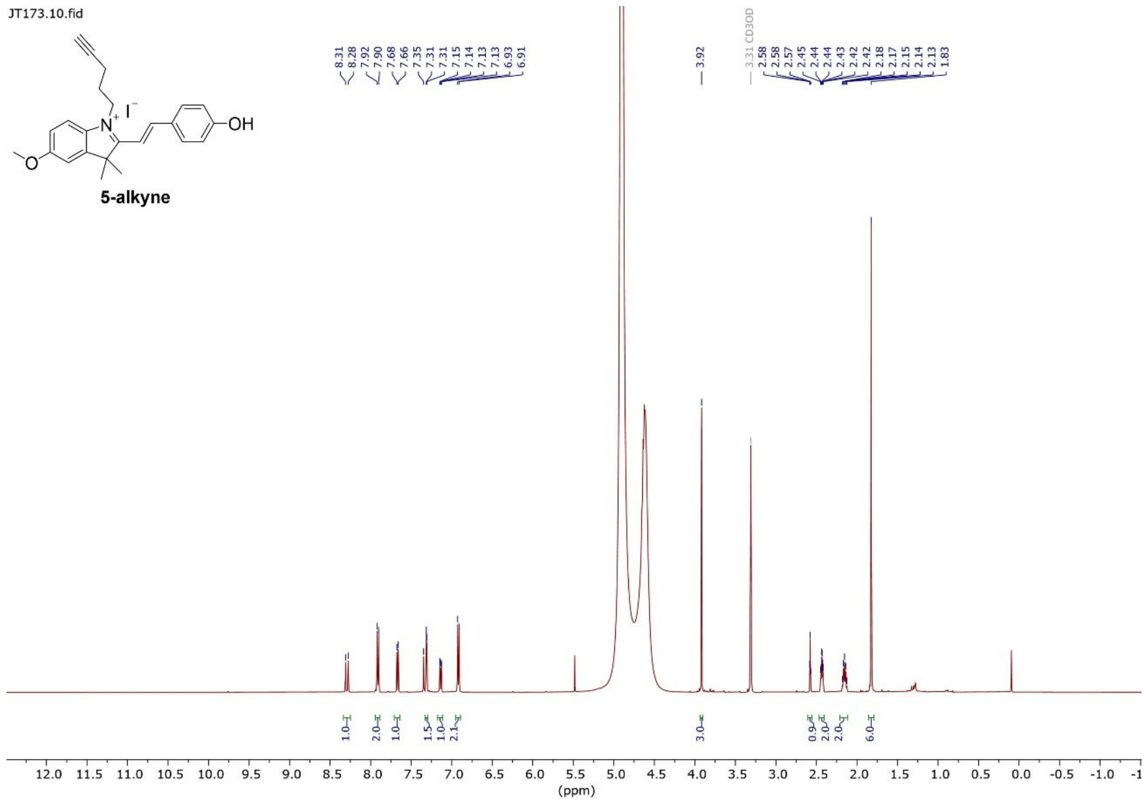

Figure S32. ^{1}H NMR spectra (methanol-*d*4, 500 MHz) of compound **5-alkyne**.

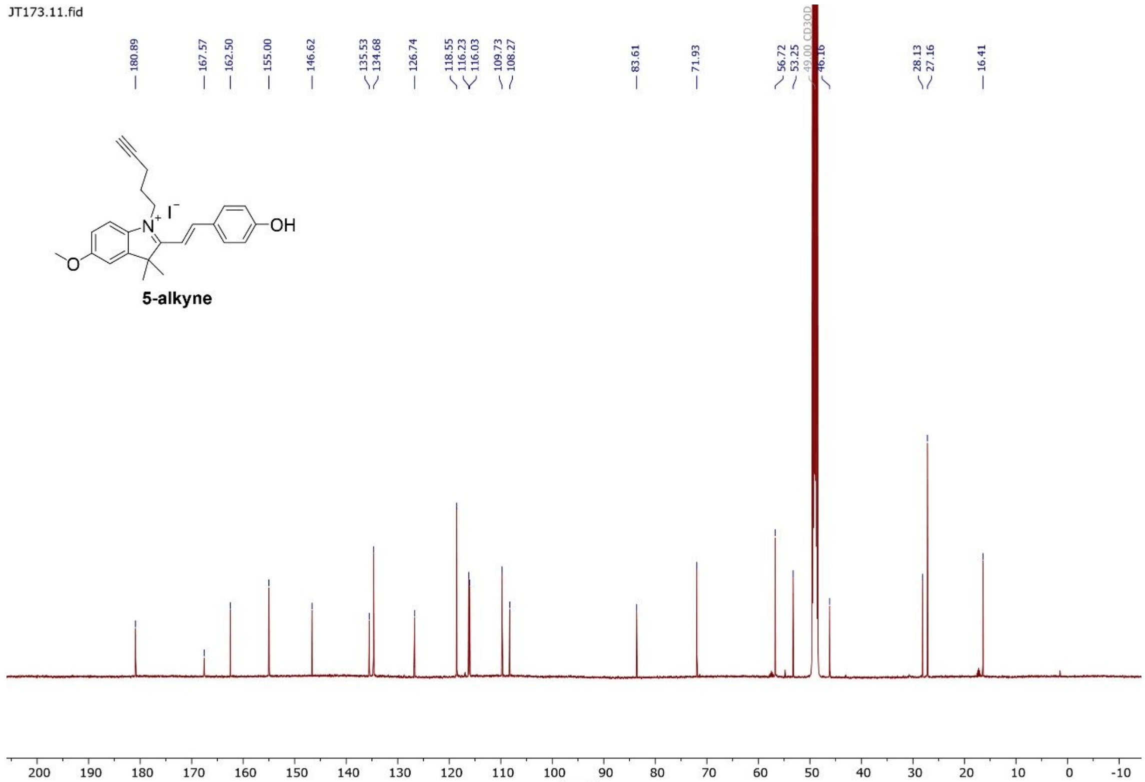

Figure S33. $^{13}C\{^{1}H\}$ NMR spectra (methanol-*d*4, 126 MHz) of compound **5-alkyne**.

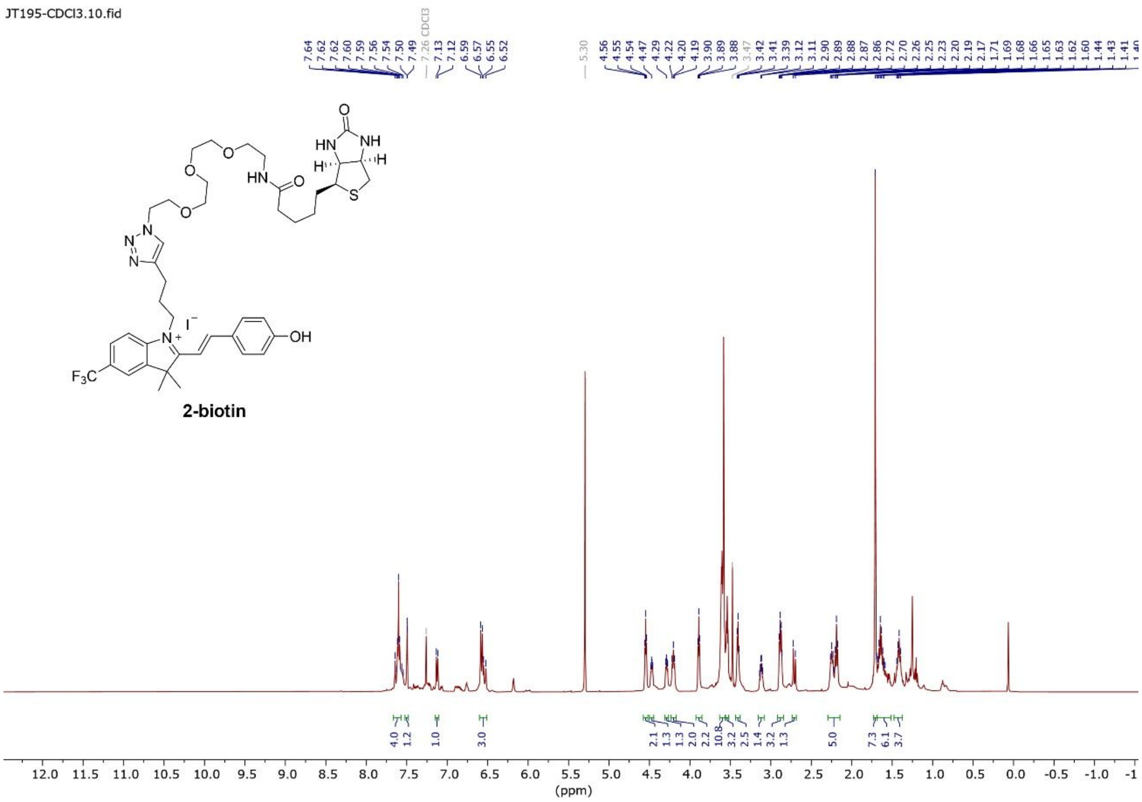

Figure S34. ^{1}H NMR spectra ($CDCl_3$, 500 MHz) of compound **2-biotin**.

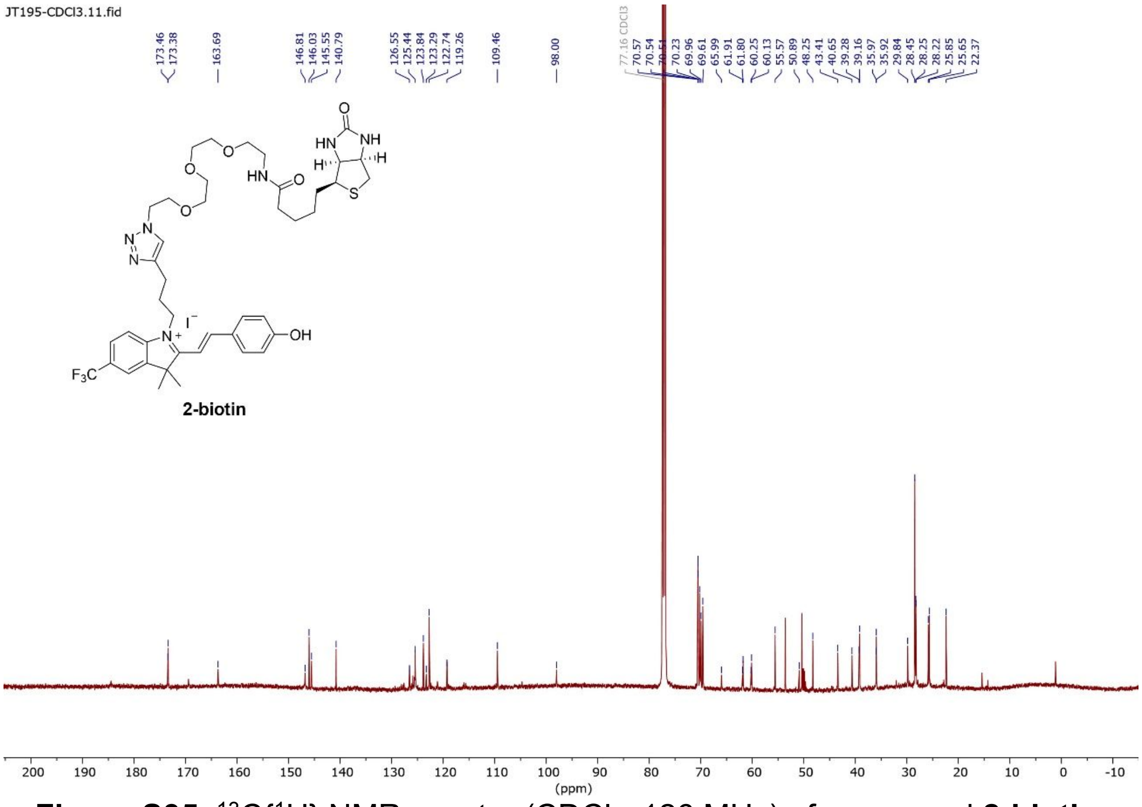

Figure S35. $^{13}C\{^{1}H\}$ NMR spectra ($CDCl_3$, 126 MHz) of compound **2-biotin**.

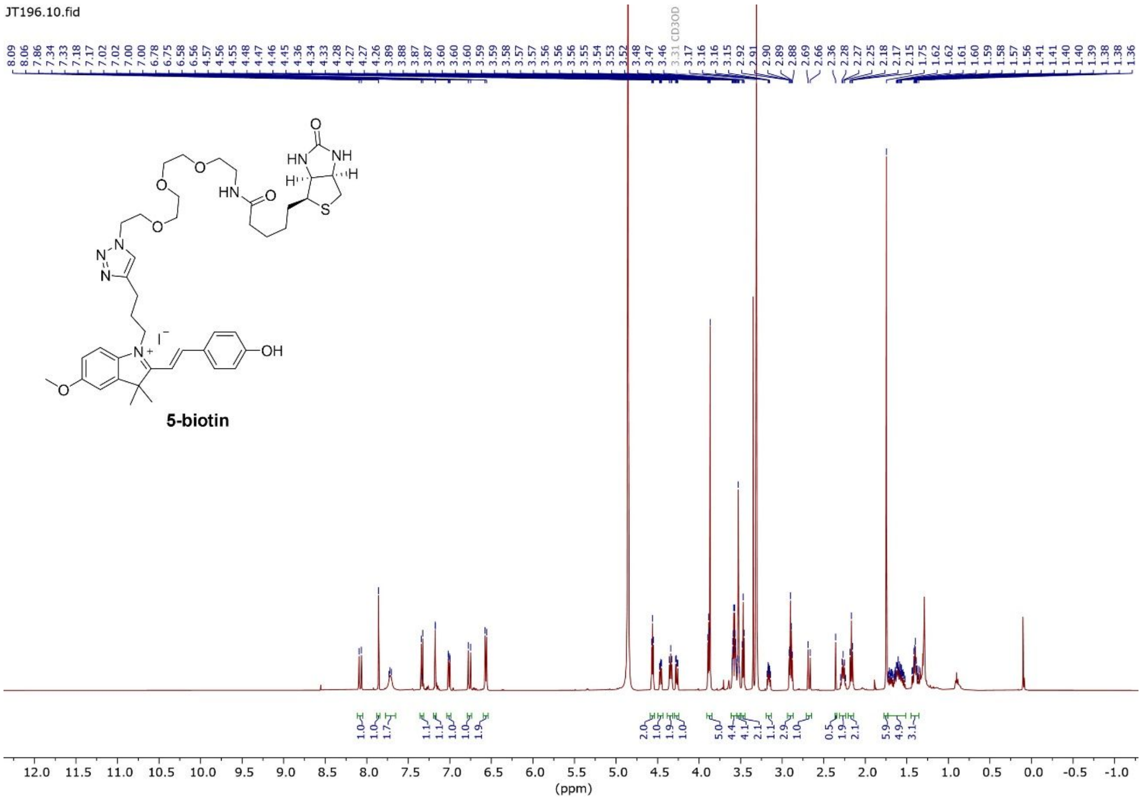

Figure S36. ^{1}H NMR spectra (methanol-*d*4, 500 MHz) of compound **5-biotin**.

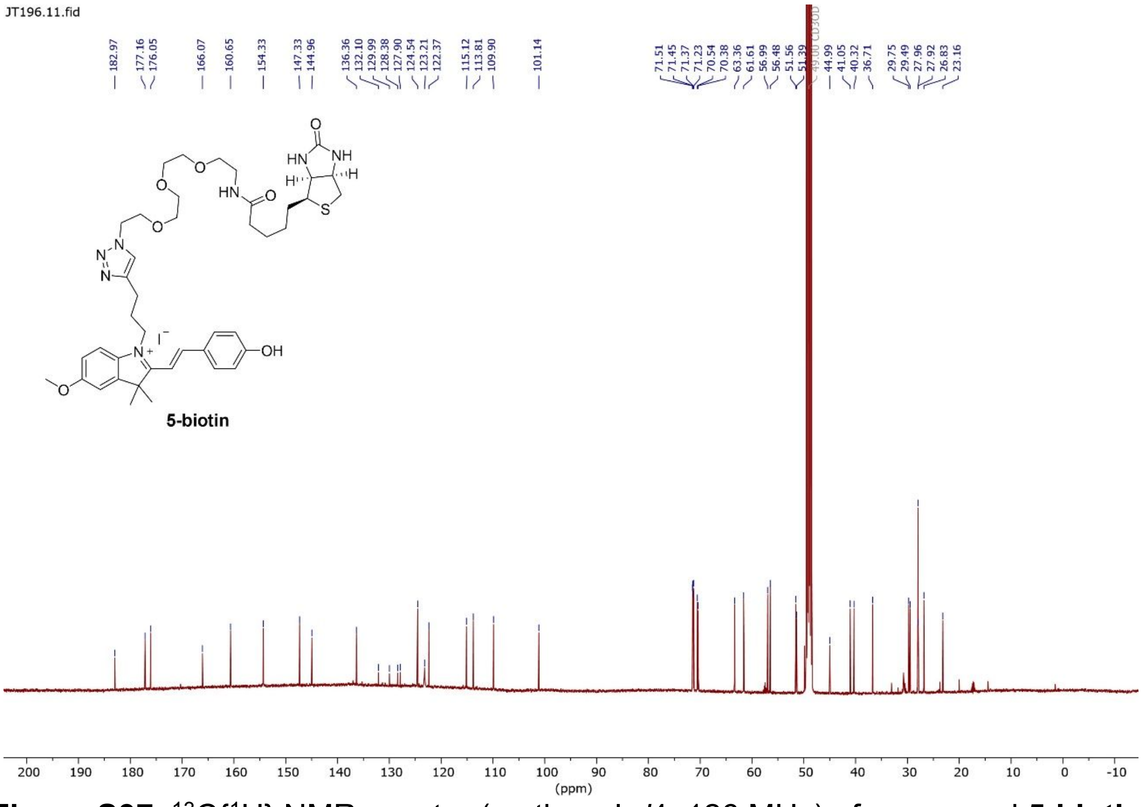

Figure S37. $^{13}C\{^{1}H\}$ NMR spectra (methanol-*d*4, 126 MHz) of compound **5-biotin**.

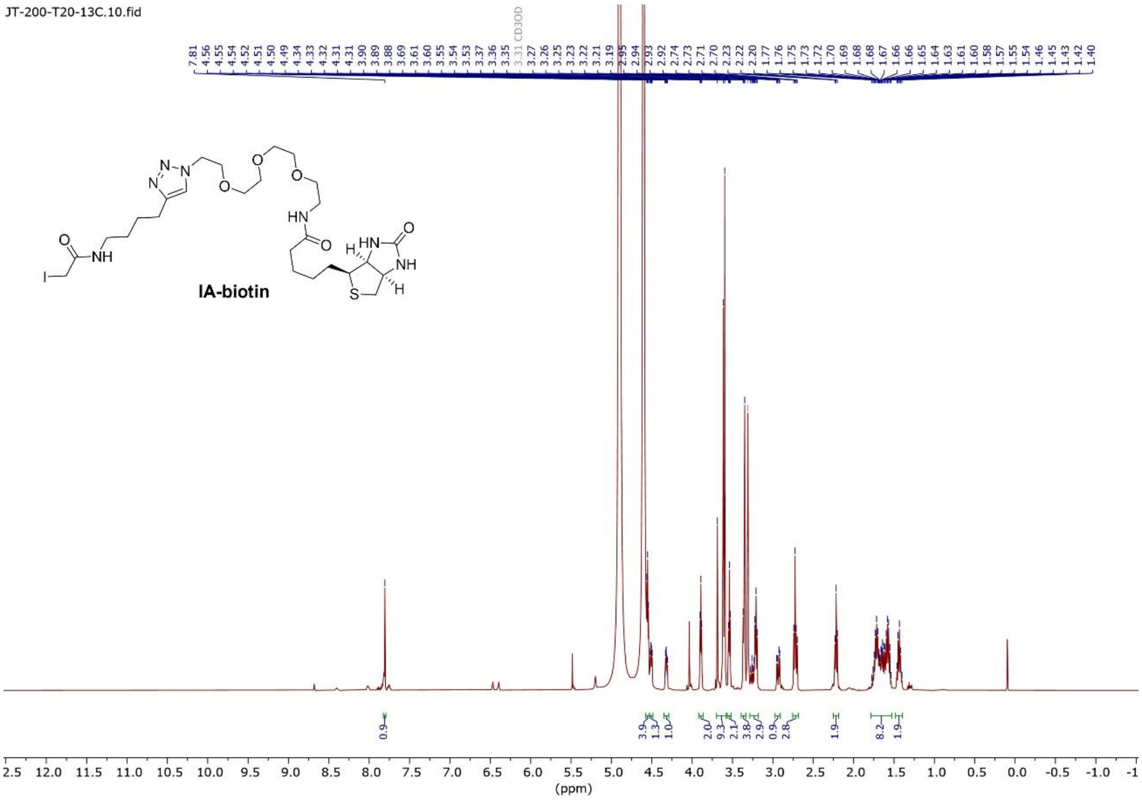

Figure S38. ^{1}H NMR spectra (methanol-*d*4, 500 MHz) of compound **IA-biotin**.

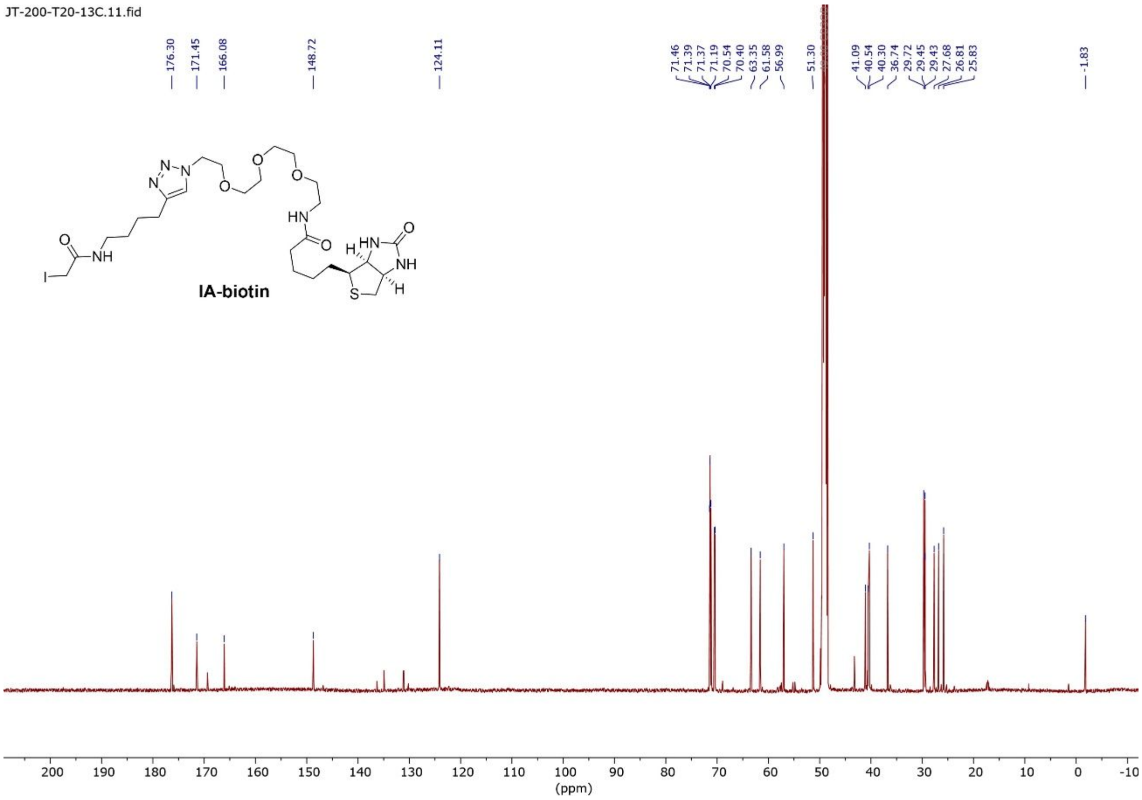

Figure S39. $^{13}C\{^{1}H\}$ NMR spectra (methanol-*d*4, 126 MHz) of compound **IA-biotin**.

HRMS spectra.

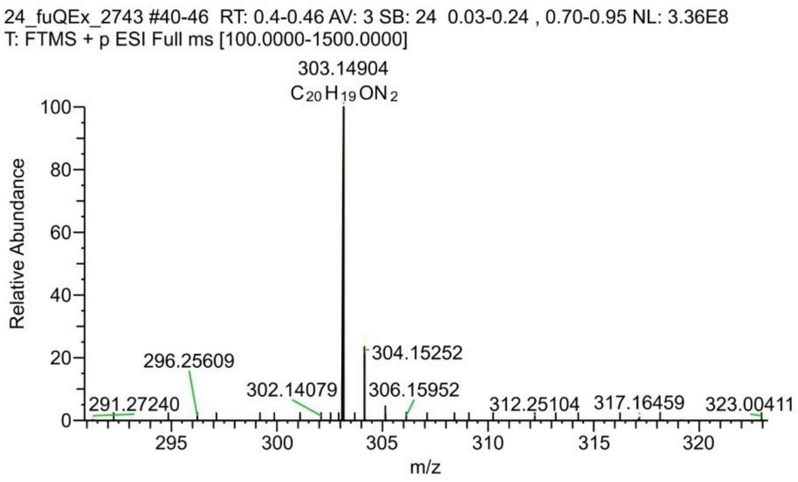

Figure S40. HRESIMS spectrum of compound **1**.

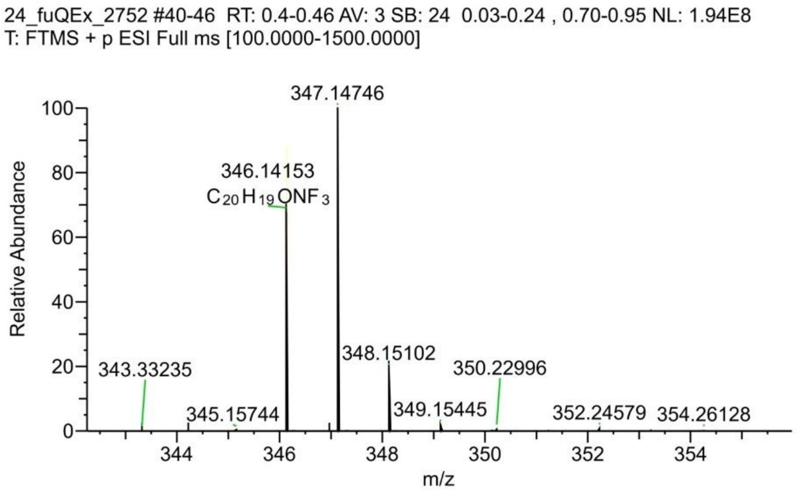

Figure S41. HRESIMS spectrum of compound **2**.

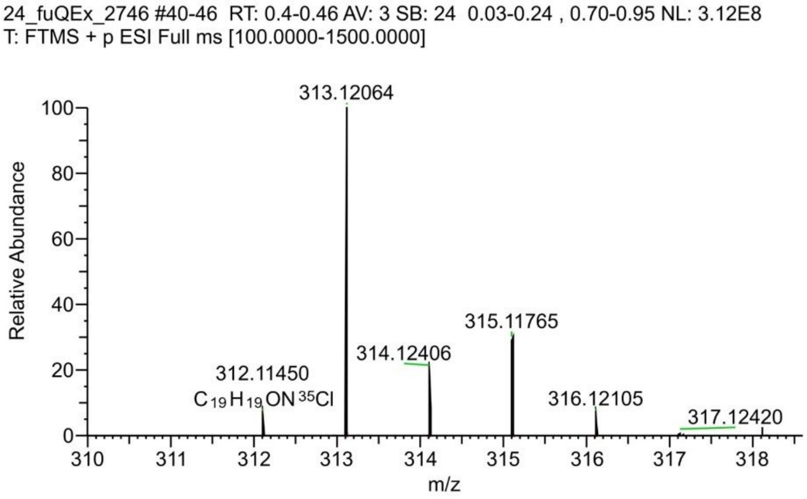

Figure S42. HRESIMS spectrum of compound **3**.

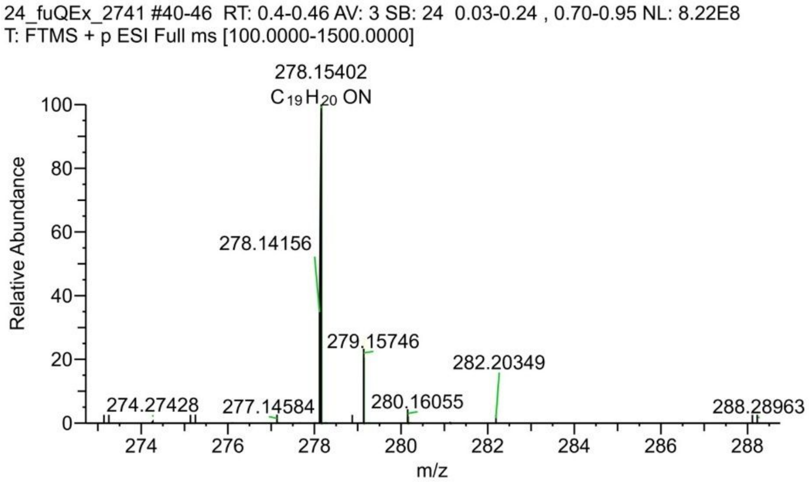

Figure S43. HRESIMS spectrum of compound **4**.

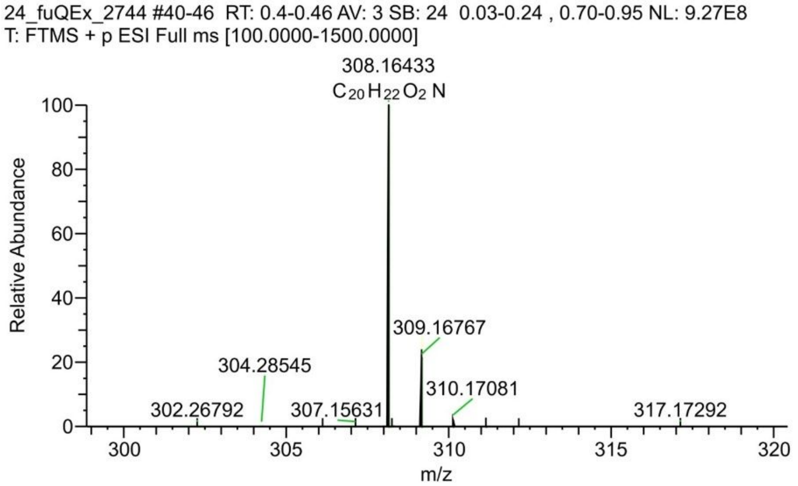

Figure S44. HRESIMS spectrum of compound **5**.

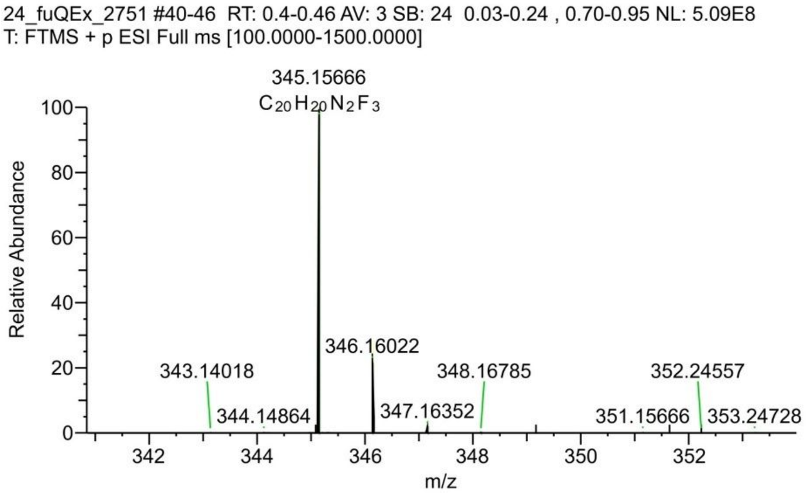

Figure S45. HRESIMS spectrum of compound **2-NH_2**.

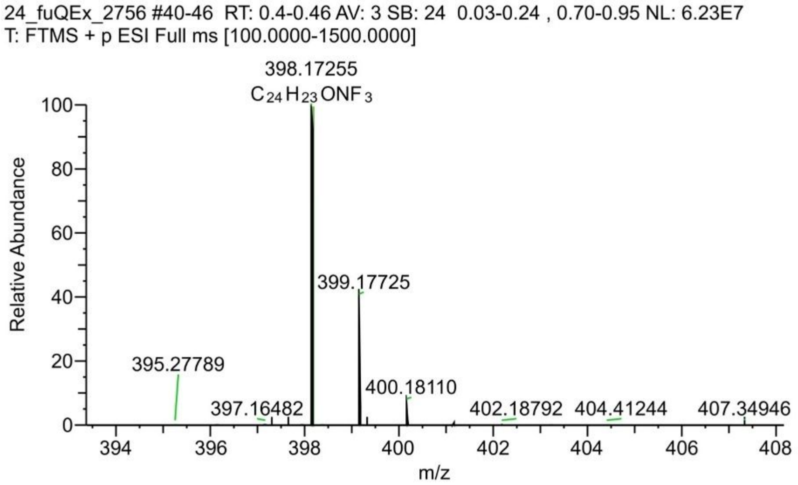

Figure S46. HRESIMS spectrum of compound **2-alkyne**.

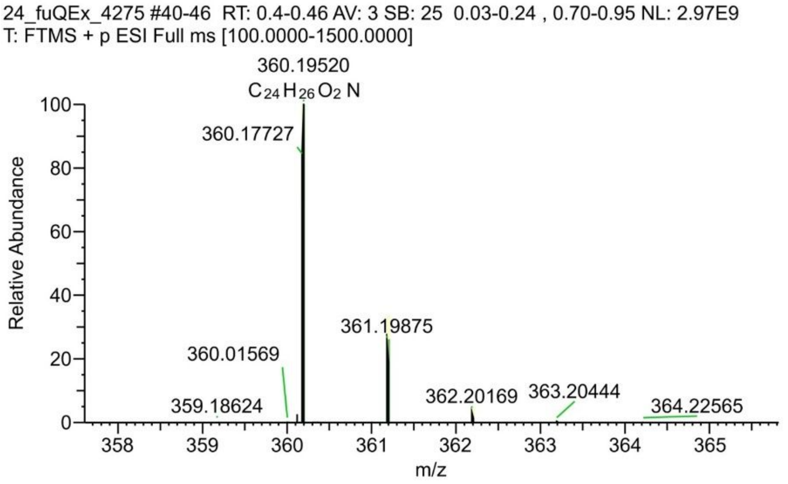

Figure S47. HRESIMS spectrum of compound **5-alkyne**.

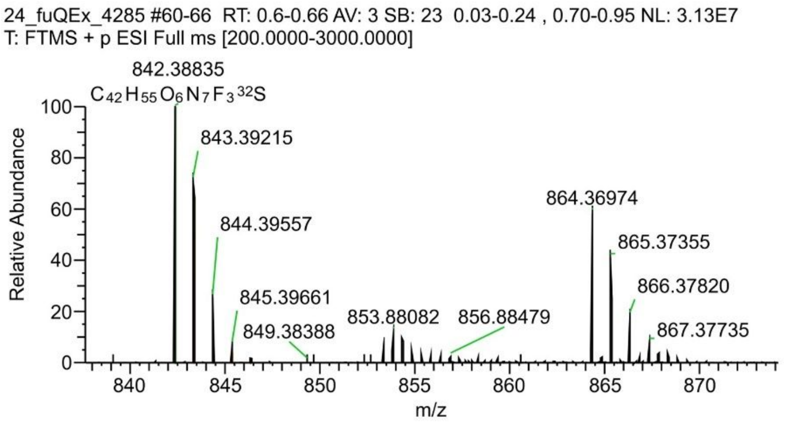

Figure S48. HRESIMS spectrum of compound **2-biotin**.

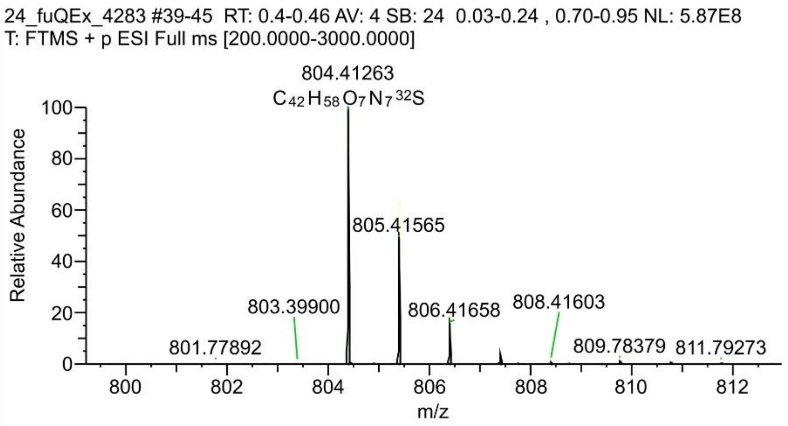

Figure S49. HRESIMS spectrum of compound **5-biotin**.

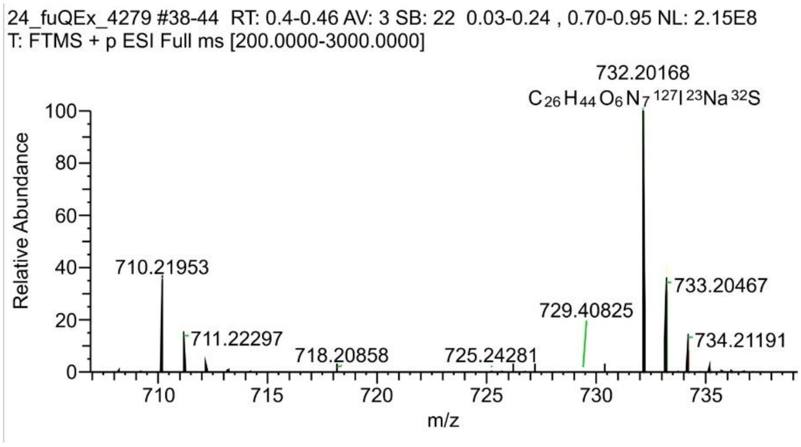

Figure S50. HRESIMS spectrum of compound **IA-biotin**.

Uncropped gels

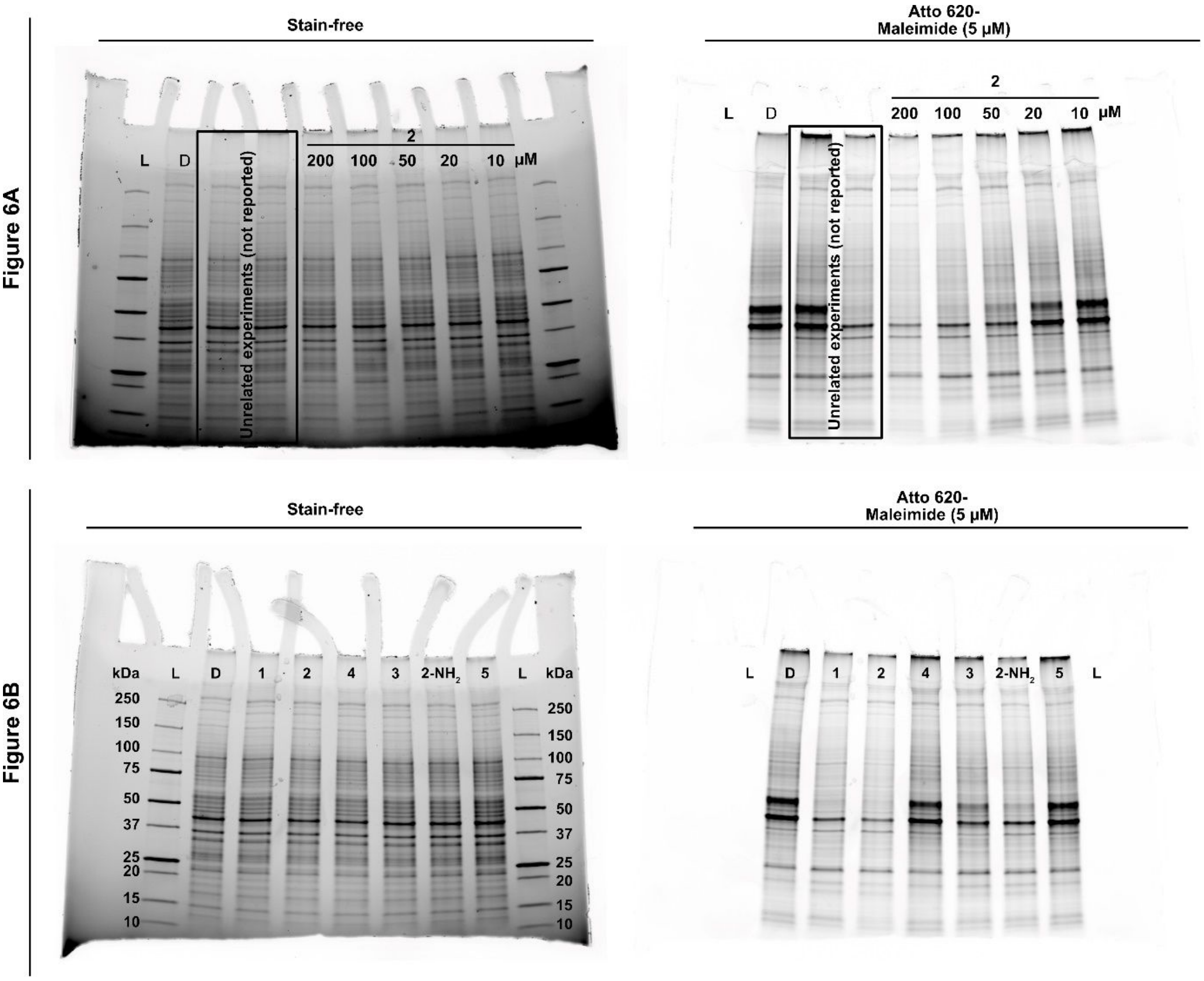

Figure S51. Uncropped gels from the ABPP experiments with compounds **1**-**5** showed in Figure 6.

References.

[1] R. Kar, P. K. Singha, M. A. Venkatachalam, P. Saikumar, *Oncogene* **2009**, *28*, 2556–2568.

[2] D. Nedungadi, A. Binoy, N. Pandurangan, B. G. Nair, N. Mishra, *Cell Biology International* **2021**, *45*, 164–176.

[3] D. Nedungadi, A. Binoy, N. Pandurangan, S. Pal, B. G. Nair, N. Mishra, *Experimental Cell Research* **2018**, *364*, 243–251.

[4] M. J. Yoon, A. R. Lee, S. A. Jeong, Y.-S. Kim, J. Y. Kim, Y.-J. Kwon, K. S. Choi, *Oncotarget* **2014**, *5*, 6816–6831.

[5] M. J. Yoon, Y. J. Kang, J. A. Lee, I. Y. Kim, M. A. Kim, Y. S. Lee, J. H. Park, B. Y. Lee, I. A. Kim, H. S. Kim, S.-A. Kim, A.-R. Yoon, C.-O. Yun, E.-Y. Kim, K. Lee, K. S. Choi, *Cell Death Dis* **2014**, *5*, e1112–e1112.

[6] M. J. Yoon, E. H. Kim, J. H. Lim, T. K. Kwon, K. S. Choi, *Free Radical Biology and Medicine* **2010**, *48*, 713–726.

[7] X. Chen, X. Chen, X. Zhang, L. Wang, P. Cao, V. Rajamanickam, C. Wu, H. Zhou, Y. Cai, G. Liang, Y. Wang, *Redox Biology* **2019**, *21*, 101061.

[8] G.-N. Li, X.-J. Zhao, Z. Wang, M.-S. Luo, S.-N. Shi, D.-M. Yan, H.-Y. Li, J.-H. Liu, Y. Yang, J.-H. Tan, Z.-Y. Zhang, R.-Q. Chen, H.-L. Lai, X.-Y. Huang, J.-F. Zhou, D. Ma, Y. Fang, Q.-L. Gao, *Sig Transduct Target Ther* **2022**, *7*, 317.

[9] M. J. Seo, D. M. Lee, I. Y. Kim, D. Lee, M.-K. Choi, J.-Y. Lee, S. S. Park, S.-Y. Jeong, E. K. Choi, K. S. Choi, *Cell Death Dis* **2019**, *10*, 187.

[10] J. Sang, W. Li, H.-J. Diao, R.-Z. Fan, J.-L. Huang, L. Gan, M.-F. Zou, G.-H. Tang, S. Yin, *Cancer Letters* **2021**, *509*, 13–25.

[11] P. K. Singha, S. Pandeswara, M. A. Venkatachalam, P. Saikumar, *Cell Death Dis* **2013**, *4*, e457–e457.

[12] M. Bury, A. Girault, V. Mégalizzi, S. Spiegl-Kreinecker, V. Mathieu, W. Berger, A. Evidente, A. Kornienko, P. Gailly, C. Vandier, R. Kiss, *Cell Death Dis* **2013**, *4*, e561–e561.

[13] S. H. Kim, H.-Y. Shin, Y.-S. Kim, J. G. Kang, C. S. Kim, S.-H. Ihm, M. G. Choi, H. J. Yoo, S. J. Lee, *Anticancer Res* **2014**, *34*, 4857.

[14] K. Ghosh, S. De, S. Das, S. Mukherjee, S. Sengupta Bandyopadhyay, *PLoS ONE* **2016**, *11*, e0168488.

[15] A. Perry, *Org. Biomol. Chem.* **2019**, *17*, 4825–4834.

[16] V. Demichev, C. B. Messner, S. I. Vernardis, K. S. Lilley, M. Ralser, *Nat Methods* **2020**, *17*, 41–44.

[17] G. Teo, G. Liu, J. Zhang, A. I. Nesvizhskii, A.-C. Gingras, H. Choi, *Journal of Proteomics* **2014**, *100*, 37–43.

[18] W. E. Wolski, P. Nanni, J. Grossmann, M. d'Errico, R. Schlapbach, C. Panse, *J. Proteome Res.* **2023**, *22*, 1092–1104.

[19] J. Nguyen, A. Tirla, P. Rivera-Fuentes, *Org. Biomol. Chem.* **2021**, *19*, 2681–2687.

[20] A. Martin, P. Rivera-Fuentes, *Nat. Chem.* **2024**, *16*, 28–35.
